# Squaramide-Catalyzed Asymmetric Mannich Reaction between 1,3-Dicarbonyl Compounds and Pyrazolinone Ketimines: A Pathway to Enantioenriched 4-Pyrazolyl- and 4-Isoxazolyl-4-aminopyrazolone Derivatives

**DOI:** 10.3390/molecules27206983

**Published:** 2022-10-17

**Authors:** Marta Gil-Ordóñez, Camille Aubry, Cristopher Niño, Alicia Maestro, José M. Andrés

**Affiliations:** GIR SintACat, Instituto CINQUIMA and Departamento de Química Orgánica, Facultad de Ciencias, Universidad de Valladolid, Paseo de Belén 7, 47011 Valladolid, Spain

**Keywords:** asymmetric catalysis, organocatalysis, mannich reaction, ketimines, pyrazolone

## Abstract

A series of N-Boc ketimines derived from pyrazolin-5-ones have been used as electrophiles in enantioselective Mannich reactions with different 1,3-dicarbonyl compounds. This method provides a direct pathway to access the 4-amino-5-pyrazolone derivatives bearing a quaternary substituted stereocenter and containing two privileged structure motifs, the β-diketone and pyrazolinone substructures. The adducts were obtained in excellent yields (up to 90%) and enantioselectivities (up to 94:6 er) by employing a very low loading of 2 mol% of a quinine-derived bifunctional squaramide as an organocatalyst for a wide range of substrates. In addition, the utility of the obtained products was demonstrated through one step transformations to enantioenriched diheterocyclic systems (4-pyrazolyl-pyrazolone and 4-isoxazolyl-pyrazolone), potentially promising candidates for drug discovery.

## 1. Introduction

The enantioselective synthesis of nitrogen-containing heterocycles bearing stereogenic centers has received substantial attention in recent years due to their ubiquity in the cores of natural products and bioactive molecules [1,2,3]. Among the different types of nitrogen-containing heterocycles, pyrazole and pyrazolone derivatives are a privileged class of compounds that possess a broad spectrum of applications as pharmaceutical and agrochemical products, as well as material science [4,5]. Medicinal chemistry researchers have synthesized drug-like pyrazolone candidates that exhibit significant pharmacological activities including antimicrobial, antitumor, CNS (central nervous system) effect, anti-inflammatory activities, and so on. For this reason, significant efforts have been made in recent years to develop new methods for the asymmetric synthesis of the structurally diverse pyrazolone derivatives, especially employing the reactivity of pyrazolin-5-one core [6,7,8,9,10]. However, the asymmetric synthesis of 4,4-disubstituted pyrazolones bearing a nitrogen at C-4 is challenging given the predictable biological activity of these molecules. Several examples are found in the literature that describe the preparation of pyrazolones bearing a tetrasubstituted center via the α-amination reaction of 4-substituted pyrazolones [11,12,13]. Alternatively, the asymmetric reaction of pyrazole-4,5-dione ketimines with different nucleophilic reagents is another straightforward method for the construction of the 4-aminopyrazolone core with a quaternary carbon center. Recently, some organocatalytic asymmetric transformations based on ketimines derived from pyrazolin-5-ones have been reported including Strecker [14] and aza-Friedel Crafts [15,16] reactions.

**Scheme 1:**
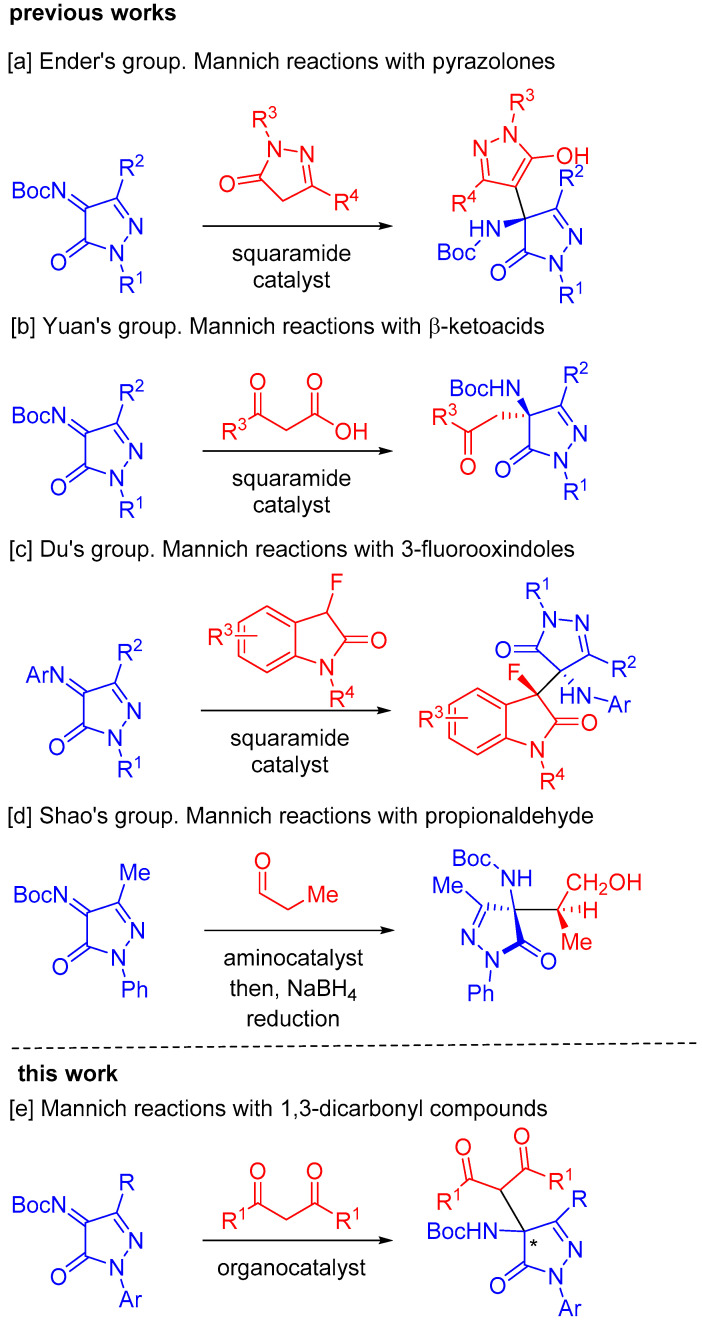
Catalytic asymmetric Mannich reactions of pyrazolinone ketimines.

The asymmetric Mannich reaction is a crucial method for the formation of new C-C bond including β-amino carbonyl compounds [17]. In 2017, Enders′ group was the first to describe the synthesis of a new series of N-Boc ketimines derived from pyrazolin-5-ones and demonstrated their use as electrophiles in asymmetric Mannich reactions with pyrazolones (Scheme 1a) [18]. The reaction proceeded smoothly with 1 mol% quinine-derived squaramide organocatalyst and the desired amino-bis-pyrazolone adducts obtained in excellent yields and stereoselectivities.

Later, Yuan’s group reported the asymmetric decarboxylative Mannich reaction of β-ketoacids with pyrazole-4,5-dione ketimines catalyzed by a quinine-derived squaramide to access chiral β-amino ketone-pyrazolones bearing a tetrasubstituted center at C-4 position in excellent yields and, generally, good enantioselectivities (Scheme 1b) [19]. In 2019, Du and coworkers developed the diastereo- and enantioselective Mannich reaction of 3-fluorooxindole to N-aryl pyrazole-4,5-dione-derived ketimines using a dihydroquinine-derived squaramide as an organocatalyst. The desired products containing an amino-pyrazolone-oxindole scaffold and an asymmetric fluorine atom were obtained in high to excellent yields, with excellent enantio- and good to excellent diastereoselectivities (Scheme 1c) [20]. Unfortunately, the same reaction carried out with N-Boc-protected pyrazolinone ketimine under the optimized reaction conditions provided the target product with low yield and diastereo- and enantioselectivity. In the same year, Shao reported the enantiodivergent Mannich reaction of N-Boc ketimines derived from pyrazolin-5-ones with propionaldehyde promoted by acyclic chiral secondary aminocatalyst leading to the corresponding adducts in good yields with both high diastereoselectivity and enantioselectivity (Scheme 1d) [21].

Herein, we report a new organocatalytic asymmetric Mannich reaction between 1,3-dicarbonyl compounds and ketimines derived from pyrazoline-5-ones to provide 4-amino-5-pyrazolone derivatives bearing a tetra-substituted stereocenter and containing two privileged structure motifs, the β-diketone and pyrazolinone substructures (Scheme 1e).

## 2. Results and Discussion

First, we investigated the reaction of N-Boc ketimine **1a** with 2,4-pentanedione (**2a**) as the model reaction in the presence of 10 mol% of bifunctional organocatalysts **C1** and **C2**, both derived from quinine, in toluene at room temperature (Table 1). With thiourea **C1** as a catalyst, the reaction gave the desired product **3a** in 74% yield with 68:32 er (entry 1). To our delight, squaramide catalyst **C2** provided the product **3a** in excellent yield and with an increase in the er value to 80:20 (entry 2). Screening of different solvents including DCM, CHCl_3_, DCE, Et_2_O, THF, 1,4-dioxane, and ethyl acetate showed that toluene was better than other solvents (entry 2 vs. entries 3–9). In contrast to these results, the reaction in acetonitrile gave the opposite enantiomer with the same enantiomeric ratio (compare entries 2 and 10).

Then, we analyzed the influence of the H-bonding donor group by comparing quinine-derived squaramide **C2** (bearing a phenethyl substituent) with **C3** ((bis(trifluoromethyl)benzyl derivative) and **C4** ((bis(trifluoromethyl)phenyl derivative) squaramides (entries 2 and 11–12). The catalyst **C4** where the squaramide unit is directly attached to an aryl group provided better enantioselectivity (84:16 er) than squaramides **C2** and **C3** in lower reaction time. Additional trials performed under the same reaction conditions with cinchonidine derived-squaramide **C5** and hydroquinine-derived squaramide **C6** did not lead to any increase in enantioselectivity (see entries 13–14). Quinidine-derived squaramide **C7**, a pseudoenantiomer of **C4**, also effectively catalyzed this reaction but gave the opposite enantiomer of **3a** with a similar yield and lower selectivity (71:29 er, entry 15). A significant decrease in enantioselectivity was also observed by using L-valine derived-squaramide **C8** (64:36 er, entry 16).

Next, the catalyst loading of **C4** was reduced to 5 and 2 mol%, and no erosion in chemical yield or enantioselectivity was observed after 2 h of reaction time (entry 18). Lowering the reaction temperature to −18 °C resulted in a longer reaction time and no improvement in the value of er (entry 19). The ratio of nucleophile can be decreased from 2 equivalents to 1.1 equivalents without changing the enantioselectivity (entry 20). In light of the above screening experiments, the best reaction conditions for the enantioselective Mannich reaction were established: 1.1 equiv of diketone in toluene with 2 mol% **C4** at room temperature.

**Scheme 2:**
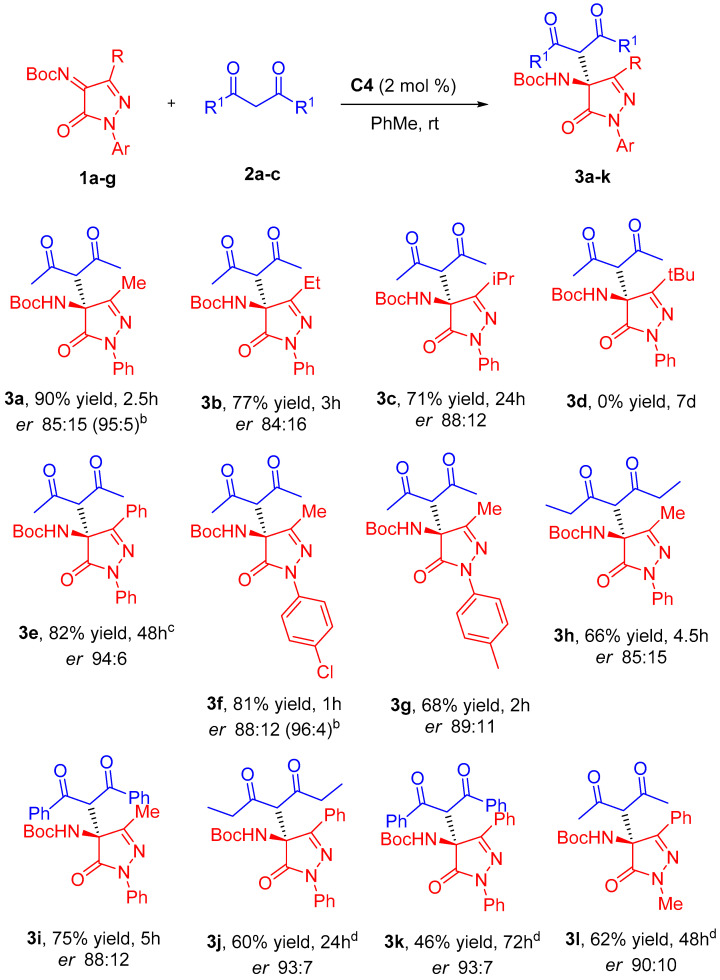
Substrate scope for the asymmetric Mannich reaction. ^a^ Reactions were carried out by using **1** (0.1 mmol), **2** (0.11 mmol), and catalyst **C4** (2 mol%) in 1 mL of PhMe at room temperature. Yields correspond to isolated compound after flash chromatography. The er values were determined by chiral HPLC analysis. ^b^ Determined from the mother liquor after recristallyzation from hexane-EtOAc. ^c^ 5 mol% catalyst was used. ^d^ 10 mol% catalyst was used.

With the optimized reaction conditions in hand, various N-Boc pyrazolinone ketimines **1a**–**g** were reacted with different dicarbonyl compounds **2a**–**c** to produce the corresponding adducts **3a**–**l**. The results are collected in Scheme 2.

The imines **1b** and **1c** bearing ethyl and isopropyl substituent (R) at the C-3 position worked well in the reaction with pentane-2,4-dione and gave the expected products **3b** and **3c** in good yield with 84:16 and 88:12 er, respectively. It was observed that the increase in steric bulk of the alkyl group resulted in lower reactivity, although it provided better enantiocontrol. However, in the reaction of N-Boc ketimine **1d** bearing a tert-butyl group at the C-3 position, no product was observed after seven days of reaction, presumably due to increased steric hindrance. In the case of a phenyl group at the same position, the corresponding product **3e** was obtained with very good yield and enantioselectivity (94:6 er) after 48 h of reaction time. Nevertheless, a small decrease in enantioselectivity (90:10 er) was observed when the N-Boc ketimine **1l**, N-methyl substituted, was reacted with 2,4-pentanedione under similar reaction conditions. When using N-Boc ketimines with different aryl groups at the N-1 position, whether it be electron-withdrawing (**1f**) or electron-donating (**1g**), good yields and slightly higher enantioselectivities were obtained for **3f** and **3g**. It is important to note that recrystallization of adducts **3a** and **3f** from hexane-EtOAc allowed for obtaining enantioenriched **3a** and **3f** (er ≥ 95:5) from the mother liquor in 68% yield.

After exploring a series of pyrazolinone ketimines, the substrate scope of β-diketones was further extended. 3,5-Heptanedione (**2b**) readily reacted with ketimine **1a** leading to **3h** in good yield and moderate enantioselectivity (85:15 er), but the reaction of **1a** with dibenzoylmethane (**2c**) giving **3i** was slower and more enantioselective. Both diketones **2b** and **2c** reacted with the less reactive ketimine **1e** in the presence of 10 mol% catalyst **C4**, providing adducts **3j** and **3k** in moderate yield but with good enantioselectivity (93:7 er).

**Scheme 3:**
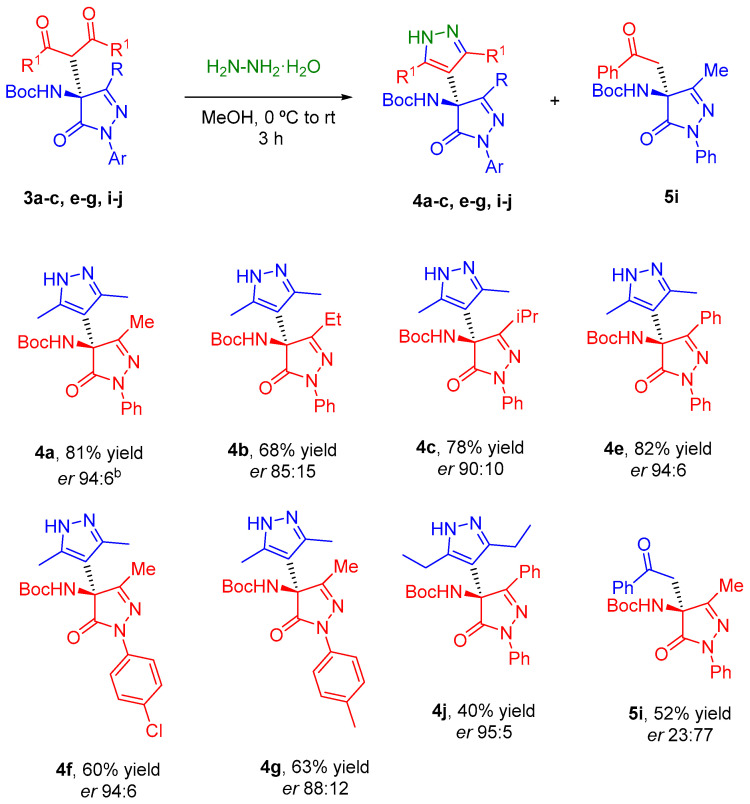
Preparation of pyrazole derivatives **4**. ^a^ Reactions were carried out with H_2_NNH_2_·H_2_O (2 equiv) in MeOH at 0° to rt. Yields correspond to isolated compound after flash chromatography. The er values were determined by chiral HPLC analysis. ^b^ The reactions were performed with enantioenriched **3a** (er 95:5) and **3f** (er 96:4).

Next, the practical synthetic utility of this Mannich reaction was demonstrated by the transformation of adducts **3** into a series of 4-pyrazolyl-pyrazolone derivatives **4** with potential pharmacological interest (Scheme 3).

Condensation of adducts **3a**–**c,e**–**g** with a two-fold excess of hydrazine monohydrate in methanol proceeded easily at room temperature furnishing the pyrazole derivatives **4a**–**c**,**e**–**g** in good yields (60–82%). However, adduct **3j**, prepared from heptane-3,5-dione, reacted under the same reaction conditions to give compound **4j** in only moderate yield (40%). Chiral HPLC analysis of the final pyrazoles **4** showed that the enantiomeric ratio was maintained with respect to the starting compounds, with no erosion of the enantiomeric purity during the transformation. The adduct **4a** was achieved enantiomerically pure (er > 99:1) after recrystallization from hexane-ethyl acetate.

Unexpectedly, in the reaction of dibenzoylmethane derivative **3i** with hydrazine monohydrate, the corresponding condensation product (**4i**) was not the final product; instead, the chiral β-amino ketone-pyrazolinone derivative **5i** was isolated in 52% yield, after cleavage of the benzoyl group of **3i**. This unwanted reaction has not been observed in the reactions of the adducts derived from the pentane-2,4-dione (**3a**–**c**,**e**–**g**) and heptane-3,5-dione (**3j**). Fortunately, the comparison of specific rotation and HPLC retention times of **5i** with those described in literature [19] allowed us to determine the absolute configuration (S) of adduct **3i** by chemical correlation. The absolute configuration of products **3** and **4** is expected to be the same by analogy assuming a common reaction pathway.

**Scheme 4:**
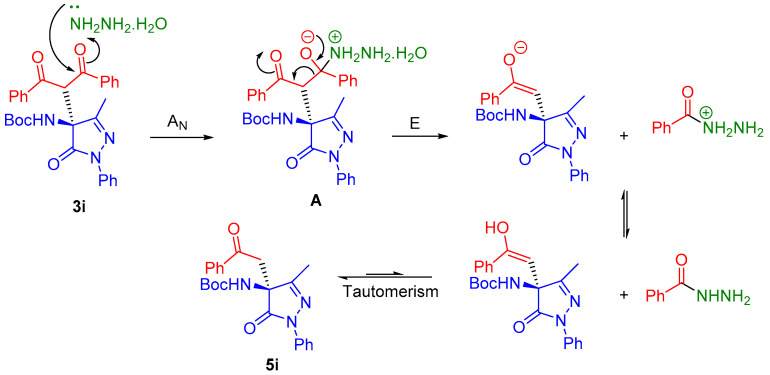
Plausible mechanism of debenzoylation.

A plausible mechanism of this well-known deacylation process [22] is described in Scheme 4. The nucleophilic attack of hydrazine hydrate on the carbonyl group of **3i** leads to intermediate **A**, which undergoes a debenzoylation process to furnish the β-amino ketone-pyrazolinone derivative **5i**.

**Scheme 5:**
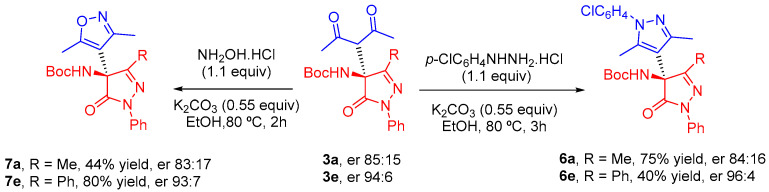
Transformation of adducts **3a,e** to heterocycles **6a**,**e** and **7a**,**e**.

To further illustrate the synthetic potential of this methodology, the asymmetric Mannich addition products **3a**,**e** were treated with 4-chlorophenylhydrazine and hydroxylamine hydrochloride in refluxing ethanol to afford their corresponding 4-chlorophenylpyrazoles (**6a**,**e**) and isoxazoles (**7a**,**e**), respectively, in moderate to good yields (Scheme 5). Again, there is no erosion of the enantiomeric purity during the transformations.

**Figure 1:**
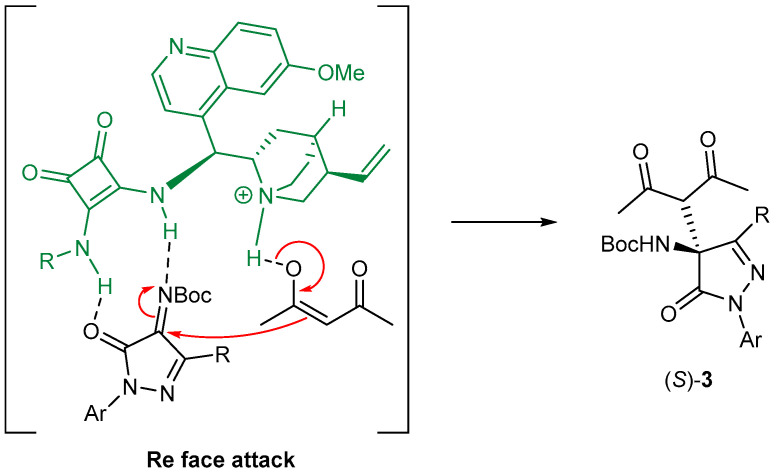
Proposed ternary complex that rationalizes the stereoselection for the Mannich reaction.

In addition, on the basis of our results and those previously reported [18,19], we proposed the formation of the ternary complex depicted in Figure 1 to rationalize the stereochemistry of the products. The H-bonding activation of N-Boc ketimine **1** by the squaramide moiety of catalyst **C4** facilitates the nucleophilic attack of the diketone enolate from the *re*-face of the imine group, leading to the formation of adduct **3** with the (S) configuration.

## 3. Materials and Methods

### 3.1. General Information

^1^H NMR (500 MHz, 400 MHz) and ^13C^ NMR (126 MHz, 101 MHz) spectra were recorded in CDCl_3_ or DMSO-d_6_ as solvent (Laboratory of Instrumental Techniques, University of Valladolid). Chemical shifts for protons are reported in ppm from TMS with the residual CHCl_3_ resonance as internal reference. Chemical shifts for carbons are reported in ppm from TMS and are referenced to the carbon resonance of the solvent. Data are reported as follows: chemical shift, multiplicity (s = singlet, d = doublet, t = triplet, q = quadruplet, quint = quintuplet, sext = sextuplet, sept = septuplet, m = multiplet, br s = broad signal), coupling constants in Hertz, and integration. Specific rotations were measured on a PerkinElmer 341 digital polarimeter using a 5 mL cell with a 1 dm path length, and a sodium lamp, and concentration is given in g per 100 mL. Infrared spectra were recorded on a PerkinElmer Spectrum One FT-IR spectrometer and are reported in frequency of absorption (only the structurally most important peaks are given). Melting points were obtained with a micro melting point Leica Gallen III apparatus and are uncorrected. 

Flash chromatography was carried out using silica gel (230–240 mesh). Chemical yields refer to pure isolated substances. TLC analysis was performed on glass-backed plates coated with silica gel 60 and an F254 indicator, and visualized by either UV irradiation or by staining with phosphomolybdic acid solution. Chiral HPLC analysis was performed on a JASPO HPLC system (JASCO PU-2089 pump and UV-2075 UV/Vis detector) equipped with a quaternary pump, using a Chiralpak AD-H, Chiralpak IA, Lux-Amylose-2 and Lux-i-Amylose-3 analytical columns (250 × 4.6 mm). UV detection was monitored at 254 nm. ESI mass spectra were obtained on an Agilent 5973 inert GC/MS system.

Commercially available organic and inorganic compounds were used without further purification. Solvents were dried and stored over microwave-activated 4 Å molecular sieves. Pyrazolinone ketimines [18], thiourea **C1** [23], and squaramides **C3**–**C7** [24,25,26] were prepared according to literature procedures. Racemic mixture was synthesized according to general procedure using an aquiral bifunctional thiourea derived from *N*^1^,*N*^1^-dimethylethane-1,2-diamine [27] (0.01 mmol) as catalyst. Experimental procedures for the preparation of bifunctional squaramides **C2** and **C8**, NMR Spectra for new compounds and HPLC chromatograms are in the Supplementary Materials.

### 3.2. General Procedure for the Synthesis of Mannich Products **3a**–**k** by Enantioselective Mannich Reaction of N-Boc Ketimines with β-Diketones

To a mixture of N-Boc ketimine **1a**–**g** (0.1 mmol), catalyst **C4** (0.002 mmol, 0.02 equiv) in 1.0 mL of toluene, β-diketone **2a**–**c** (0.11 mmol, 1.1 equiv) was added at room temperature, and the reaction mixture was stirred in a Wheaton vial. The progress of the reaction was monitored by TLC analysis. After the completion of the reaction, the solvent was removed under reduced pressure. The crude reaction mixture was purified by flash column chromatography to afford the corresponding product **3a**–**k**. The enantiomeric excess was determined by chiral-phase HPLC analysis using mixtures of hexane/isopropanol as eluent.

*tert*-Butyl (*S*)-(4-(2,4-dioxopentan-3-yl)-3-methyl-5-oxo-1-phenyl-4,5-dihydro-1*H*-pyrazol-4-yl)carbamate (**3a**). Product **3a** was obtained according to general procedure using pentane-2,4-dione (11 µL, 0.11 mmol, 1.1 equiv) as β-diketone and catalyst **C4** (1.3 mg, 0.002 mmol, 0.02 equiv). Chromatography on a silica gel using hexane/EtOAc = 3:1 as eluent afforded compound **3a** as a colorless solid (35 mg, 0.09 mmol, 90% yield); Mp 140–141 °C (hexane-ethyl acetate); [α]_D_^25^ = +18.9 (*c =* 0.9, CHCl_3_); ^1^H NMR (500 MHz, CDCl_3_): δ 7.87 (dd, *J* = 8.6, 1.2 Hz, 2H, Har), 7.39 (dd, J = 8.6, 7.4 Hz, 2H, Har), 7.19 (tt, J = 7.4, 1.3 Hz, 1H, Har), 6.38 (br s, 1H, NH), 4.08 (s, 1H, CH), 2.31 (s, 3H, CH_3_CO), 2.30 (s, 3H, CH_3_CO), 2.08 (s, 3H, CH_3_), 1.36 (s, 9H, C(CH_3_)_3_) ppm; ^13^C NMR (126 MHz, CDCl_3_): δ 200.4 (CO), 169.9 (CON), 137.7 (Car), 128.9 (CHar), 125.4 (CHar), 118.9 (CHar), 77.3 (C(CH_3_)_3_), 66.9 (CH), 66.7 (CNHBoc), 32.1 (CH_3_CO), 31.9 (CH_3_CO), 28.1 (C(CH_3_)_3_), and 14.8 (CH_3_) ppm. IR (ATR): 3403, 3356, 2974, 2931, 1707, 1596, 1496, 1375, 1254, 1154, 758, 688 cm^−1^. HRMS (ESI-QTOF) m/z: [M+H]^+^ Calcd. For C_20_H_26_N_3_O_5_ 388.1867; Found 388.1868. Chiral HPLC analysis: Chiralpak AD-H column, hexane/*i*-PrOH 85:15, 0.7 mL/min, λ = 254 nm, major enantiomer (*S*) *t*_R_ = 11.5 min, minor enantiomer (*R*) *t*_R_ = 26.1 min. (*er* 85:15). 

A sample of **3a** (*er* 85:15) was recrystallized from MeOH to afford **3a** as white crystals (quasi-racemic mixture, *er* 58:42) and enantioenriched **3a** from the mother liquors (*er* 95:5). This last fraction was then used to prepare compound **4a**.

*tert*-Butyl (*S*)-(4-(2,4-dioxopentan-3-yl)-3-ethyl-5-oxo-1-phenyl-4,5-dihydro-1*H*-pyrazol-4-yl)carbamate (**3b**). Product **3b** was obtained according to general procedure using pentane-2,4-dione (11 µL, 0.11 mmol, 1.1 equiv) as β-diketone and catalyst **C4** (1.3 mg, 0.002 mmol, 0.02 equiv). Chromatography on a silica gel using hexane/EtOAc = 3:1 as eluent afforded compound **3b** as a colorless solid (31 mg, 0.077 mmol, 77% yield). [α]_D_^25^ = +10.7 (*c =* 0.5, CHCl_3_). ^1^H NMR (500 MHz, CDCl_3_): δ 7.89 (dd, *J* = 8.7, 1.2 Hz, 2H, Har), 7.38 (dd, *J* = 8.7, 7.4 Hz, 2H, Har), 7.18 (tt, J = 7.4, 1.2 Hz, 1H, Har), 6.40 (br s, 1H, NH), 4.05 (s, 1H, CH), 2.41 (m, 1H, CHHCH_3_), 2.34 (m, 1H, CHHCH_3_), 2.30 (s, 3H, CH_3_CO), 2.29 (s, 3H, CH_3_CO), 1.34 (s, 9H, C(CH_3_)_3_), 1.27 (t, *J* = 7.3 Hz, 3H, CH_3_CH_2_) ppm. ^13^C NMR (126 MHz, CDCl_3_): δ 200.7 (CO), 170.1 (CON), 137.9 (Car), 128.8 (CHar), 125.3 (CHar), 118.9 (CHar), 77.3 (C(CH_3_)_3_), 67.1 (CH), 66.9 (CNHBoc), 32.1 (CH_3_CO), 31.9 (CH_3_CO), 28.1 (C(CH_3_)_3_), 22.2 (CH_2_CH_3_), 9.6 (CH_3_CH_2_) ppm. IR (ATR): 3388, 2985, 2942, 1707, 1596, 1493, 1453, 1351, 1279, 1152, 1054, 761, 692 cm^−1^. HRMS (ESI-QTOF) m/z: [M+H]^+^ Calcd. For C_21_H_28_N_3_O_5_ 402.2023; Found 402.2029. Chiral HPLC analysis: Chiralpak AD-H column, hexane/*i*-PrOH 85:15, 0.7 mL/min, λ = 254 nm, major enantiomer (*S*) *t*_R_ = 10.0 min, minor enantiomer (*R*) *t*_R_ = 20.0 min. (*er* 84:16).

*tert*-Butyl (*S*)-(4-(2,4-dioxopentan-3-yl)-3-isopropyl-5-oxo-1-phenyl-4,5-dihydro-1*H*-pyrazol-4-yl)carbamate (**3c**). Product **3c** was obtained according to general procedure using pentane-2,4-dione (11 µL, 0.11 mmol, 1.1 equiv) as β-diketone and catalyst **C4** (1.3 mg, 0.002 mmol, 0.02 equiv). Chromatography on a silica gel using hexane/EtOAc = 4:1 as eluent afforded compound **3c** as a colorless solid (30 mg, 0.071 mmol, 71% yield). [α]_D_^25^ = +42.5 (*c =* 0.8, MeOH). ^1^H NMR (500 MHz, CDCl_3_): δ 7.89 (d, *J* = 8.1 Hz, 2H, Har), 7.38 (dd, J = 8.7, 7.4 Hz, 2H, Har), 7.17 (tt, J = 7.4, 1.2 Hz, 1H, Har), 6.49 (br s, 1H, NH), 4.04 (s, 1H, CH), 2.65 (sept, J = 6.9 Hz, 1H, CH(CH_3_)_2_), 2.29 (s, 3H, CH_3_CO), 2.28 (s, 3H, CH_3_CO), 1.37 (s, 9H, C(CH_3_)_3_), 1.27 (d, *J* = 6.8 Hz, 6H, (CH_3_)_2_CH) 1.24 (d, *J* = 7.0 Hz, 6H, (CH_3_)_2_CH) ppm. ^13^C NMR (126 MHz, CDCl_3_): δ 201.1 (CO), 169.8 (CON), 138.0 (Car), 128.8 (CHar), 125.2 (CHar), 119.1 (CHar), 77.3 (C(CH_3_)_3_), 67.4 (CH(COCH_3_)_2_), 67.0 (CNHBoc), 32.1 (CH_3_CO), 31.7 (CH_3_CO), 28.8 (CH(CH_3_)_2_), 28.1 (C(CH_3_)_3_), 20.3 ((CH_3_)_2_CH) ppm. IR (ATR): 3413, 2975, 2935, 1710, 1598, 1493, 1457, 1359, 1283, 1156, 1083, 1054, 754, 688 cm^−1^. HRMS (ESI-QTOF) m/z: [M+Na]^+^ Calcd. For C_22_H_29_N_3_NaO_5_ 438.1999; Found 438.1999. Chiral HPLC analysis: Chiralpak AD-H column, hexane/*i*-PrOH 95:5, 0.7 mL/min, λ = 254 nm, major enantiomer (*S*) *t*_R_ = 18.6 min, minor enantiomer (*R*) *t*_R_ = 27.0 min. (*er* 88:12).

*tert*-Butyl (*S*)-(4-(2,4-dioxopentan-3-yl)-5-oxo-1,3-diphenyl-4,5-dihydro-1*H*-pyrazol-4-yl)carbamate (**3e**). Product **3e** was obtained according to general procedure using pentane-2,4-dione (11 µL, 0.11 mmol, 1.1 equiv) as β-diketone and catalyst **C4** (3.2 mg, 0.005 mmol, 0.05 equiv). Chromatography on a silica gel using hexane/EtOAc = 4:1 as eluent afforded compound **3e** as a colorless solid (37 mg, 0.082 mmol, 82% yield). [α]_D_^25^ = +56.3 (*c =* 0.6, CHCl_3_). ^1^H NMR (500 MHz, CDCl_3_): δ 7.98 (dd, J = 8.4, 1.2 Hz, 2H, Har), 7.90 (dd, *J* = 7.8, 2.0 Hz, 2H, Har), 7.43 (m, 5H, Har), 7.23 (tt, J = 7.4, 1.0 Hz, 1H, Har), 6.82 (br s, 1H, NH), 3.97 (s, 1H, CH), 2.11 (s, 3H, CH_3_CO), 2.03 (s, 3H, CH_3_CO), 1.31 (s, 9H, C(CH_3_)_3_) ppm. ^13^C NMR (126 MHz, CDCl_3_): δ 202.2 (CO), 200.5 (CO), 170.0 (CON), 137.9 (Car), 130.8 (CHar), 129.0 (CHar), 128.9 (CHar), 126.7 (CHar), 125.6 (CHar), 119.2 (CHar), 77.3 (C(CH_3_)_3_), 66.3 (CH), 66.2 (CNHBoc), 32.3 (CH_3_CO), 32.2 (CH_3_CO), 28.1 (C(CH_3_)_3_) ppm. IR (ATR): 3379, 2982, 1714, 1597, 1490, 1358, 1255, 1156, 751, 689 cm^−1^. HRMS (ESI-QTOF) m/z: [M+Na]^+^ Calcd. For C_25_H_27_N_3_NaO_5_ 472.1843; Found 472.1842. Chiral HPLC analysis: Chiralpak AD-H column, hexane/*i*-PrOH 85:15, 0.7 mL/min, λ = 254 nm, major enantiomer (*S*) *t*_R_ = 12.7 min, minor enantiomer (*R*) *t*_R_ = 29.1 min. (*er* 94:6).

*tert*-Butyl (*S*)-(1-(4-chlorophenyl)-4-(2,4-dioxopentan-3-yl)-3-methyl-5-oxo-4,5-dihydro-1*H*-pyrazol-4-yl)carbamate (**3f**). Product **3f** was obtained according to general procedure using pentane-2,4-dione (11 µL, 0.11 mmol, 1.1 equiv) as β-diketone and catalyst **C4** (1.3 mg, 0.002 mmol, 0.02 equiv). Chromatography on a silica gel using hexane/EtOAc = 3:1 as eluent afforded compound **3f** as a colorless solid (34 mg, 0.081 mmol, 81% yield). Mp 170–171 °C (hexane-ethyl acetate). [α]_D_^25^ = +17.9 (*c =* 0.6, CHCl_3_). ^1^H NMR (500 MHz, CDCl_3_): δ 7.84 (d, *J* = 8.9 Hz, 2H, Har), 7.34 (d, *J* = 8.9 Hz, 2H, Har), 6.36 (br s, 1H, NH), 4.05 (s, 1H, CH), 2.30 (s, 3H, CH_3_CO), 2.29 (s, 3H, CH_3_CO), 2.07 (s, 3H, CH_3_), 1.35 (s, 9H, C(CH_3_)_3_) ppm. ^13^C NMR (126 MHz, CDCl_3_): δ 200.4 (CO), 169.9 (CON), 158.1 (CO_2_tBu), 153.5 (CCH_3_), 136.3 (Car), 130.4 (Car), 128.9 (CHar), 119.9 (CHar), 81.7 (C(CH_3_)_3_), 66.9 (CH), 66.6 (CNHBoc), 32.1 (CH_3_CO), 31.9 (CH_3_CO), 28.1 (C(CH_3_)_3_), 14.7 (CH_3_) ppm. IR (ATR): 3419, 2975, 2905, 1714, 1494, 1461, 1365, 1251, 1152, 836, 810 cm^−1^. HRMS (ESI-QTOF) m/z: [M+H]^+^ Calcd. ForC_20_H_25_ClN_3_O_5_ 422.1477; Found 422.1487. Chiral HPLC analysis: Chiralpak AD-H column, hexane/*i*-PrOH 85:15, 0.7 mL/min, λ = 254 nm, major enantiomer (*S*) *t*_R_ = 10.0 min, minor enantiomer (*R*) *t*_R_ = 31.8 min. (*er* 88:12). A sample of **3f** (*er* 88:12) was recrystallized from hexane-ethyl acetate to afford **3f** as white crystals (quasi-racemic mixture, *er* 58:42) and enantioenriched **3f** from the mother liquors (*er* 96:4). This last fraction was then used to prepare compound **4f**.

*tert*-Butyl (*S*)-(4-(2,4-dioxopentan-3-yl)-3-methyl-5-oxo-1-(*p*-tolyl)-4,5-dihydro-1*H*-pyrazol-4-yl)carbamate (**3g**). Product **3g** was obtained according to general procedure using pentane-2,4-dione (11 µL, 0.11 mmol, 1.1 equiv) as β-diketone and catalyst **C4** (1.3 mg, 0.002 mmol, 0.02 equiv). Chromatography on a silica gel using hexane/EtOAc = 3:1 as eluent afforded compound **3g** as a colorless solid (27 mg, 0.068 mmol, 68% yield). Mp 150–151 °C (hexane-ethyl acetate). [α]_D_^25^ = +19.0 (*c =* 0.5, CHCl_3_). ^1^H NMR (500 MHz, CDCl_3_): δ 7.73 (d, *J* = 8.4 Hz, 2H, Har), 7.18 (d, *J* = 8.4 Hz, 2H, Har), 6.35 (br s, 1H, NH), 4.07 (s, 1H, CH), 2.33 (s, 3H, CH_3_C_6_H_4_), 2.30 (s, 3H, CH_3_CO), 2.29 (s, 3H, CH_3_CO), 2.07 (s, 3H, CH_3_), 1.35 (s, 9H, C(CH_3_)_3_) ppm. ^13^C NMR (126 MHz, CDCl_3_): δ 200.4 (CO), 169.7 (CON), 135.3 (Car), 135.1 (Car), 129.4 (CHar), 119.0 (CHar), 77.3 (C(CH_3_)_3_), 67.0 (CH), 66.7 (CNHBoc), 32.1 (CH_3_CO), 31.9 (CH_3_CO), 28.1 (C(CH_3_)_3_), 21.0 (CH_3_C_6_H_4_), 14.8 (CH_3_) ppm. IR (ATR): 3423, 2978, 2923, 1714, 1703, 1512, 1472, 1369, 1255, 1156, 1056, 814 cm^−1^. HRMS (ESI-QTOF) m/z: [M+H]^+^ Calcd. For C_21_H_28_N_3_O_5_ 402.2023; Found 402.2043. Chiral HPLC analysis: Chiralpak AD-H column, hexane/*i*-PrOH 85:15, 0.7 mL/min, λ = 254 nm, major enantiomer (*S*) *t*_R_ = 11.5 min, minor enantiomer (*R*) *t*_R_ = 44.7 min. (*er* 89:11).

*tert*-Butyl (*S*)-(4-(3,5-dioxoheptan-4-yl)-3-methyl-5-oxo-1-phenyl-4,5-dihydro-1*H*-pyrazol-4-yl)carbamate (**3h**). Product **3h** was obtained according to general procedure using heptane-3,5-dione (15 µL, 0.11 mmol, 1.1 equiv) as β-diketone and catalyst **C4** (1.3 mg, 0.002 mmol, 0.02 equiv). Chromatography on a silica gel using hexane/EtOAc = 4:1 as eluent afforded compound **3h** as a colorless solid (27 mg, 0.066 mmol, 66% yield). Mp 119–120 °C (hexane-ethyl acetate). [α]_D_^25^ = +12.9 (*c =* 0.6, CHCl_3_). ^1^H NMR (500 MHz, CDCl_3_): δ 7.85 (dd, *J* = 8.7, 1.2 Hz, 2H, Har), 7.37 (dd, *J* = 8.6, 7.4 Hz, 2H, Har), 7.17 (tt, *J* = 7.4, 1,2 Hz, 1H, Har), 6.55 (br s, 1H, NH), 4.03 (s, 1H, CH), 2.60 (m, 2H, CHHCH_3_), 2.54 (m, 2H, CHHCH_3_), 2.05 (s, 3H, CH_3_), 1.36 (s, 9H, C(CH_3_)_3_), 1.08 (t, *J* = 7.1 Hz, 3H, CH_3_CH_2_), 0.99 (t, *J* = 7.1 Hz, 3H, CH_3_CH_2_) ppm. ^13^C NMR (126 MHz, CDCl_3_): δ 202.7 (CO), 170.1 (CON), 137.8 (Car), 128.8 (CHar), 125.3 (CHar), 118.9 (CHar), 77.3 (C(CH_3_)_3_), 67.0 (CH), 65.1 (CNHBoc), 38.6 (CH_2_CO), 38.4 (CH_2_CO), 28.1 (C(CH_3_)_3_), 14.8 (CH_3_), 7.4 (CH_3_CH_2_), 7.3 (CH_3_CH_2_) ppm. IR (ATR): 3339, 2978, 2942, 1714, 1597, 1497, 1369, 1270, 1152, 1104, 759, 689 cm^−1^. HRMS (ESI-QTOF) m/z: [M+H]^+^ Calcd. For C_22_H_30_N_3_O_5_ 416.2180; Found 416.2189. Chiral HPLC analysis: Chiralpak AD-H column, hexane/*i*-PrOH 85:15, 0.7 mL/min, λ = 254 nm, major enantiomer (*S*) *t*_R_ = 9.2 min, minor enantiomer (*R*) *t*_R_ = 23.8 min. (*er* 85:15).

*tert*-Butyl (*S*)-(4-(1,3-dioxo-1,3-diphenylpropan-2-yl)-3-methyl-5-oxo-1-phenyl-4,5-dihydro-1*H*-pyrazol-4-yl)carbamate (**3i**). Product **3i** was obtained according to general procedure using 1,3-diphenylpropane-1,3-dione (25 mg, 0.11 mmol, 1.1 equiv) as β-diketone and catalyst **C4** (1.3 mg, 0.002 mmol, 0.02 equiv). Chromatography on a silica gel using hexane/EtOAc = 4:1 as eluent afforded compound **3i** as a colorless solid (38 mg, 0.075 mmol, 75% yield). Mp 201-202 °C (hexane-ethyl acetate). [α]_D_^25^ = -68.6 (*c =* 0.7, CHCl_3_). ^1^H NMR (500 MHz, CDCl_3_): δ 7.89 (dd, *J* = 11.9, 8.6 Hz, 4H, Har), 7.51 (m, 4H, Har), 7.39 (m, 4H, Har), 7.22 (dd, *J* = 8.6, 7.2 Hz, 2H, Har), 7.07 (td, *J* = 7.4, 1.3 Hz, 1H, Har), 6.61 (br s, 1H, NH), 5.91 (s, 1H, CH), 2.22 (s, 3H, CH_3_), 1.36 (s, 9H, C(CH_3_)_3_) ppm. ^13^C NMR (126 MHz, CDCl_3_): δ 191.1 (CO), 170.1 (CON), 137.5 (Car), 136.1 (Car), 135.2 (Car), 134.5 (CHar), 134.2 (CHar), 129.1 (CHar), 129.1 (CHar), 128.7 (CHar), 128.5 (CHar), 128.5 (CHar), 125.0 (CHar), 118.6 (CHar), 77.3 (C(CH_3_)_3_), 67.8 (CNHBoc), 56.6 (CH), 28.1 (C(CH_3_)_3_), 15.8 (CH_3_) ppm. IR (ATR): 3276, 3147, 2986, 1729, 1700, 1593, 1490, 1446, 1365, 1270, 1214, 1152, 770, 751, 696, 682 cm^−1^. HRMS (ESI-QTOF) m/z: [M+H]^+^ Calcd. For C_30_H_30_N_3_O_5_ 512.2180; Found 512.2214. Chiral HPLC analysis: Chiralpak AD-H column, hexane/*i*-PrOH 85:15, 0.7 mL/min, λ = 254 nm, major enantiomer (*S*) *t*_R_ = 14.2 min, minor enantiomer (*R*) *t*_R_ = 35.8 min. (*er* 88:12).

*tert*-Butyl (*S*)-(4-(3,5-dioxoheptan-4-yl)-5-oxo-1,3-diphenyl-4,5-dihydro-1*H*-pyrazol-4-yl)carbamate (**3j**). Product **3j** was obtained according to general procedure using heptane-3,5-dione (15 µL, 0.11 mmol, 1.1 equiv) as β-diketone and catalyst **C4** (6.3 mg, 0.01 mmol, 0.1 equiv). Chromatography on a silica gel using hexane/EtOAc = 8:1 as eluent afforded compound **3j** as a colorless solid (29 mg, 0.06 mmol, 60% yield). [α]_D_^25^ = +48.4 (*c =* 0.5, CHCl_3_). ^1^H NMR (500 MHz, CDCl_3_): δ 7.98 (dd, J = 8.6, 1.3 Hz, 2H, Har), 7.88 (dd, *J* = 7.3, 2.5 Hz, 2H, Har), 7.43 (m, 5H, Har), 7.23 (m, 1H, Har), 7.12 (br s, 1H, NH), 3.94 (s, 1H, CH), 2.59 (dq, J = 20.6, 7.1 Hz, 1H, CHHCH_3_), 2.38 (dq, J = 19.0, 7.0 Hz, 1H, CHHCH_3_), 2.19 (dq, J = 19.2, 7.1 Hz, 1H, CHHCH_3_), 2.03 (dq, J = 19.4, 7.0 Hz, 1H, CHHCH_3_), 1.33 (s, 9H, C(CH_3_)_3_), 0.90 (t, J = 7.1 Hz, 3H, CH_3_), 0.79 (t, J = 7.0 Hz, 3H, CH_3_) ppm. ^13^C NMR (126 MHz, CDCl_3_): δ 204.0 (CO), 202.7 (CO), 170.2 (CON), 137.9 (Car), 130.7 (CHar), 128.9 (CHar), 126.8 (CHar), 125.5 (CHar), 119.2 (CHar), 77.3 (C(CH_3_)_3_), 66.7 (CNHBoc), 39.0 (2 CH_2_), 28.1 (C(CH_3_)_3_), 7.2 (CH_3_), 6.9 (CH_3_) ppm. IR (ATR): 3369, 2979, 2939, 1731, 1698, 1599, 1489, 1397, 1283, 1158, 1114, 1015, 758, 736, 689 cm^−1^. HRMS (ESI-QTOF) m/z: [M+H]^+^ Calcd. For C_27_H_32_N_3_O_5_ 478.2336; Found 478.2345. Chiral HPLC analysis: Chiralpak AD-H column, hexane/*i*-PrOH 85:15, 0.7 mL/min, λ = 254 nm, major enantiomer (*S*) *t*_R_ = 8.9 min, minor enantiomer (*R*) *t*_R_ = 22.3 min. (*er* 93:7).

*tert*-Butyl (*S*)-(4-(1,3-dioxo-1,3-diphenylpropan-2-yl)-5-oxo-1,3-diphenyl-4,5-dihydro-1*H*-pyrazol-4-yl)carbamate (**3k**). Product **3k** was obtained according to general procedure using 1,3-diphenylpropane-1,3-dione (25 mg, 0.11 mmol, 1.1 equiv) as β-diketone and catalyst **C4** (6.3 mg, 0.01 mmol, 0.1 equiv). Chromatography on a silica gel using hexane/EtOAc = 4:1 as an eluent afforded compound **3k** as a colorless solid (26 mg, 0.046 mmol, 46% yield). Mp 189–190 °C (hexane-EtOAc). [α]_D_^25^ = +41.7 (*c =* 0.34, CHCl_3_). ^1^H NMR (500 MHz, CDCl_3_): δ 7.88 (ddd, *J* = 8.6, 7.6, 1.3 Hz, 4H, Har), 7.75 (d, *J* = 7.2 Hz, 2H, Har), 7.58 (m, 1H, Har), 7.49 (dd, *J* = 8.5, 1.3 Hz, 2H, Har), 7.40 (m, 5H, Har), 7.21 (m, 6H, Har), 5.80 (s, 1H, CH), 1.39 (s, 9H, C(CH_3_)_3_) ppm. ^13^C NMR (126 MHz, CDCl_3_): δ 190.4 (CO), 170.3 (CON), 137.9 (Car), 137.0 (Car), 136.6 (Car), 134.1 (CHar), 133.8 (CHar), 130.4 (CHar), 129.1 (CHar), 128.9 (CHar), 128.6 (CHar), 128.4 (CHar), 128.2 (CHar), 128.1 (CHar), 127.5 (CHar), 125.4 (CHar), 118.9 (CHar), 77.3 (C(CH_3_)_3_), 68.6 (CNHBoc), 51.9 (CH), 28.2 ((CH_3_)_3_C) ppm. IR (ATR): 3420, 3068, 2979, 2928, 1709, 1695, 1595, 1482, 1280, 1258, 1159, 971, 758, 685 cm^−1^. HRMS (ESI-QTOF) m/z: [M+H]^+^ Calcd. For C_35_H_32_N_3_O_5_ 574.2336; Found 574.2342. Chiral HPLC analysis: Chiralpak AD-H column, hexane/*i*-PrOH 80:20, 1 mL/min, λ = 254 nm, major enantiomer (*S*) *t*_R_ = 10.9 min, minor enantiomer (*R*) *t*_R_ = 23.8 min. (*er* 93:7).

*tert*-Butyl (*S*)-(4-(2,4-dioxopentan-3-yl)-1-methyl-5-oxo-3-phenyl-4,5-dihydro-1*H*-pyrazol-4-yl)carbamate (**3l**)**.** Product **3l** was obtained according to general procedure using pentane-2,4-dione (11 µL, 0.11 mmol, 1.1 equiv) as β-diketone and catalyst **C4** (6.4 mg, 0.01 mmol, 0.1 equiv). Chromatography on a silica gel using hexane/EtOAc = 4:1 as eluent afforded compound **3l** as a colorless solid (24 mg, 0.062 mmol, 62% yield). [α]_D_^25^ = +46.0 (*c =* 0.3, CHCl_3_). ^1^H NMR (500 MHz, CDCl_3_): δ 7.77 (m, 2H, Har), 7.40 (m, 3H, Har), 6.76 (br s, 1H, NH), 3.86 (s, 1H, CH), 3.45 (s, 1H, CH_3_N), 2.07 (s, 3H, CH_3_CO), 2.03 (s, 3H, CH_3_CO), 1.36 (br s, 9H, C(CH_3_)_3_) ppm. ^13^C NMR (126 MHz, CDCl_3_): δ 202.4 (CO), 200.5 (CO), 171.4 (CON), 133.9 (Car), 130.4 (CHar), 128.9 (CHar), 126.4 (CHar), 77.2 (C(CH_3_)_3_), 66.0 (CH), 64.9 (CNHBoc), 32.2 (CH_3_N), 32.2 (CH_3_CO), 28.1 (C(CH_3_)_3_) ppm. IR (ATR): 3375, 2978, 2926, 1703, 1483, 1351, 1252, 1157, 765, 695 cm^−1^. HRMS (ESI-QTOF) m/z: [M+Na]^+^ Calcd. For C_20_H_25_N_3_NaO_5_ 410.1686; Found 410.1686. Chiral HPLC analysis: Chiralpak AD-H column, hexane/*i*-PrOH 95:5, 0.8 mL/min, λ = 254 nm, major enantiomer (*S*) *t*_R_ = 31.6 min, minor enantiomer (*R*) *t*_R_ = 43.1 min. (*er* 90:10).

### 3.3. General Procedure for the Synthesis of Pyrazole Derivatives **4a**–**j** by Reaction of Adducts **3a**–**j** with Hydrazine Hydrate

To a solution of adduct **3** (0.1 mmol) in 1.0 mL of methanol, hydrated hydrazine (12 µL, 0.2 mmol, 2 equiv) was added at 0 °C, and the reaction mixture was then stirred at rt. The progress of the reaction was monitored by TLC analysis. After the completion of the reaction, the solvent was removed under reduced pressure. The crude reaction mixture was purified by flash column chromatography to afford the corresponding product **4**. The enantiomeric excess was determined by chiral-phase HPLC analysis using mixtures of hexane/isopropanol as eluent.

*tert*-Butyl (*S*)-(3,3′,5-trimethyl-5′-oxo-1′-phenyl-1′,5′-dihydro-1*H*,4′*H*-[4,4′-bipyrazol]-4′-yl)carbamate (**4a**). Product **4a** was obtained from **3a** according to general procedure. Chromatography on a silica gel using EtOAc as eluent afforded compound **4a** as a colorless solid (31 mg, 0.081 mmol, 81% yield). Mp 220–222 °C (hexane-ethyl acetate). [α]_D_^25^ = +96.5 (*c =* 0.5, CHCl_3_). ^1^H NMR (400 MHz, CDCl_3_): δ 7.93 (dd, J = 8.0, 0.8 Hz, 2H, Har), 7.39 (t, J = 7.8 Hz, 2H, Har), 7.18 (tt, *J* = 7.4, 1.2 Hz, 1H, Har), 5.92 (br s, 1H, NH), 2.28 (s, 6H, CH_3_), 2.11 (s, 3H, CH_3_), 1.37 (s, 9H, C(CH_3_)_3_) ppm. ^13^C NMR (126 MHz, CDCl_3_): δ 172.0 (CON), 160.5 (CO_2_tBu), 154.1 (CCH_3_), 142.3 (CCH_3_), 138.0 (Car), 128.9 (CHar), 125.0 (CHar), 118.6 (CHar), 107.5 (C_4pyrazole_), 77.3 (C(CH_3_)_3_), 65.6 (CNHBoc), 28.1 (C(CH_3_)_3_), 14.2 (CH_3_), 12.9 (CH_3_) ppm. IR (ATR): 3247, 2978, 2934, 1711, 1692, 1593, 1501, 1365, 1251, 1156, 759, 693 cm^−1^. HRMS (ESI-QTOF) m/z: [M+Na]^+^ Calcd. For C_20_H_25_N_5_NaO_3_ 406.1850; Found 406.1860. Chiral HPLC analysis: Lux Amylose-2 column, hexane/*i*-PrOH 90:10, 1 mL/min, λ = 254 nm, minor enantiomer (*R*) *t*_R_ = 19.9 min, major enantiomer (*S*) *t*_R_ = 29.1 min. (*er* 94:6).

*tert*-Butyl (*S*)-(3′-ethyl-3,5-dimethyl-5′-oxo-1′-phenyl-1′,5′-dihydro-1*H*,4′*H*-[4,4′-bipyrazol]-4′-yl)carbamate (**4b**). Product **4b** was obtained from **3b** according to general procedure. Chromatography on a silica gel using EtOAc as eluent afforded compound **4b** as a colorless solid (27 mg, 0.068 mmol, 68% yield). [α]_D_^25^ = +62.8 (*c =* 0.14, CHCl_3_). ^1^H NMR (500 MHz, CDCl_3_): δ 7.97 (dd, *J* = 8.7, 1.2 Hz, 2H, Har), 7.40 (dd, *J* = 8.7, 7.4 Hz, 2H, Har), 7.18 (tt, *J* = 7.4, 1.2 Hz, 1H, Har), 5.71 (br s, 1H, NH), 2.50 (dq, *J* = 17.6, 7.4 Hz, 1H, CHHCH_3_), 2.37 (m, 1H, CHHCH_3_), 2.28 (s, 6H, CH_3_), 1.36 (s, 9H, C(CH_3_)_3_), 1.30 (t, J = 7.0 Hz, 3H, CH_3_CH_2_) ppm. ^13^C NMR (126 MHz, CDCl_3_): 172.1 (CON), 164.0 (CO_2_tBu), 154.0 (CEt), 142.3 (CCH_3_), 138.2 (Car), 128.8 (CHar), 124.9 (CHar), 118.5 (CHar), 108.0 (C_4pyrazole_), 77.2 (C(CH_3_)_3_), 65.6 (CNHBoc), 28.1 (C(CH_3_)_3_), 21.5 (CH_2_CH_3_), 12.9 (CH_3_), 9.1 (CH_3_CH_2_) ppm. HRMS (ESI-QTOF) m/z: [M+H]^+^ Calcd. For C_21_H_28_N_5_O_3_ 398.2187; Found 398.216. Chiral HPLC analysis: Chiralpak AD-H column, hexane/*i*-PrOH 90:10, 1 mL/min, λ = 254 nm, major enantiomer (*S*) *t*_R_ = 25.8 min, minor enantiomer (*R*) *t*_R_ = 31.8 min. (*er* 85:15). 

*tert*-Butyl (*S*)-(3′-isopropyl-3,5-dimethyl-5′-oxo-1′-phenyl-1′,5′-dihydro-1*H*,4′*H*-[4,4′-bipyrazol]-4′-yl)carbamate (**4c**). Product **4c** was obtained from **3c** according to general procedure. Chromatography on a silica gel using hexane/EtOAc = 2:1 as an eluent afforded compound **4c** as a colorless solid (32 mg, 0.078 mmol, 78% yield). [α]_D_^25^ = +139.8 (*c =* 0.46, CHCl_3_). ^1^H NMR (500 MHz, CDCl_3_): δ 7.99 (d, J = 7.1 Hz, 2H, Har), 7.40 (dd, J = 8.5, 7.4 Hz, 2H, Har), 7.18 (t, *J* = 7.4 Hz, 1H, Har), 6.22 (br s, 1H, NH), 2.66 (sept, *J* = 6.8 Hz, 1H, CH(CH_3_)_2_), 2.23 (s, 6H, CH_3_), 1.36 (s, 9H, C(CH_3_)_3_), 1.32 (d, *J* = 7.0 Hz, 3H, CH_3_CH), 1.07 (d, *J* = 6.8 Hz, 3H, CH_3_CH) ppm. ^13^C NMR (126 MHz, CDCl_3_): 172.3 (CON), 167.0 (CO_2_tBu), 154.4 (CiPr), 141.9 (CCH_3_), 138.1 (Car), 128.8 (CHar), 125.0 (CHar), 118.6 (CHar), 107.8 (C_4pyrazole_), 77.2 (C(CH_3_)_3_), 66.2 (CNHBoc), 28.2 (CH(CH_3_)_2_), 28.1 (C(CH_3_)_3_), 21.1 (CH_3_CH), 20.8 (CH_3_CH), 12.8 (CH_3_) ppm. IR (ATR): 3290, 2975, 2931, 1708, 1597, 1494, 1367, 1159, 759, 737, 693 cm^−1^. HRMS (ESI-QTOF) m/z: [M+Na]^+^ Calcd. For C_22_H_29_N_5_NaO_3_ 434.2163; Found 434.2162. Chiral HPLC analysis: Lux Amylose-2 column, hexane/*i*-PrOH 90:10, 1 mL/min, λ = 254 nm, minor enantiomer (*R*) *t*_R_ = 17.5 min, major enantiomer (*S*) *t*_R_ = 23.5 min. (*er* 90:10).

*tert*-Butyl (*S*)-(3,5-dimethyl-5′-oxo-1′,3′-diphenyl-1′,5′-dihydro-1*H*,4′*H*-[4,4′-bipyrazol]-4′-yl)carbamate (**4e**). Product **4e** was obtained from **3e** according to general procedure. Chromatography on a silica gel using hexane/EtOAc = 1:1 as eluent afforded compound **4e** as a colorless solid (36 mg, 0.082 mmol, 82% yield). [α]_D_^25^ = −190.0 (*c =* 0.1, CHCl_3_). ^1^H NMR (500 MHz, CDCl_3_): δ 8.13 (br s, 1H, NH), 7.99 (dd, J = 8.7, 1.2 Hz, 2H, Har), 7.85 (d, J = 7.1 Hz, 2H, Har), 7.39 (m, 5H, Har), 7.19 (tt, J = 7.4, 1.2 Hz, 1H, Har), 2.30 (s, 6H, CH_3_), 1.19 (s, 9H, C(CH_3_)_3_) ppm. ^13^C NMR (126 MHz, CDCl_3_): δ 171.5 (CON), 167.0 (CO_2_tBu), 153.7 (CPh), 143.1 (CCH_3_), 138.3 (Car), 138.2 (Car), 128.9 (CHar), 128.8 (CHar), 126.4 (CHar), 125.1 (CHar), 118.7 (CHar), 108.7 (C_4pyrazole_), 77.2 (C(CH_3_)_3_), 64.1 (CNHBoc), 27.9 (C(CH_3_)_3_), 12.8 (CH_3_) ppm. IR (ATR): 3237, 3123, 3060, 2978, 2931, 1730, 1708, 1594, 1500, 1367, 1159, 759, 737, 689 cm^−1^. HRMS (ESI-QTOF) m/z: [M+H]^+^ Calcd. For C_25_H_28_N_5_O_3_ 446.2187; Found 446.2205. Chiral HPLC analysis: Lux *i*-Amylose-3 column, hexane/*i*-PrOH 90:10, 1 mL/min, λ = 254 nm, major enantiomer (*S*) *t*_R_ = 12.8 min, minor enantiomer (*R*) *t*_R_ = 32.5 min. (*er* 94:6).

*tert*-Butyl (*S*)-(1′-(4-chlorophenyl)-3,3′,5-trimethyl-5′-oxo-1′,5′-dihydro-1*H*,4′*H*-[4,4′-bipyrazol]-4′-yl)carbamate (**4f**). Product **4f** was obtained from enantioenriched **3f** according to general procedure. Chromatography on a silica gel using hexane/EtOAc = 1:3 as eluent afforded compound **4f** as a colorless solid (25 mg, 0.060 mmol, 60% yield). [α]_D_^25^ = +51.0 (*c =* 0.22, CHCl_3_)**.** ^1^H NMR (500 MHz, CDCl_3_): δ 7.91 (d, *J* = 8.9 Hz, 2H, Har), 7.35 (d, *J* = 8.9 Hz, 2H, Har), 5.84 (br s, 1H, NH), 2.27 (s, 6H, CH_3_), 2.10 (s, 3H, CH_3_), 1.37 (s, 9H, C(CH_3_)_3_) ppm. ^13^C NMR (126 MHz, CDCl_3_): δ 171.9 (CON), 160.7 (CO_2_tBu), 154.0 (CMe), 142.3 (CCH_3pyrazole_), 136.6 (Car), 130.1 (Car), 128.9 (CHar), 119.6 (CHar), 107.4 (C_4pyrazole_), 77.2 (C(CH_3_)_3_), 65.5 (CNHBoc), 28.1 (C(CH_3_)_3_), 14.2 (CH_3_), 12.8 (CH_3_) ppm. IR (ATR): 3268, 2978, 2931, 1708, 1490, 1361, 1254, 1159, 1093, 1011, 910, 828, 727 cm^−1^. HRM**S** (ESI-QTOF) m/z: [M+H]^+^ Calcd. For C_20_H_25_ClN_5_O_3_ 418.1640; Found 418.1633. HPLC: Lux *i*-Amylose-3 column, hexane/*i*-PrOH 90:10, 1 mL/min, λ = 254 nm, major enantiomer (*S*) t_R_ = 26.5 min, minor enantiomer (*R*) *t*_R_ = 52.5 min. (*er* 94:6).

*tert*-Butyl (*S*)-(3,3′,5-trimethyl-5′-oxo-1′-(*p*-tolyl)-1′,5′-dihydro-1*H*,4′*H*-[4,4′-bipyrazol]-4′-yl)carbamate (**4g**). Product **4g** was obtained from **3g** according to general procedure. Chromatography on a silica gel using hexane/EtOAc = 1:3 as an eluent afforded compound **4g** as a colorless solid (25 mg, 0.063 mmol, 63% yield). [α]_D_^25^ = +61.2 (c = 0.3, CHCl_3_). ^1^H NMR (500 MHz, CDCl_3_): δ 7.80 (d, J = 8.6 Hz, 2H, Har), 7.19 (d, J = 8.6 Hz, 2H, Har), 6.00 (br s, 1H, NH), 2.34 (s, 3H, CH_3_C_4_H_6_), 2.26 (s, 6H, CH_3_), 2.09 (s, 3H, CH_3_), 1.37 (s, 9H, C(CH_3_)_3_) ppm. ^13^C NMR (126 MHz, CDCl_3_): δ 171.8 (CON), 160.5 (CO_2_tBu), 154.1 (CCH_3_), 142.3 (CCH_3pyrazole_), 135.6 (Car), 134.7 (Car), 129.4 (CHar), 118.6 (CHar), 107.6 (C_4pyrazole_), 77.3 (C(CH_3_)_3_), 65.5 (CNHBoc), 28.1 (C(CH_3_)_3_), 20.9 (CH_3_C_6_H_4_), 14.1 (CH_3_), 12.8 (CH_3_) ppm. IR (ATR): 3268, 2982, 2928, 1705, 1509, 1361, 1250, 1159, 815, 730 cm^−1^. HRMS (ESI-QTOF) m/z: [M+H]^+^ Calcd. For C_21_H_28_N_5_O_3_ 398.2187; Found 398.2183. Chiral HPLC analysis: Chiralpak IA, hexane/*i*-PrOH 90:10, 1 mL/min, λ = 254 nm, major enantiomer (*S*) *t*_R_ = 23.5 min, minor enantiomer (*S*) *t*_R_ = 47.2 min. (*er* 88:12).

*tert*-Butyl (S)-(3,5-diethyl-5′-oxo-1′,3′-diphenyl-1′,5′-dihydro-1*H*,4′*H*-[4,4′-bipyrazol]-4′-yl)carbamate (**4j**). Product **4j** was obtained from **3j** according to general procedure. Chromatography on a silica gel using hexane/EtOAc = 2:1 as eluent afforded compound **4j** as a colorless solid (19 mg, 0.040 mmol, 40% yield). [α]_D_^25^ = −134.3 (*c =* 0.2, CHCl_3_). ^1^H NMR (500 MHz, CDCl_3_): δ 7.97 (dd, J = 8.6, 1.2 Hz, 2H, Har), 7.82 (d, *J* = 7.6 Hz, 2H, Har), 7.42 (m, 5H, Har), 7.21 (tt, J = 7.4, 1.2 Hz, 1H, Har), 2.98 (m, 2H, CHHCH_3_), 2.86 (dq, J = 15.5, 7.6 Hz, CHHCH_3_), 1.24 (t, J = 7.5, 6H), 1.20 (s, 9H, C(CH_3_)_3_ ppm. ^13^C NMR (126 MHz, CDCl_3_): δ 166.1 (CON), 148.5 (CPh), 144.0 (CEt), 133.4 (Car), 124.3 (CHar), 124.2 (CHar), 121.7 (CHar), 120.6 (CHar), 114.1 (CHar), 104.4 (C_4pyrazole_), 72.3 (C(CH_3_)_3_), 23.2 (C(CH_3_)_3_), 15.1 (CH_3_CH_2_), 8.7 (CH_3_CH_2_), 7.3 (CH_3_CH_2_) ppm. IR (ATR): 3250, 2975, 2928, 1701, 1594, 1490, 1368, 1159, 756, 693 cm^−1^. HRMS (ESI-QTOF) m/z: [M+H]^+^ Calcd. For C_27_H_32_N_5_O_3_ 474.2500; Found 474.2486. Chiral HPLC analysis: Chiralpak IA, hexane/*i*-PrOH 95:5, 1 mL/min, λ = 254 nm, minor enantiomer (*R*) *t*_R_ = 20.1 min, major enantiomer (*S*) *t*_R_ = 25.0 min. (*er* 95:5).

*tert*-Butyl (*S*)-(3-methyl-5-oxo-4-(2-oxo-2-phenylethyl)-1-phenyl-4,5-dihydro-1*H*-pyrazol-4-yl)carbamate (**5i**) [19]. Product **5i** was obtained from **3i** according to general procedure. Chromatography on a silica gel using hexane/EtOAc = 3:1 as eluent afforded compound **5i** as a colorless solid (21 mg, 0.052 mmol, 52% yield). [α]_D_^25^ = −17.5 (*c =* 0.3, CH_2_Cl_2_). [Lit. [19] [α]_D_^20^ = −20.2 (*c* = 1, CH_2_Cl_2_, er 94:6 for (*S*) enantiomer)]. ^1^H NMR (400 MHz, DMSO-d_6_): δ 7.90 (br s, 1H, Har), 7.83 (m, 2H, Har), 7.74 (d, J = 7.8 Hz, 2H, Har), 7.62 (tt, J = 7.4, 1.3 Hz, 1H, Har), 7.49 (t, J = 7.8 Hz, 2H, Har), 7.38 (dd, J = 8.7, 7.4 Hz, 2H, Har), 7.14 (tt, J = 7.4, 1.3 Hz, 1H, Har), 3.74 (d, J = 17.2 Hz, 1H, CHHCOPh), 3.62 (d, J = 17.2 Hz, 1H, CHHCOPh), 1.99 (s, 3H, CH_3_), 1.31 (s, 9H, C(CH_3_)_3_) ppm. ^13^C NMR (100 MHz, DMSO-d_6_): δ 195 (CO), 172.3 (CON), 158.8 (CO_2_tBu), 153.8 (CCH_3_), 138.7 (Car), 136.2 (Car), 134.2 (CHar), 129.3 (CHar), 129.2 (CHar), 128.4 (CHar), 124.7 (CHar), 118.1 (CHar), 80.0 (C(CH_3_)_3_), 63.6 (CNHBoc), 42.6 (CH_2_), 28.4 (C(CH_3_)_3_), 13.5 (CH_3_) ppm. IR (ATR): 2856, 1714, 1594, 1500, 1364, 1251, 1159, 753, 693 cm^−1^. HRMS (ESI-QTOF) m/z: [M+Na]^+^ Calcd. For C_35_H_25_N_3_NaO_4_ 430.1737; Found 430.1759. Chiral HPLC analysis: Chiralpak IA column, hexane/*i*-PrOH 80:20, 1 mL/min, λ = 254 nm, minor enantiomer (*R*) *t*_R_ = 6.9 min, major enantiomer (*S*) *t*_R_ = 32.4 min. (*er* 77:23).

### 3.4. General Procedure for the Synthesis of Pyrazole Derivatives **6a**,**e**

A solution of **3a,e** (0.1 mmol), 4-chlorophenylhydrazine hydrochloride (19 mg, 0.11 mmol, 1.1 equiv) and and K_2_CO_3_ (8 mg, 0.055 mmol, 0.55 equiv) in ethanol (1 mL) was heated to 80 °C for 2–3 h. After that, the solvent of reaction mixture was removed under reduced pressure. The crude product was purified by flash chromatography on silica gel to afford **6a**,**e**.

*tert*-Butyl (S)-(1-(4-chlorophenyl)-3,3′,5-trimethyl-5′-oxo-1′-phenyl-1′,5′-dihydro-1*H*,4′*H*-[4,4′-bipyrazol]-4′-yl)carbamate (**6a**). Product **6a** was obtained from **3a** according to general procedure. Chromatography on a silica gel using hexane/EtOAc = 4:1 as an eluent afforded compound **6a** as a colorless solid (37 mg, 0.075 mmol, 75% yield). Mp 196–197 °C (hexane-ethyl acetate). [α]_D_^25^ = +59.9 (*c =* 0.7, CHCl_3_). ^1^H NMR (500 MHz, CDCl_3_): δ 7.94 (dd, J = 8.7, 1.2 Hz, 2H, Har), 7.40 (m, 4H, Har), 7.28 (d, *J* = 8.5 Hz, 2H, Har), 7.18 (tt, J = 7.4, 1.2 Hz, 1H, Har), 5.41 (br s, 1H, NH), 2.40 (s, 3H, CH_3_), 2.33 (s, 3H, CH_3_), 2.19 (s, 3H, CH_3_), 1.39 (s, 9H, C(CH_3_)_3_) ppm. ^13^C NMR (126 MHz, CDCl_3_): δ 171.5 (CON), 159.8 (CO_2_tBu), 153.7 (CCH_3_), 146.6 (CCH_3_), 138.0 (Car), 137.1 (Car), 134.4 (Car), 129.4 (CHar), 128.9 (CHar), 127.1 (CHar), 125.1 (CHar), 118.6 (CHar), 110.0 (C_4pyrazole_), 79.7 (C(CH_3_)_3_), 65.4 (CNHBoc), 28.1 (C(CH_3_)_3_), 14.5 (CH_3_), 14.1 (CH_3_), 12.3 (CH_3_) ppm. IR (ATR): 3269, 2982, 2928, 1711, 1598, 1500, 1393, 1364, 1295, 1254, 1163, 1093, 1014, 838, 759, 690, 645 cm^−1^. HRMS (ESI-QTOF) m/z: [M+H]^+^ Calcd. For C_26_H_29_ClN_5_O_3_ 494.1953; Found 494.1931. Chiral HPLC analysis: Chiralpak AD-H column, hexane/*i*-PrOH 90:10, 1 mL/min, λ = 254 nm, major enantiomer (*S*) *t*_R_ = 45.0 min, minor enantiomer (*R*) *t*_R_ = 74.2 min. (*er* 84:16).

*tert*-Butyl (*S*)-(1-(4-chlorophenyl)-3,5-dimethyl-5′-oxo-1′,3′-diphenyl-1′,5′-dihydro-1H,4′H-[4,4′-bipyrazol]-4′-yl)carbamate (**6e**). Product **6e** was obtained from **3e** according to general procedure. Chromatography on a silica gel using hexane/EtOAc = 4:1 as eluent afforded compound **6e** as a colorless solid (22 mg, 0.040 mmol, 40% yield). [α]_D_^25^ = −155.0 (*c =* 0.4, CHCl_3_). ^1^H NMR (500 MHz, CDCl_3_): δ 8.01 (dd, J = 8.8, 1.1 Hz, 2H, Har), 7.91 (d, J = 6.5 Hz, 2H, Har), 7.44 (m, 5H, Har), 7.42 (d, J = 8.7 Hz, 2H, Har), 7.29 (d, J = 8.7 Hz, 2H, Har), 7.20 (tt, J = 7.4, 1.2 Hz, 1H, Har), 5.54 (br s, 1H, NH), 2.41 (s, 3H, CH_3_), 2.31 (s, 3H, CH_3_), 1.22 (s, 9H, C(CH_3_)_3_) ppm. ^13^C NMR (126 MHz, CDCl_3_): δ 171.7 (CON), 153.6 (CCH_3_), 146.9 (CPh), 138.4 (Car), 137.5 (Car), 134.1 (Car), 130.7 (CCH_3_), 130.3 (Car), 129.3 (CHar), 128.9 (CHar), 126.9 (CHar), 126.5 (CHar), 125.1 (CHar), 118.9 (CHar), 110.5 (C_4pyrazole_), 77.2 (C(CH_3_)_3_), 64.4 (CNHBoc), 27.9 (C(CH_3_)_3_), 14.2 (CH_3_), 12.6 (CH_3_) ppm. IR (ATR): 3245, 2975, 2854, 1727, 1701, 1596, 1500, 1362, 1260, 1158, 1092, 1016, 829, 756, 735, 691 cm^−1^. HRMS (ESI-QTOF) m/z: [M+Na]^+^ Calcd. For C_31_H_30_N_5_ClNaO_3_ 578.1929; Found 578.1943. Chiral HPLC analysis: Chiralpak AD-H column, hexane/*i*-PrOH 90:10, 1 mL/min, λ = 254 nm, major enantiomer (*S*) *t*_R_ = 10.1 min, minor enantiomer (*R*) *t*_R_ = 63.7 min. (*er* 96:4).

### 3.5. General Procedure for the Synthesis of Isoxazole Derivatives **7a**,**e**

A solution of **3a,e** (0.1 mmol), hydroxylamine hydrochloride (8 mg, 0.11 mmol, 1.1 equiv) and K_2_CO_3_ (8 mg, 0.055 mmol, 0.55 equiv) in ethanol (1 mL) was heated to 80 °C for 2–3 h. After that, the solvent of reaction mixture was removed under reduced pressure. The crude product was purified by flash chromatography on silica gel to afford **7a**,**e**.

*tert*-Butyl (*S*)-(4-(3,5-dimethylisoxazol-4-yl)-3-methyl-5-oxo-1-phenyl-4,5-dihydro-1*H*-pyrazol-4-yl)carbamate (**7a**). Product **7a** was obtained from **3a** according to general procedure. Chromatography on a silica gel using hexane/EtOAc = 3:1 as an eluent afforded compound **7a** as a colorless solid (17 mg, 0.044 mmol, 44% yield). [α]_D_^25^ = +45.2 (*c =* 0.5, CHCl_3_). ^1^H NMR (500 MHz, CDCl_3_): δ 7.91 (dd, J = 8.8, 1.1 Hz, 2H, Har), 7.41 (dd, J = 8.7, 7.4 Hz, 2H, Har), 7.20 (tt, J = 7.4, 1.2 Hz, 1H, Har), 5.35 (br s, 1H, NH), 2.47 (s, 3H, CH_3_), 2.36 (s, 3H, CH_3_), 2.15 (s, 3H, CH_3_), 1.38 (s, 9H, C(CH_3_)_3_) ppm. ^13^C NMR (126 MHz, CDCl_3_): δ 170.6 (CON), 167.3 (CCH_3_), 157.8 (CO_2_tBu), 153.8 (CCH_3_), 137.8 (Car), 129.0 (CHar), 125.33 (CHar), 118.5 (CHar), 107.2 (C_4isoxazole_), 77.3 (C(CH_3_)_3_), 63.9 (CNHBoc), 28.1 (C(CH_3_)_3_), 14.3 (CH_3_), 12.9 (CH_3_), 11.8 (CH_3_) ppm. IR (ATR): 3270, 2982, 2931, 2249, 1705, 1596, 1497, 1362, 1253, 1158, 1063, 1023, 906, 756, 727, 691, 643 cm^−1^. HRMS (ESI-QTOF) m/z: [M+Na]^+^ Calcd. For C_20_H_24_N_4_NaO_4_ 407.1690; Found 407.1693. Chiral HPLC analysis: Chiralpak AD-H column, hexane/*i*-PrOH 90:10, 1 mL/min, λ = 254 nm, major enantiomer (*S*) *t*_R_ = 10.6 min, minor enantiomer (*R*) *t*_R_ = 19.5 min. (*er* 83:17).

*tert*-Butyl (*S*)-(4-(3,5-dimethylisoxazol-4-yl)-5-oxo-1,3-diphenyl-4,5-dihydro-1*H*-pyrazol-4-yl)carbamate (**7e**). Product **7e** was obtained from **3e** according to general procedure. Chromatography on a silica gel using hexane/EtOAc = 3:1 as eluent afforded compound **7e** as a colorless solid (36 mg, 0.080 mmol, 80% yield). [α]_D_^25^ = −151.4 (*c =* 0.5, CHCl_3_). ^1^H NMR (500 MHz, CDCl_3_): δ 7.98 (dd, J = 8.7, 1.3 Hz, 2H, Har), 7.83 (m, 2H, Har), 7.43 (m, 4H, Har), 7.34 (br s, 2H, Har and NH), 7.22 (tt, J = 7.4, 1.2 Hz, 1H, Har), 2.38 (s, 6H, CH_3_), 1.20 (s, 9H, C(CH_3_)_3_) ppm. ^13^C NMR (100 MHz, CDCl_3_): δ 170.7 (CON), 167.8 (CCH_3_), 158.2 (CO_2_tBu), 153.8 (CPh), 138.1 (Car), 131.0 (CHar), 129.0 (CHar), 128.9 (CHar), 126.2 (CHar), 125.4 (CHar), 118.7 (CHar), 107.7 (C_4isoxazole_), 77.2 (C(CH_3_)_3_), 62.7 (CNHBoc), 27.9 (C(CH_3_)_3_), 13.0 (CH_3_), 11.9 (CH_3_) ppm. IR (ATR): 3245, 3128, 2978, 2927, 1731, 1705, 1599, 1490, 1380, 1366, 1256, 1150, 1052, 1026, 906, 756, 735, 687 cm^−1^. HRMS (ESI-QTOF) m/z: [M+Na]^+^ Calcd. For C_25_H_26_N_4_NaO_4_ 469.1846; Found 469.1858. Chiral HPLC analysis: Chiralpak AD-H column, hexane/*i*-PrOH 90:10, 1 mL/min, λ = 254 nm, minor enantiomer (*R*) *t*_R_ = 10.1 min, major enantiomer (*S*) *t*_R_ = 14.6 min. (*er* 93:7).

## 4. Conclusions

In conclusion, we have developed a highly enantioselective Mannich reaction of pyrazolinone ketimines and 1,3-diketones in the presence of a chiral squaramide catalyst derived from quinine. The reaction provides 4-amino-5-pyrazolone derivatives bearing a quaternary substituted stereocenter at C4-position in good yields and enantioselectivities by employing a very low loading of 2 mol% of organocatalyst for a wide range of substrates. Additionally, we achieved the transformation of the adducts obtained in the corresponding enantioenriched 4-pyrazolyl-pyrazolones and 4-isoxazolyl-pyrazolones through condensation with hydrazines and hydroxylamine, opening a new way to the preparation of that kind of compounds.

## Figures and Tables

**Table 1:**
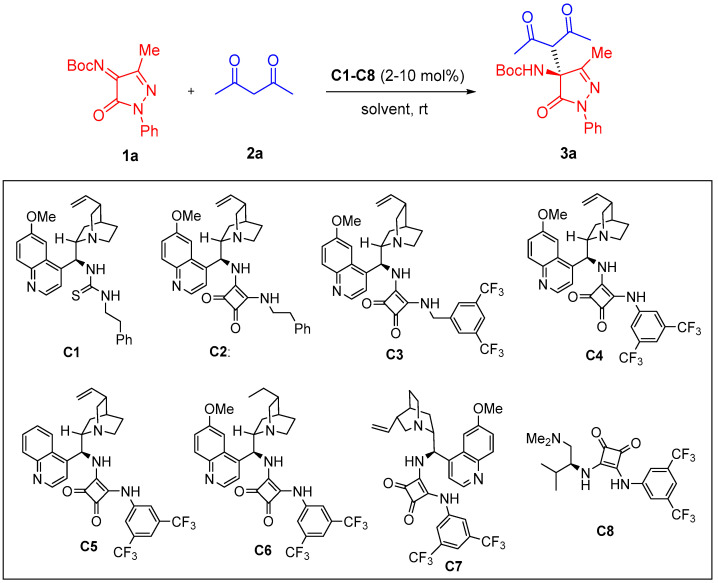
Screening of organocatalysts and optimization of reaction conditions.

Entry	Catalyst (%)	Solvent	t (h)	3a (%) ^b^	er ^c^
1	**C1** (10)	PhMe	4	74	68:32
2	**C2** (10)	PhMe	5	88	80:20
3	**C2** (10)	DCM	1	85	60:40
4	**C2** (10)	CHCl_3_	1	83	78:22
5	**C2** (10)	DCE	1	68	59:41
6	**C2** (10)	Et_2_O	6	90	73:27
7	**C2** (10)	THF	6	82	58:42
8	**C2** (10)	dioxane	3	65	55:45
9	**C2** (10)	EtOAc	2	85	60:40
10	**C2** (10)	MeCN	2	73	20:80
11	**C3** (10)	PhMe	1	80	78:22
12	**C4** (10)	PhMe	1	84	84:16
13	**C5** (10)	PhMe	1.5	68	84:16
14	**C6** (10)	PhMe	2.5	80	80:20
15	**C7** (10)	PhMe	1	76	29:71
16	**C8** (10)	PhMe	4	82	64:36
17	**C4** (5)	PhMe	2	86	84:16
18	**C4** (2)	PhMe	2	92	85:15
19 ^d^	**C4** (2)	PhMe	6	71	84:16
20 ^e^	**C4** (2)	PhMe	2.5	90	85:15

^a^ Reactions were carried out by using **1a** (0.1 mmol), **2a** (0.2 mmol, 2 equiv), and catalyst (10 mol%) in 1.0 mL of solvent at room temperature. ^b^ Yields correspond to isolated compound after flash chromatography. ^c^ Determined by HPLC on a chiral column. ^d^ Reaction at −18 °C. ^e^ Reaction performed with 1.1 equiv of 2,4-pentanedione.

## Data Availability

Not applicable.

## Supplementary Materials

The following supporting information can be downloaded at: https://www.mdpi.com/article/10.3390/molecules27206983/s1, Experimental procedures for the preparation of bifunctional squaramides **C2** and **C8**, NMR Spectra for new compounds and HPLC chromatograms.
