# Supplementary material for: Squaramide-Catalyzed Asymmetric Mannich Reaction between 1,3-Dicarbonyl Compounds and Pyrazolinone Ketimines: A Pathway to Enantioenriched 4-Pyrazolyl- and 4-Isoxazolyl-4-aminopyrazolone Derivatives
Source: Molecules. 2022 Oct 17;27(20):6983. doi: 10.3390/molecules27206983 (PMC9657449; doi:10.3390/molecules27206983)
Supplement: Supplementary file 1 [file molecules-27-06983-s001.zip › Supplementary Material revised.pdf]

Supplementary Material

Squaramide-catalyzed asymmetric Mannich reaction between 1,3-dicarbonyl compounds and pyrazolinone ketimines: a pathway to enantioenriched 4-pyrazolyl- and 4-isoxazolyl-4-aminopyrazolone derivatives.

Marta Gil-Ordóñez,[1] Camille Aubry,[1] Cristopher Niño,[1] Alicia Maestro[1]* and José M. Andrés[1]*

[1]GIR SintACat, Instituto CINQUIMA and Departamento de Química Orgánica, Facultad de Ciencias, Universidad de Valladolid, Paseo de Belén 7, 47011-Valladolid. Spain.

E-mail: alicia.maestro@uva.es; jmandres@uva.es

List of contents

1. Synthesis of bifunctional squaramides **C2** and **C8**...................................... S2

2. NMR Spectra for New Compounds S5

3. HPLC Profiles of the isolated compounds.. S31

1. Synthesis of bifunctional squaramides C2 and C8.

1.1. Preparation of squaramide organocatalyst C2.

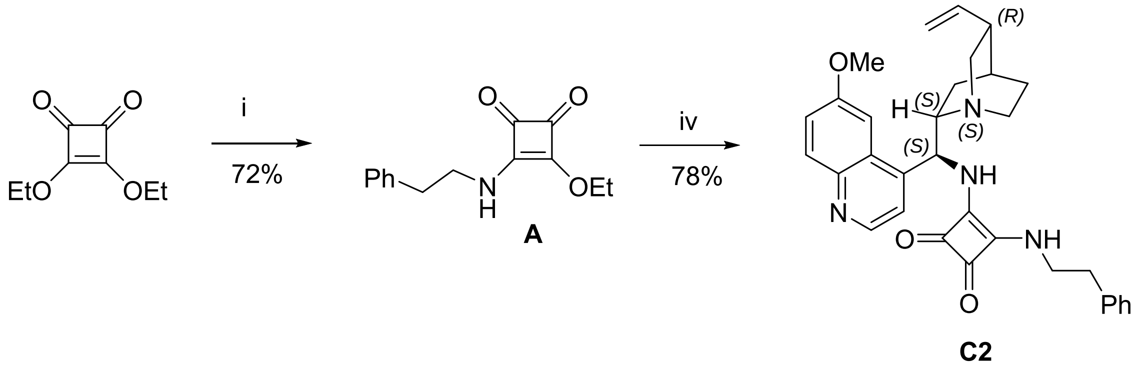

3-Ethoxy-4-(phenethylamino)cyclobut-3-ene-1,2-dione (A).

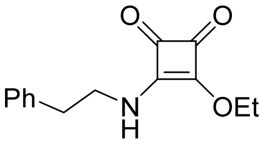

To a solution of diethyl squarate (955 mg, 5.60 mmol) in CH_2Cl_2 (6 mL) was added a 2-phenylethan-1-amine (712 mg, 5.88 mmol, 1.05 equiv) and the mixture was stirred at room temperature for 48 h. The reaction mixture was concentrated and purified by column chromatography on silica gel (Hexane/EtOAc = 1:1) to afford the desired product as a white solid: 989 mg (4.03 mmol, 72% yield). Mp 104-105 ºC. **^{1}H-NMR** (500 MHz, $CDCl_3$) δ 7.31 (m, 2H, $\underline{H}$ar), 7.25 (m, 1H, $\underline{H}$ar), 7.18 (m, 2H, $\underline{H}$ar), 6.71 (br s, 1H, N$\underline{H}$), 4.73 (q, J = 7.1 Hz, 2H, $C\underline{H}_2CH_3$), 3.69 (m, 2H, $C\underline{H}_2CH_2$), 2.92 (t, J = 7.0 Hz, 2H, $C\underline{H}_2CH_2$), 1.45 (t, J = 7.2 Hz, 3H, $CH_2C\underline{H}_3$). **^{13}C-NMR** (101 MHz, $CDCl_3$): δ 189.6 ($\underline{C}$O), 182.6 ($\underline{C}$O), 177.6 ($\underline{C}$=C), 172.3 (C=$\underline{C}$), 137.4 ($\underline{C}$ar), 128.9 ($\underline{C}$Har), 128.7 ($\underline{C}$Har), 126.9 ($\underline{C}$Har), 69.7 ($\underline{C}H_2CH_3$), 46.1 ($\underline{C}H_2N$), 37.0 ($\underline{C}H_2Ph$), 15.8 ($\underline{C}H_3$) ppm**. IR** (ATR): 3255, 3044, 3024, 2937, 1807, 1690, 1596, 1522, 1334, 1116, 1059, 1019, 838, 751, 691 cm^{-1}. **HRMS** (ESI-QTOF) m/z: $[M+H]^+$ Calcd. For $C_{14}H_{16}NO_3$ 246.1125; Found 246.1129.

3-(((*S*)-(6-methoxyquinolin-4-yl)((1*S*,2*S*,4*S*,5*R*)-5-vinylquinuclidin-2-yl)methyl)amino)-4-(phenethylamino)cyclobut-3-ene-1,2-dione (C2).

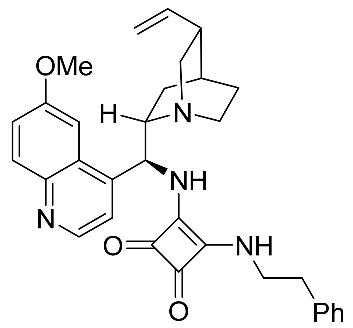

C2

To a stirred solution of 9-amino-(9-deoxy)epiquinine (162 mg, 0.5 mmol) in methanol (6 mL) was added 3-ethoxy-4-(phenethylamino)cyclobut-3-ene-1,2-dione (**1**) (123 g, 0.5 mmol). After stirring for 48 h, a white precipitate formed, which was filtered and washed with methanol (3 x 5 mL) yielding the squaramide **IV** (204 mg, 0.39 mmol, 78%). Colorless solid. Mp 279-280 ºC. $[\alpha]_D^{25}$ = +4.52 (*c* = 0.4, DMSO). **^{1}H NMR** (500 MHz, DMSO-d$_6$) δ 8.77 (d, J = 4.6 Hz, 1H, NH), 7.95 (d, J = 9.2 Hz, 1H, Har), 7.83 (br s, 1H, Har), 7.77 (br s, 1H, Har), 7.55 (br s, 1H, Har), 7.42 (dd, J = 9.2, 2.6 Hz, 2H, Har), 7.17 (m, 5H, Har), 5.94 (m, 2H, CH=CH$_2$ and NH), 4.98 (m, 2H, CH$_2$=CH), 3.90 (s, 3H, CH$_3$O), 3.70 (m, 2H, CH$_2$NH), 3.37 (m, 1H), 3.28 (m, 1H), 3.16 (dd, J = 13.6, 10.0 Hz, 1H), 2.78 (br s, 2H), 2.61 (m, 2H), 2.25 (br s, 1H), 1.54 (br s, 1H), 1.47 (br s, 2H), 0.56 (br s, 1H) ppm. **^{13}C-NMR** (101 MHz, DMSO-d$_6$): δ 182.8 (CO), 182.4 (CO), 168.1 (C=C), 167.0 (C=C), 158.3 (Car), 148.2 (CHar), 144.7 (Car), 144.1 (Car), 142.7 (CHar), 138.8 (Car), 131.9 (CHar), 129.1 (CHar), 128.8 (CHar), 126.7 (CHar), 122.3 (CHar), 114.7 (CH$_2$=CH), 102.1 (CHar), 59.1 (CHN), 56.2 (CH$_2$N), 56.1 (CH$_3$O), 45.0 (CH$_2$N), 40.5 (CH$_2$) 39.8 (CHCH=CH$_2$), 37.3 (CH$_2$Ph), 27.8 (CH$_2$), 26.7 (CH$_2$) ppm**.** **IR** (ATR): 3159, 2935, 1800, 1638, 1567, 1360, 1240, 1033, 840, 747, 700 cm^{-1}. **HRMS** (ESI-QTOF) m/z: $[M+H]^+$ Calcd. For $C_{32}H_{35}N_4O_3$ 523.2704; Found 523.2708.

1.2. Preparation of squaramide organocatalyst C8.

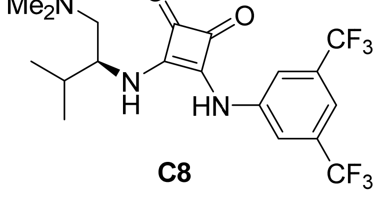

C8

To a stirred solution of (S)-N^1,N^1,3-trimethylbutane-1,2-diamine[1] (169 mg, 1.3 mmol) in CH_2Cl_2 (5 mL) was added 3-((3,5-bis(trifluoromethyl)phenyl)amino)-4-ethoxycyclobutane-1,2-dione (459 mg, 1.3 mmol). After stirring for 48 h, a precipitate was formed, which was filtered and washed with diethyl ether, yielding the catalyst **C8** as a white solid (420 mg, 0.96 mmol, 74% yield). Mp 208-210 ºC. $[\alpha]_D^{25}$ = -11.6 (c = 0.4, acetone). **^{1}H-NMR** (400 MHz, DMSO-d_6) δ 8.06 (s, 2H, H̲ar), 7.78 (br s, 1H, NH), 7.62 (s, 1H, H̲ar), 4.09 (br s, 1H, CH̲N), 2.46 (m, 1H, CHH̲NMe$_2$), 2.28 (m, 1H, CH̲HNMe$_2$), 2.15 (s, 6H, CH̲$_3$N), 1.84 (m, 1H, CH̲Me$_2$), 0.89 (d, J = 6.5 Hz, 3H, CH̲$_3$CH), 0.87 (d, J = 6.5 Hz, 3H, CH̲$_3$CH) ppm. **^{13}C-NMR** (101 MHz, DMSO-d_6): δ 185.0 (C̲O), 180.6 (C̲O), 170.5 (C̲=C), 162.6 (C=C̲), 141.6 (C̲ar), 131.8 (q, J = 32.9 Hz, C̲CF$_3$), 123.6 (q, J = 272.8 Hz, C̲F$_3$), 118.5 (C̲Har), 115.1 (C̲Har), 61.7 (C̲H$_2$NMe$_2$), 57.7 (C̲HN), 45.7 (C̲H$_3$N), 31.1 (C̲HMe$_2$), 19.7 (C̲H$_3$CH), 17.1 (C̲H$_3$CH) ppm. **^{19}F-NMR** (376 MHz, $CDCl_3$): δ -61.8 ppm. **IR** (ATR): 3135, 2948, 1798, 1662, 1578, 1542, 1457, 1377, 1274, 1124, 940, 882, 743, 699, 685 cm^{-1}. **HRMS** (ESI-QTOF) m/z: $[M+H]^+$ Calcd. For $C_{19}H_{22}F_6N_3O_2$ 438,1611; Found 438,1614.

[1] J. M. Andrés, R. Manzano, R. Pedrosa. *Chem. Eur. J.* **2008**, *14*, 5116.

2. NMR Spectra for New Compounds

3-Ethoxy-4-(phenethylamino)cyclobut-3-ene-1,2-dione (A).

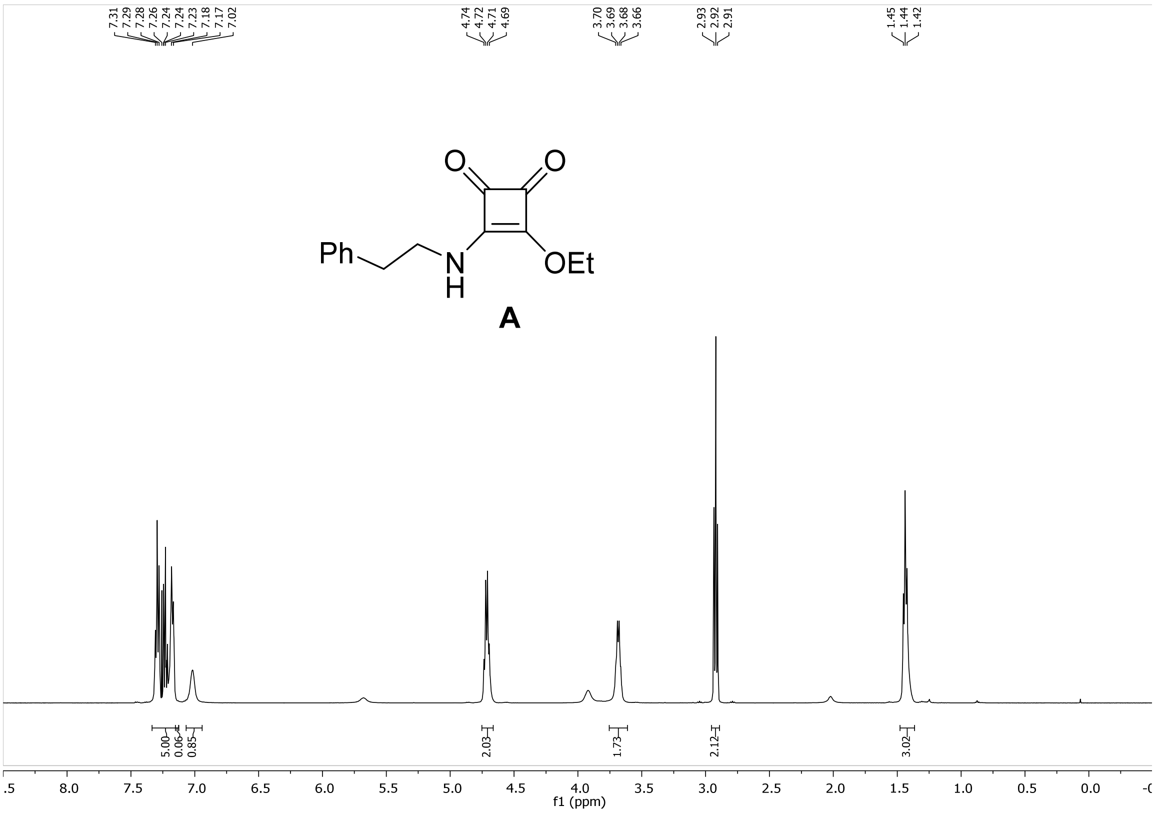

Figure S1. ^{1}H NMR spectrum of **A** ($CDCl_3$, 500 MHz).

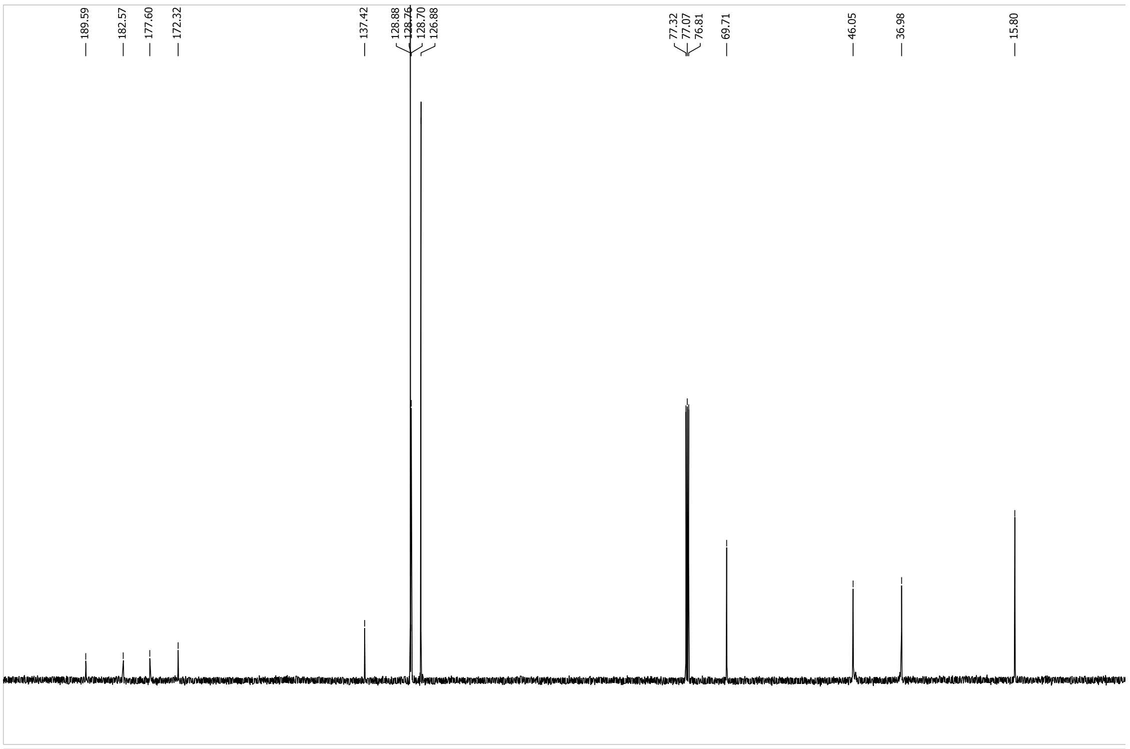

Figure S2. ^{13}C NMR spectrum of **A** ($CDCl_3$, 101 MHz).

3-(((*S*)-(6-Methoxyquinolin-4-yl)((1*S*,2*S*,4*S*,5*R*)-5-vinylquinuclidin-2-yl)methyl)amino)-4-(phenethylamino)cyclobut-3-ene-1,2-dione (C2).

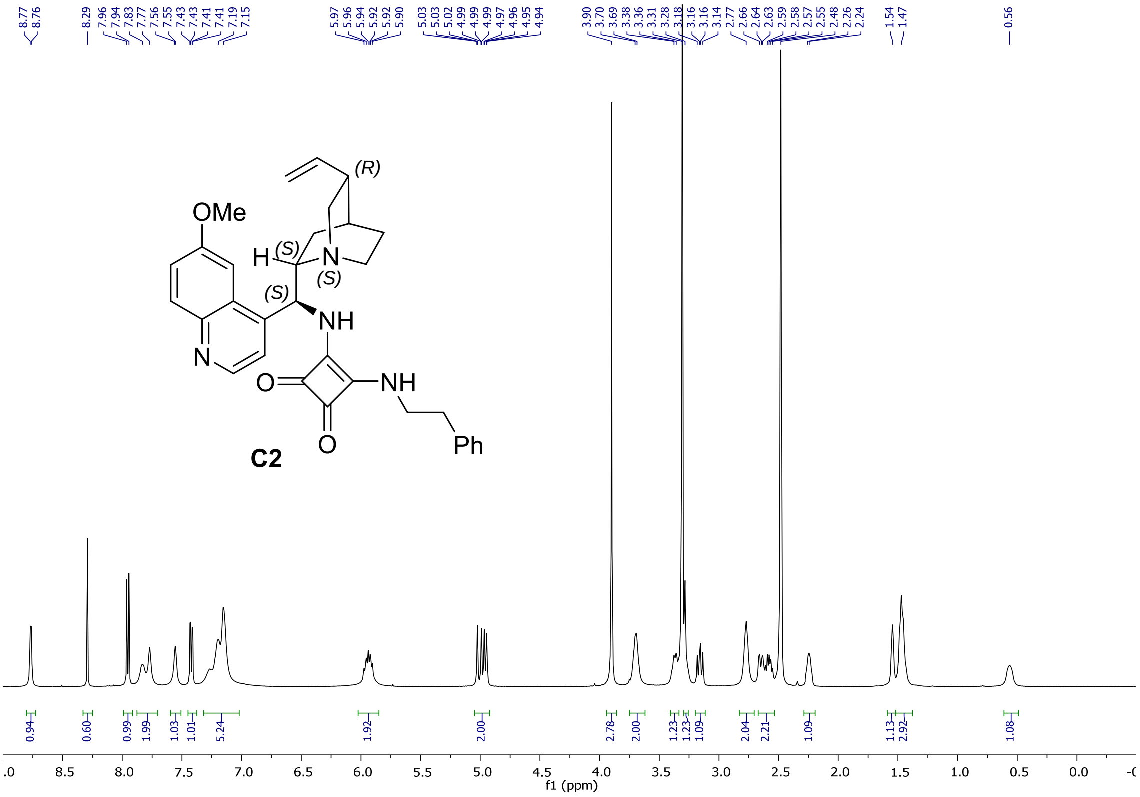

Figure S3. ^{1}H NMR spectrum of **C2** (DMSO-d6, 500 MHz).

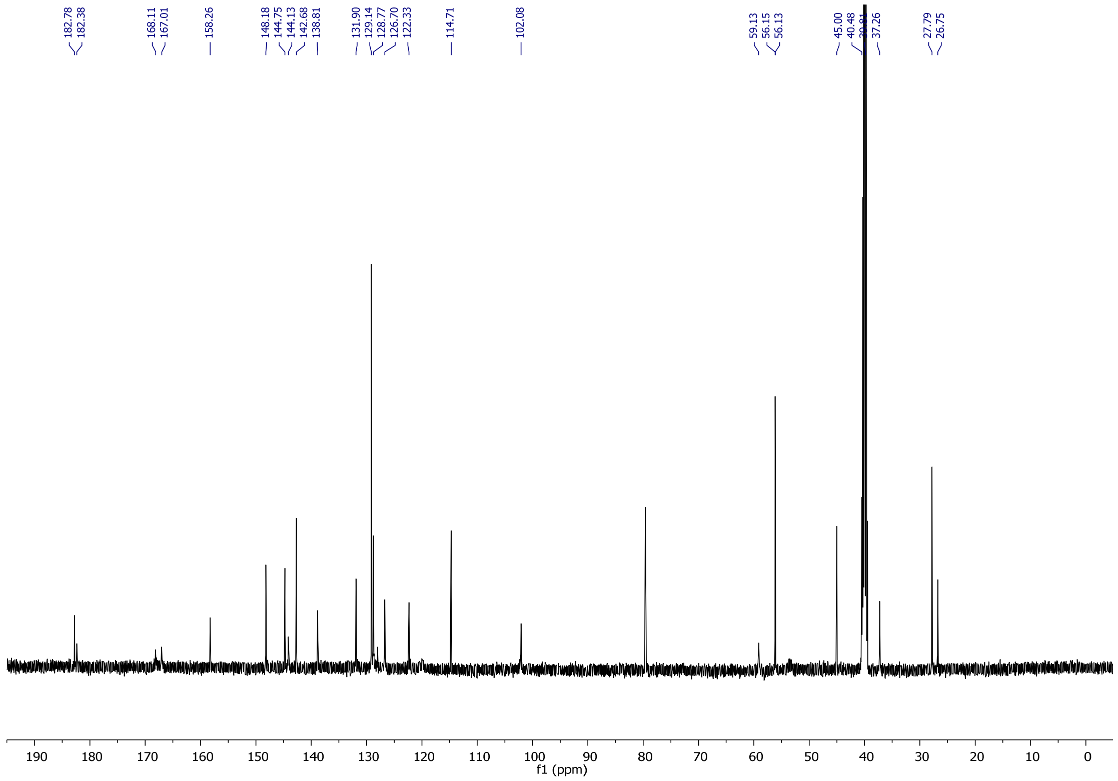

Figure S4. ^{13}C NMR spectrum of **C2** (DMSO-d_6, 101 MHz).

(*S*)-3-((3,5-Bis(trifluoromethyl)phenyl)amino)-4-((1-(dimethylamino)-3-methylbutan-2-yl)amino)cyclobut-3-ene-1,2-dione (C8).

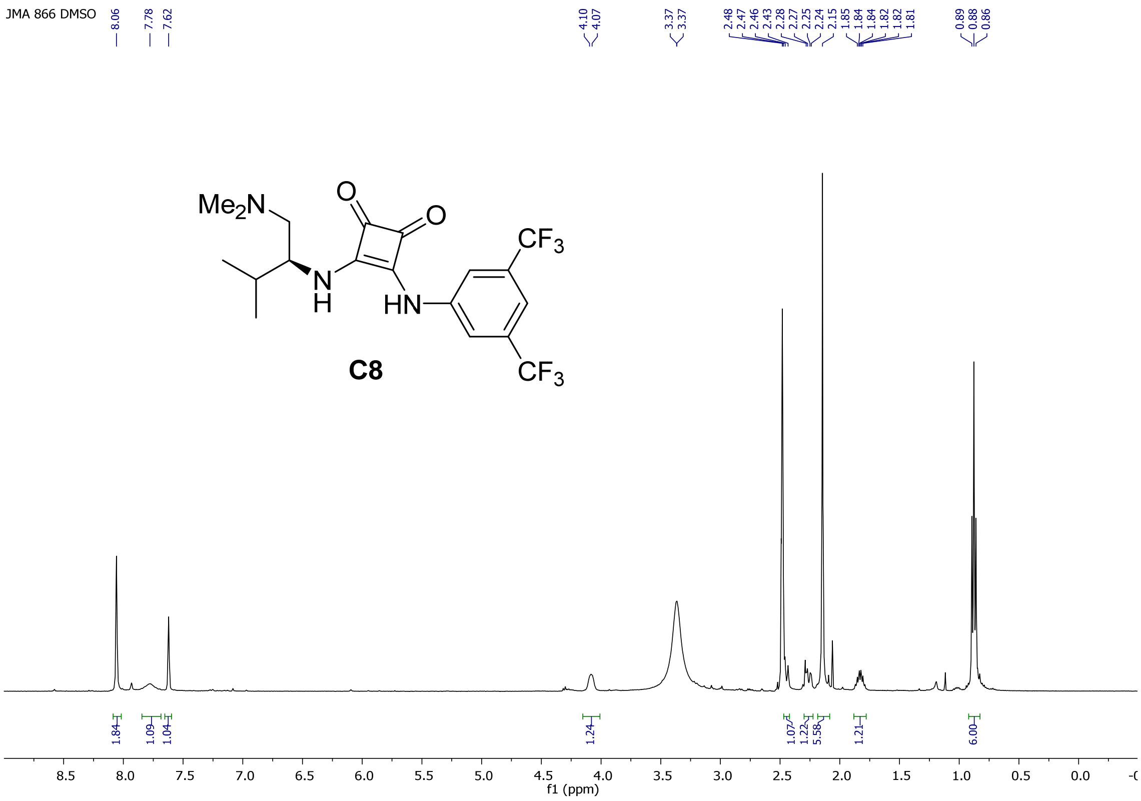

Figure S5. ^{1}H NMR spectrum of **C8** (DMSO-d6, 400 MHz).

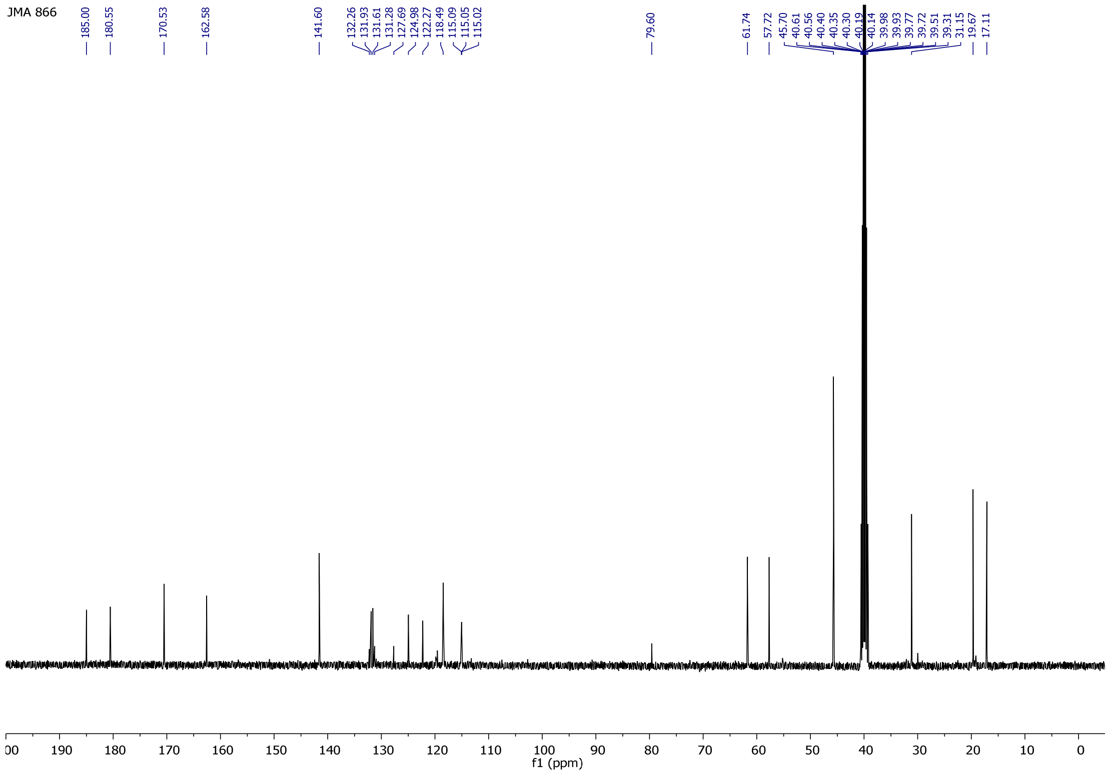

Figure S6. ^{13}C NMR spectrum of **C8** (DMSO-d_6, 101 MHz).

***tert*-Butyl (*S*)-(4-(2,4-dioxopentan-3-yl)-3-methyl-5-oxo-1-phenyl-4,5-dihydro-1*H*-pyrazol-4-yl)carbamate (3a).**

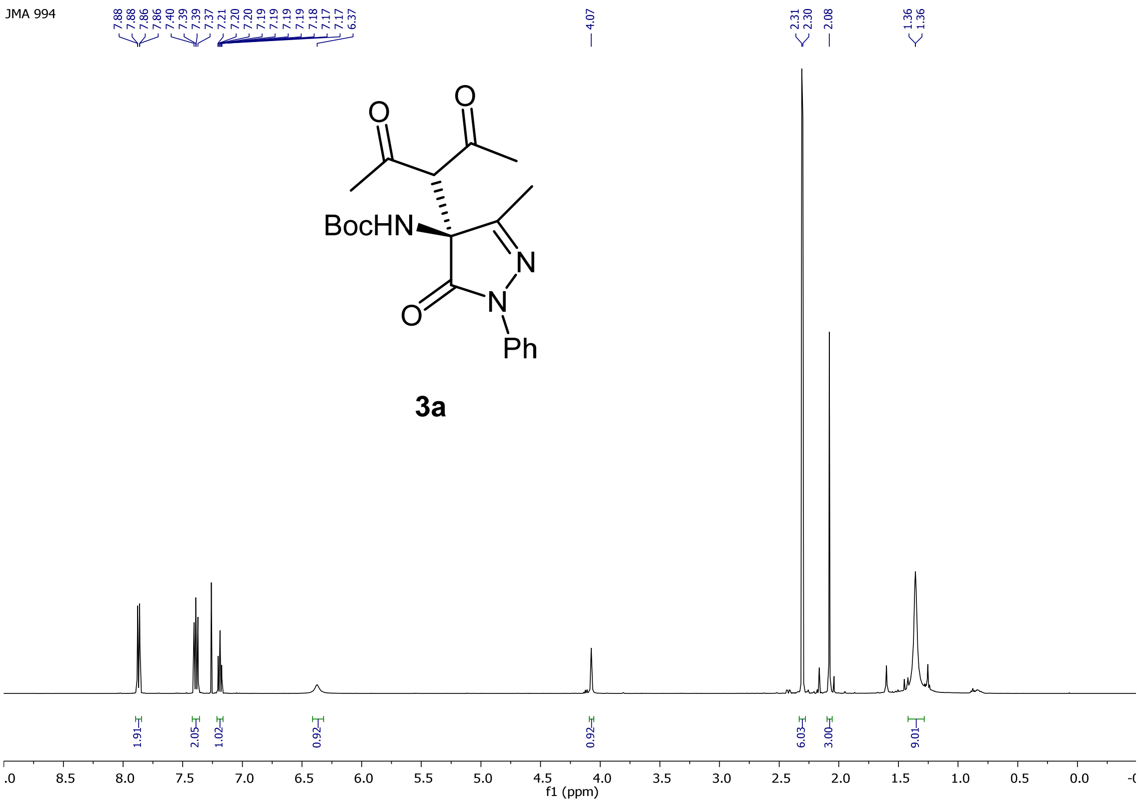

Figure S7. ^{1}H NMR spectrum of **3a** ($CDCl_3$, 500 MHz).

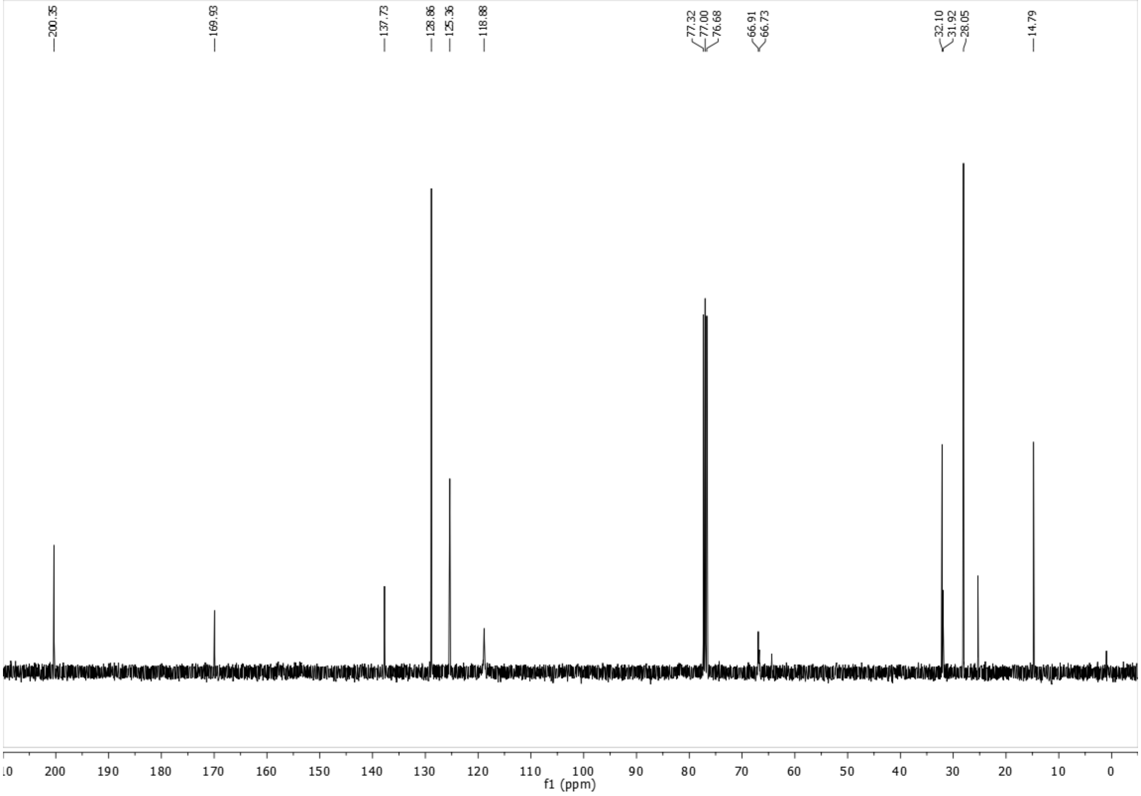

Figure S8. ^{13}C NMR spectrum of **3a** ($CDCl_3$, 126 MHz).

***tert*-Butyl (*S*)-(4-(2,4-dioxopentan-3-yl)-3-ethyl-5-oxo-1-phenyl-4,5-dihydro-1*H*-pyrazol-4-yl)carbamate (3b).**

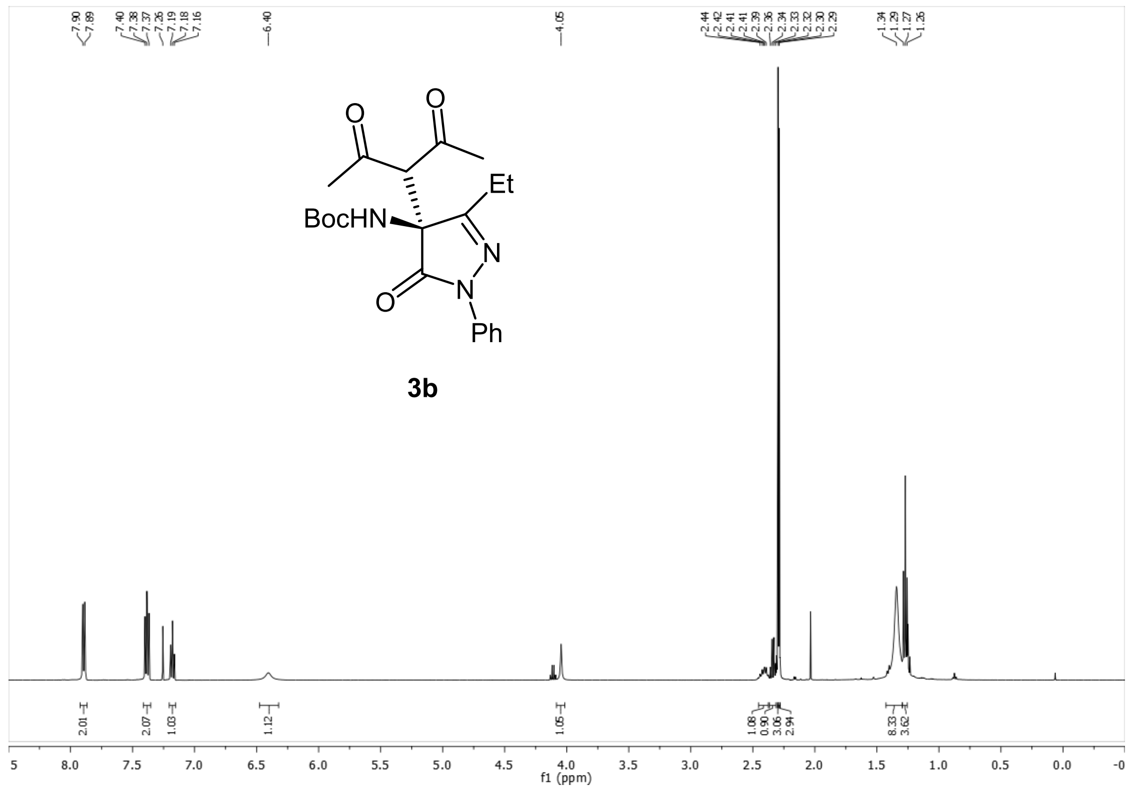

Figure S9. ^{1}H NMR spectrum of **3b** ($CDCl_3$, 500 MHz).

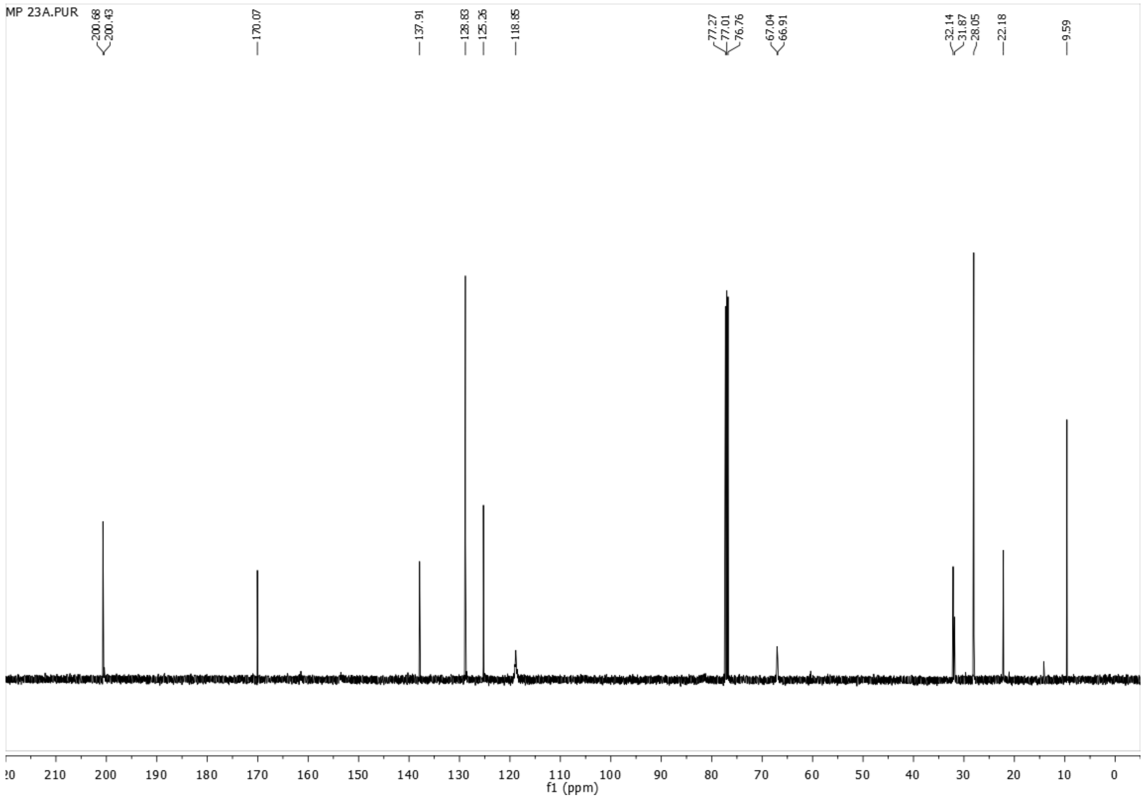

Figure S10. ^{13}C NMR spectrum of **3b** ($CDCl_3$, 126 MHz).

***tert*-Butyl (*S*)-(4-(2,4-dioxopentan-3-yl)-3-isopropyl-5-oxo-1-phenyl-4,5-dihydro-1*H*-pyrazol-4-yl)carbamate (3c).**

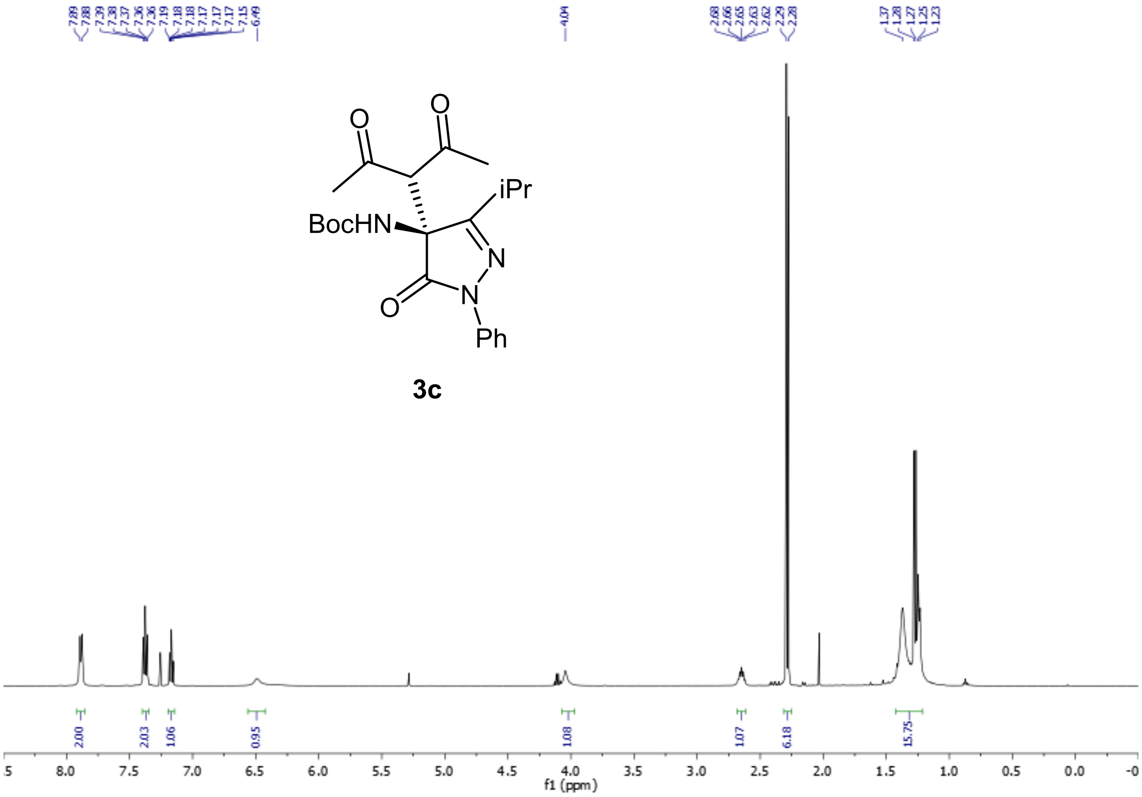

Figure S11. ^{1}H NMR spectrum of **3c** ($CDCl_3$, 500 MHz).

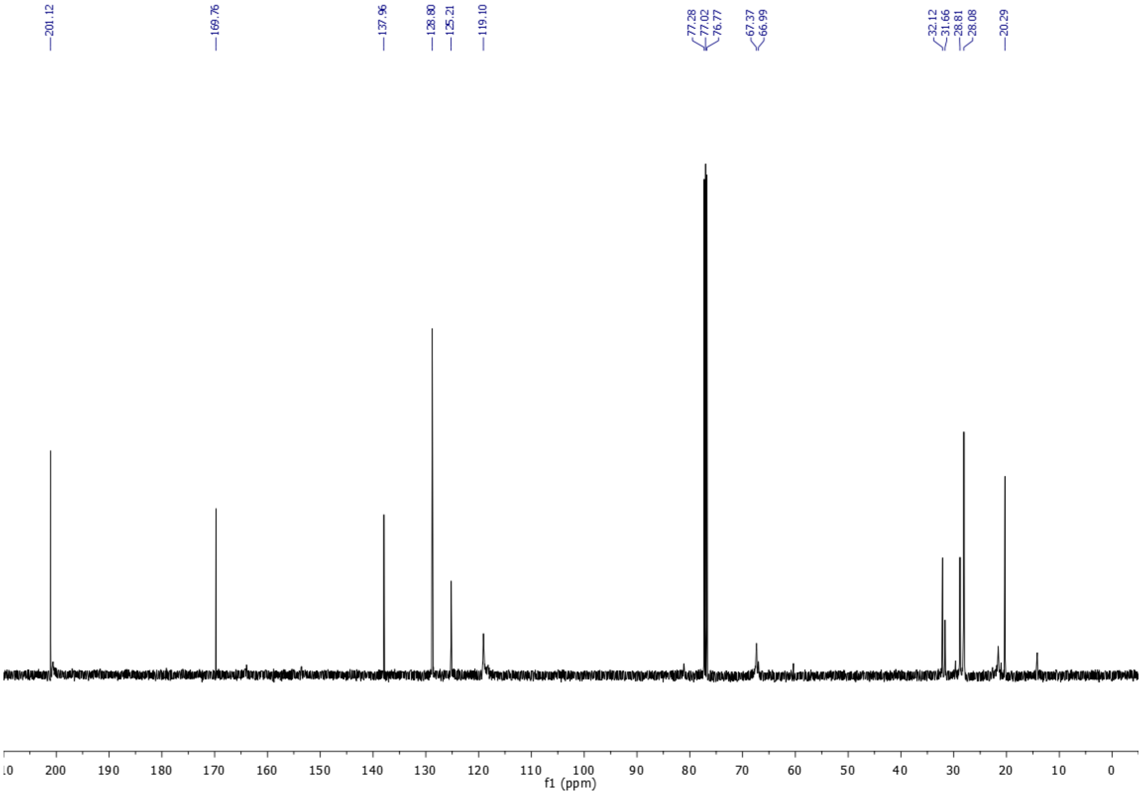

Figure S12. ^{13}C NMR spectrum of **3c** ($CDCl_3$, 126 MHz).

***tert*-Butyl (*S*)-(4-(2,4-dioxopentan-3-yl)-5-oxo-1,3-diphenyl-4,5-dihydro-1*H*-pyrazol-4-yl)carbamate (3e).**

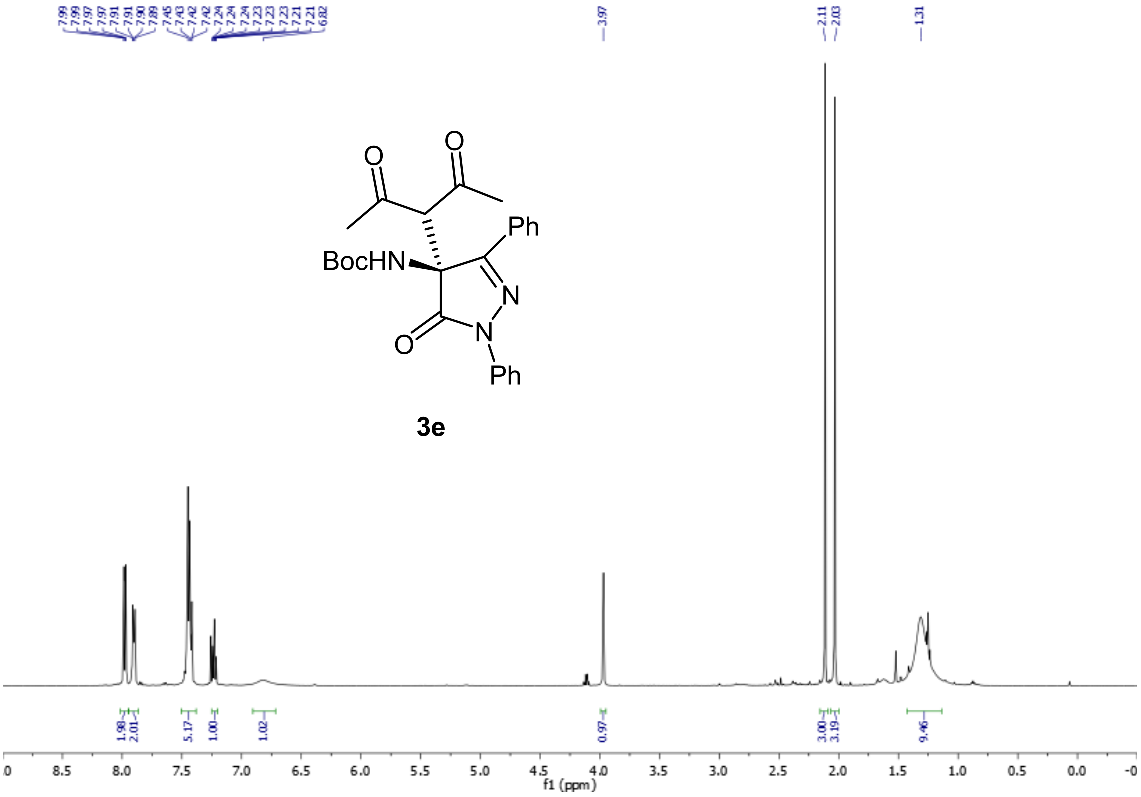

Figure S13. ^{1}H NMR spectrum of **3e** ($CDCl_3$, 500 MHz).

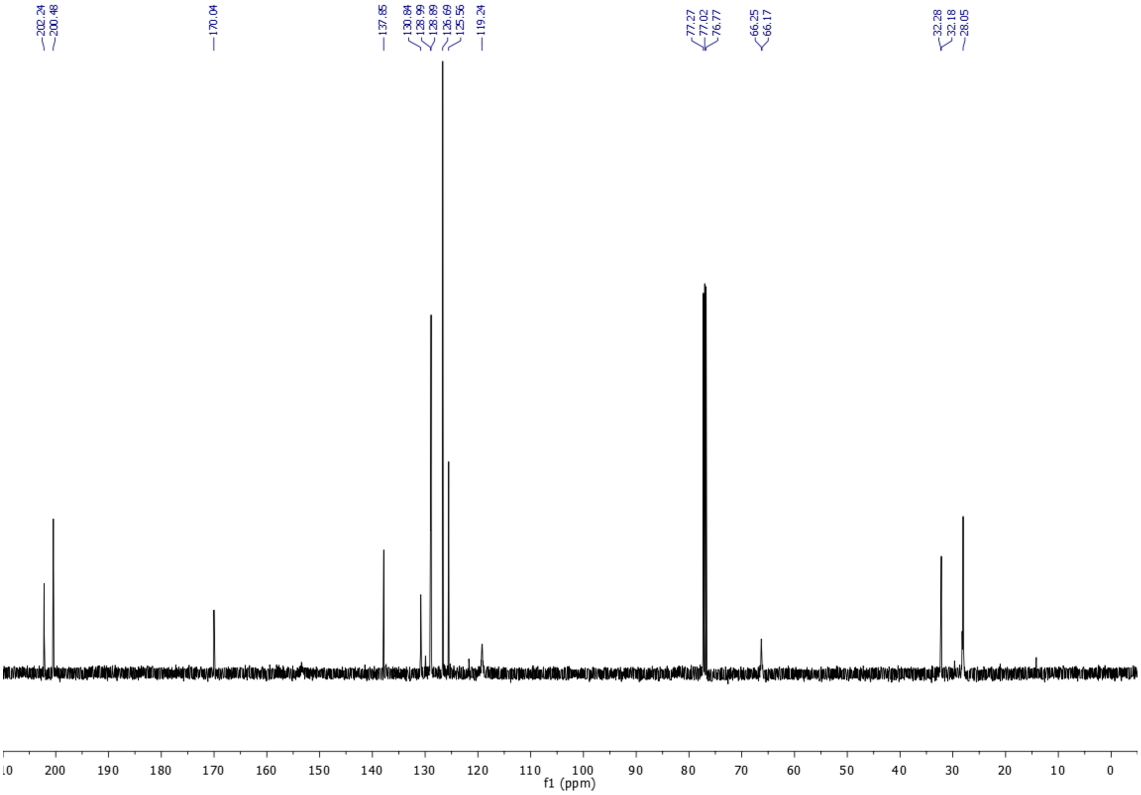

Figure S14. ^{13}C NMR spectrum of **3e** ($CDCl_3$, 126 MHz).

***tert*-Butyl (*S*)-(1-(4-chlorophenyl)-4-(2,4-dioxopentan-3-yl)-3-methyl-5-oxo-4,5-dihydro-1*H*-pyrazol-4-yl)carbamate (3f).**

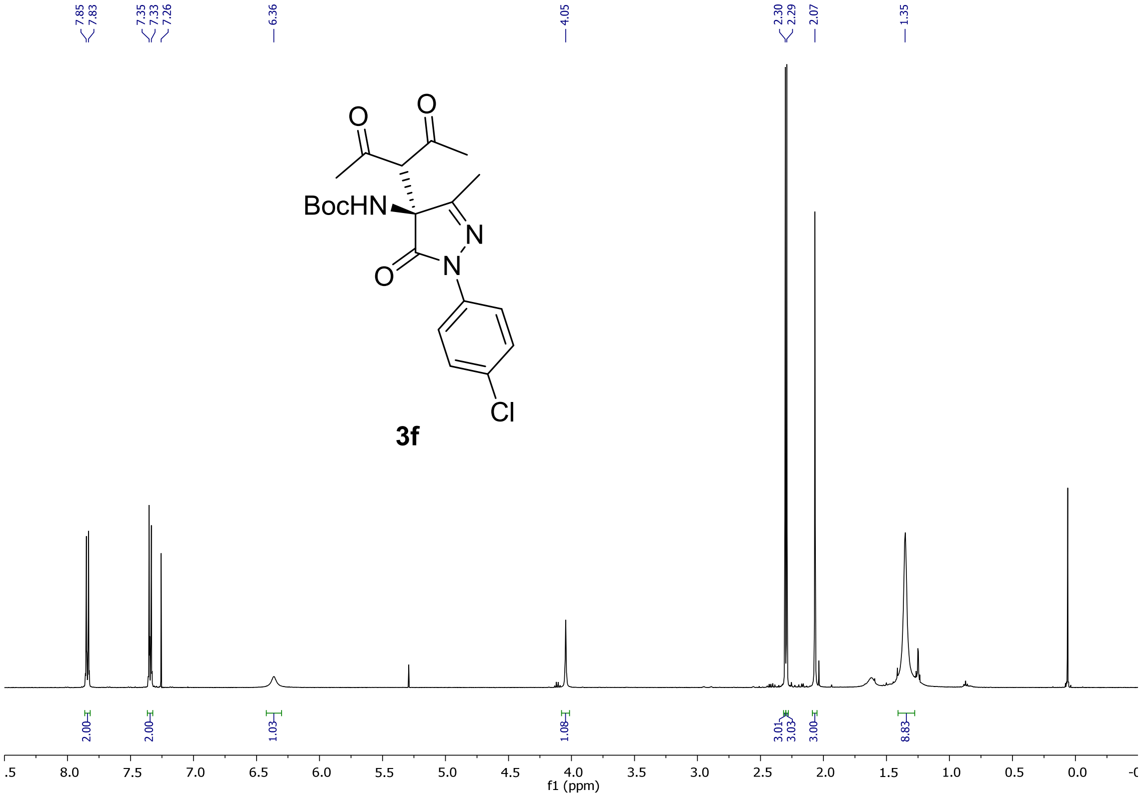

Figure S15. ^{1}H NMR spectrum of **3f** ($CDCl_3$, 500 MHz).

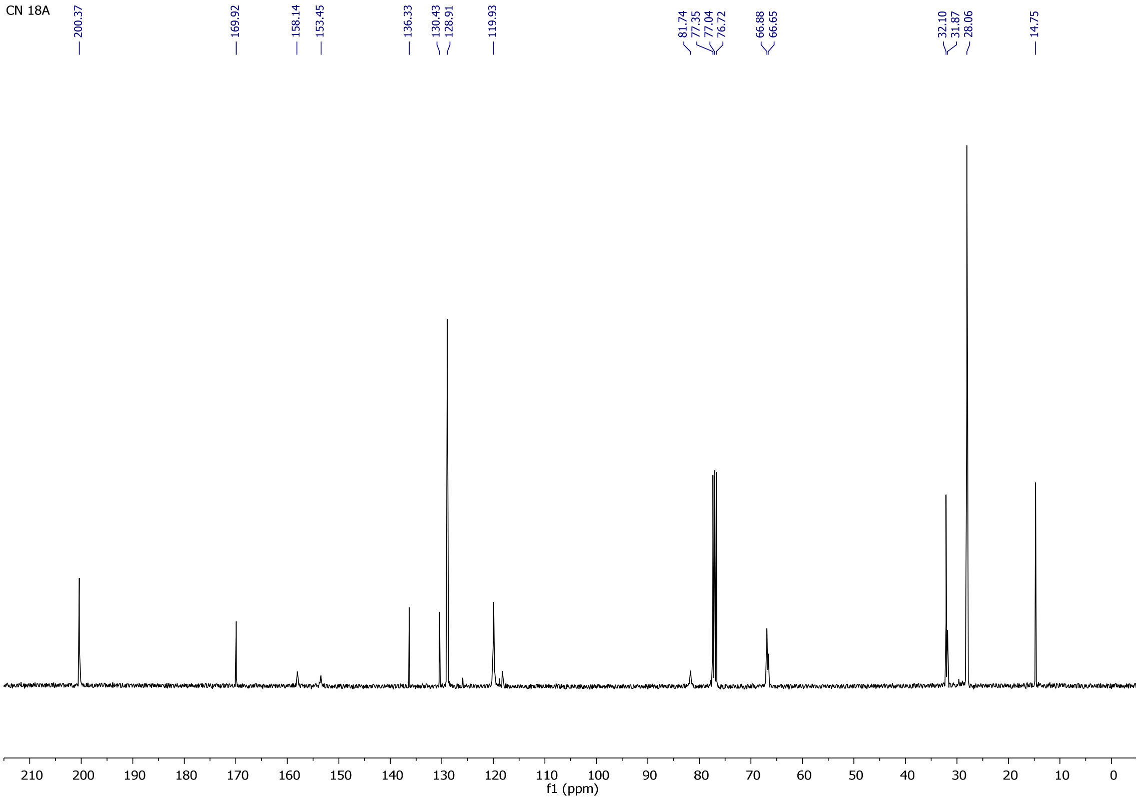

Figure S16. ^{13}C NMR spectrum of **3f** ($CDCl_3$, 126 MHz).

tert-Butyl (S)-(4-(2,4-dioxopentan-3-yl)-3-methyl-5-oxo-1-(p-tolyl)-4,5-dihydro-1H-pyrazol-4-yl)carbamate (3g).

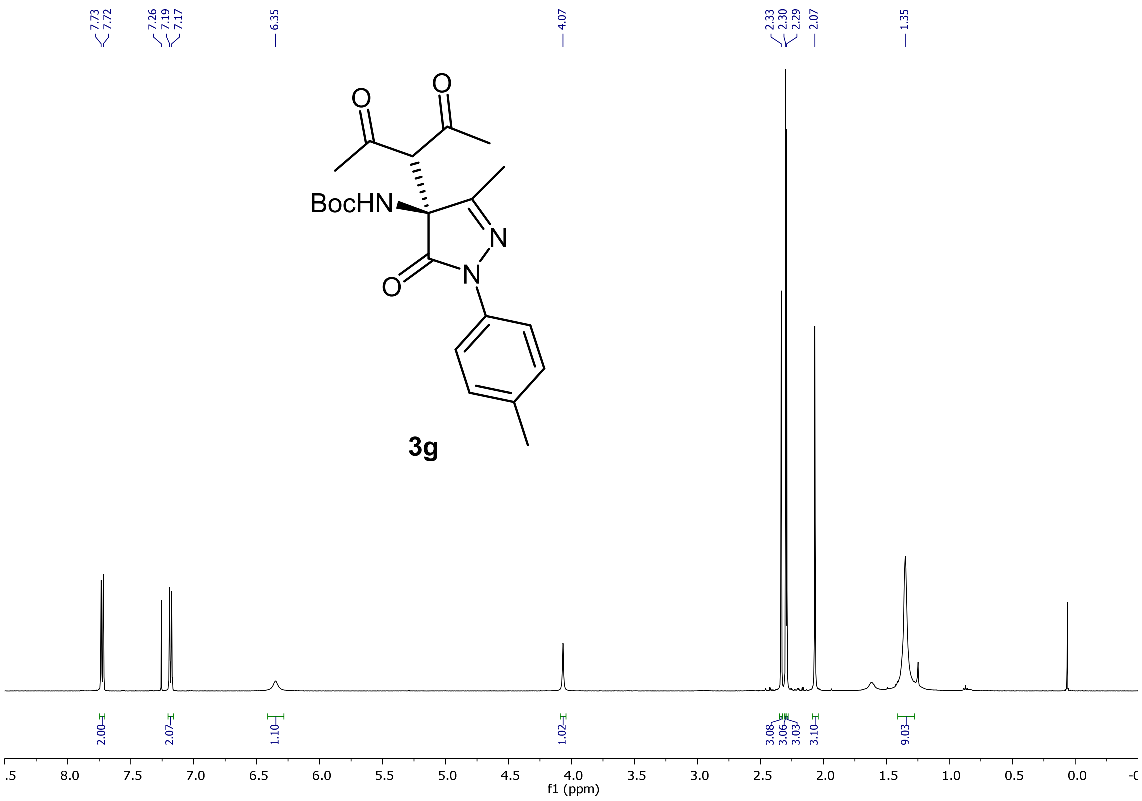

Figure S17. ^{1}H NMR spectrum of **3g** ($CDCl_3$, 500 MHz).

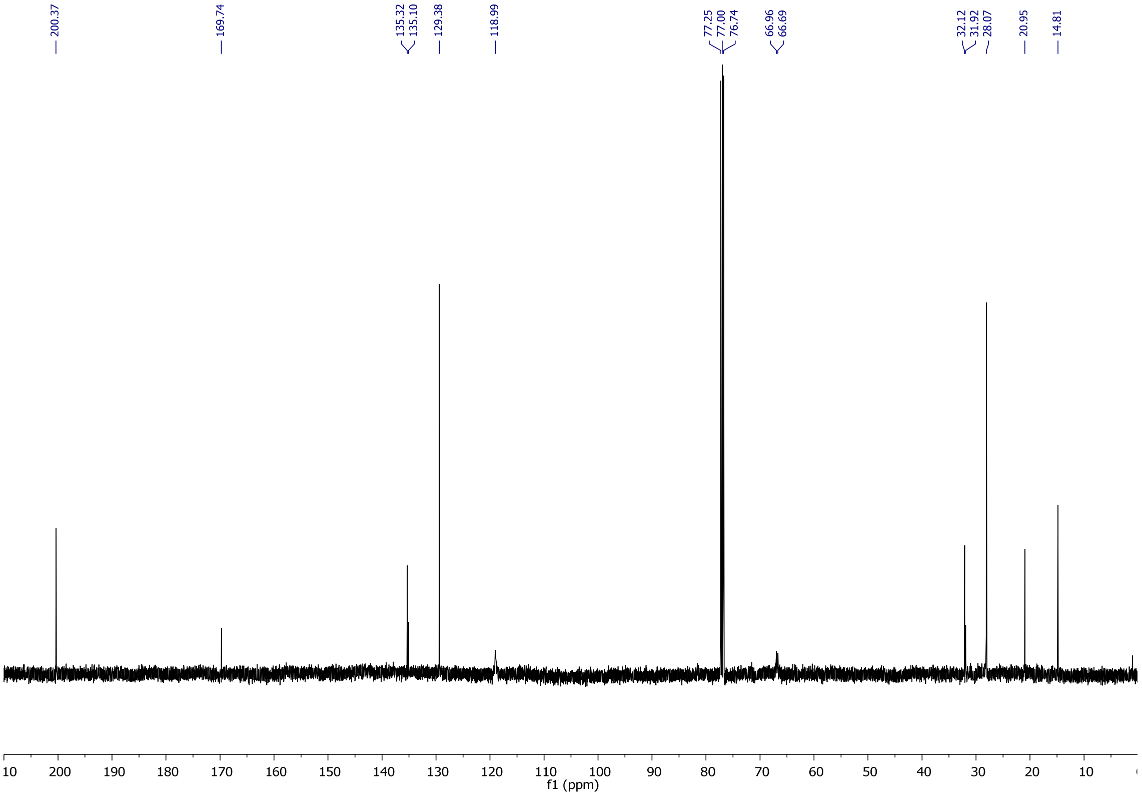

Figure S18. ^{13}C NMR spectrum of **3g** ($CDCl_3$, 126 MHz).

***tert*-Butyl (*S*)-(4-(3,5-dioxoheptan-4-yl)-3-methyl-5-oxo-1-phenyl-4,5-dihydro-1*H*-pyrazol-4-yl)carbamate (3h).**

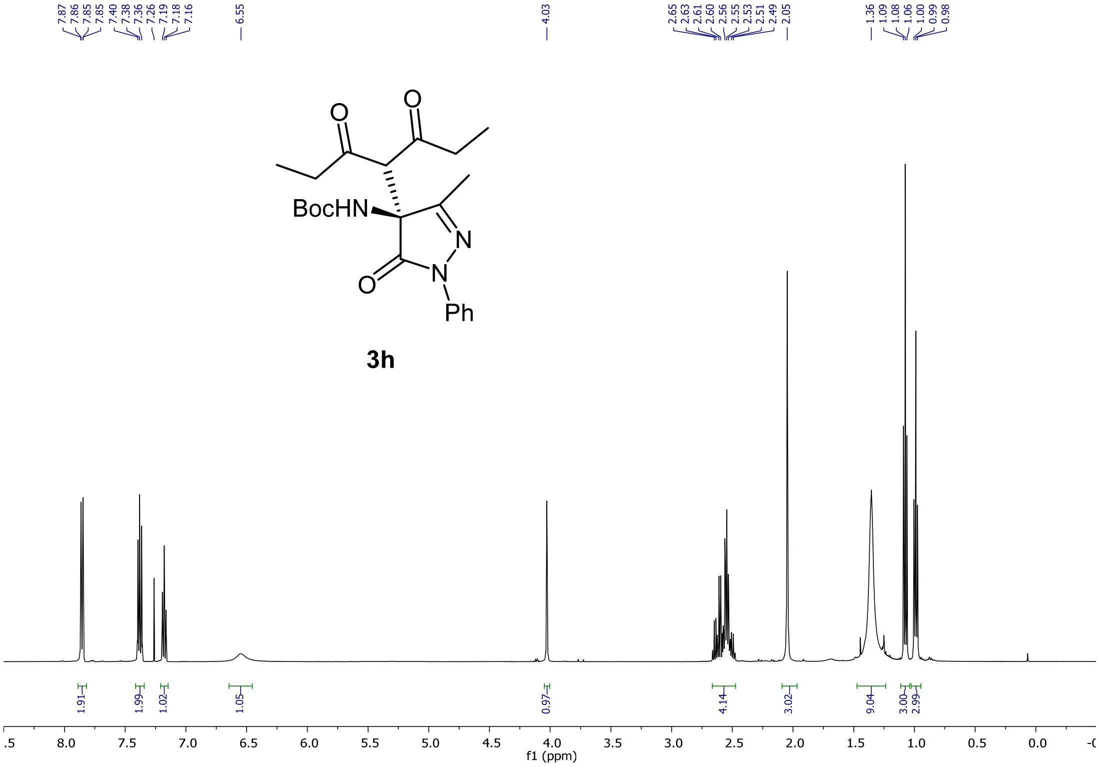

Figure S19. ^{1}H NMR spectrum of **3h** ($CDCl_3$, 500 MHz).

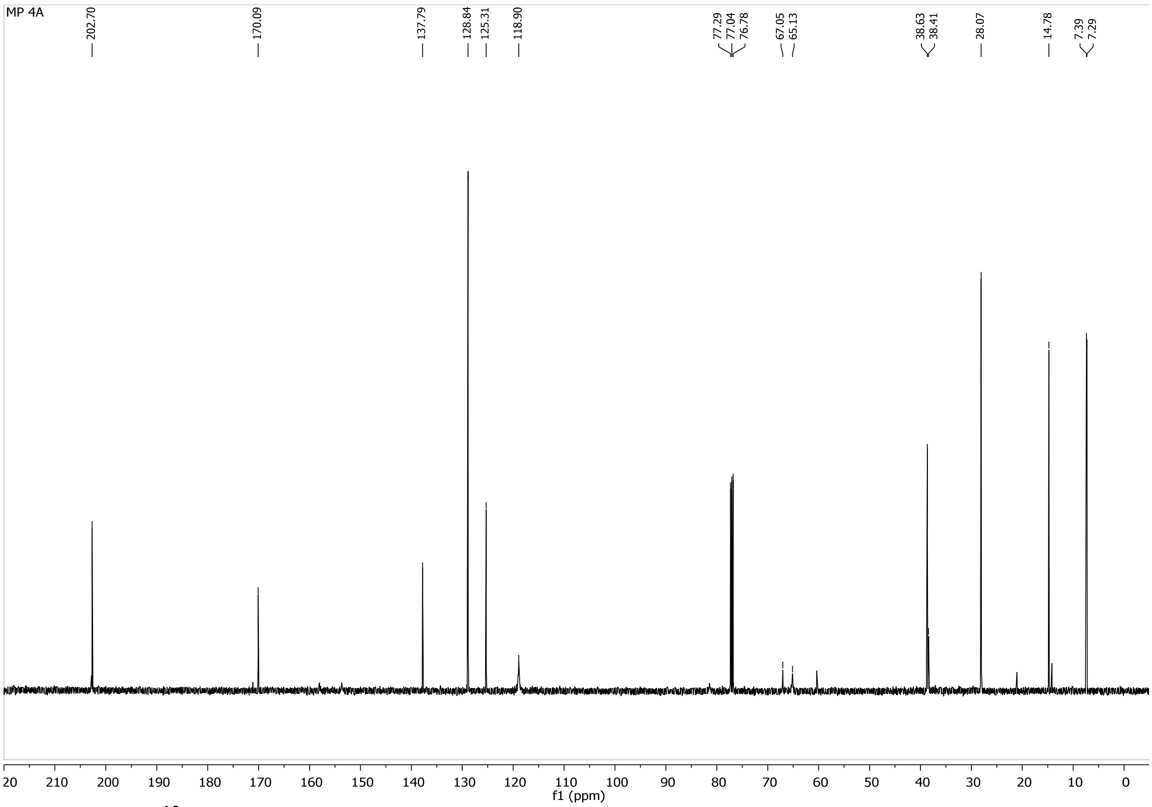

Figure S20. ^{13}C NMR spectrum of **3h** ($CDCl_3$, 126 MHz).

tert-Butyl (S)-(4-(1,3-dioxo-1,3-diphenylpropan-2-yl)-3-methyl-5-oxo-1-phenyl-4,5-dihydro-1H-pyrazol-4-yl)carbamate (3i).

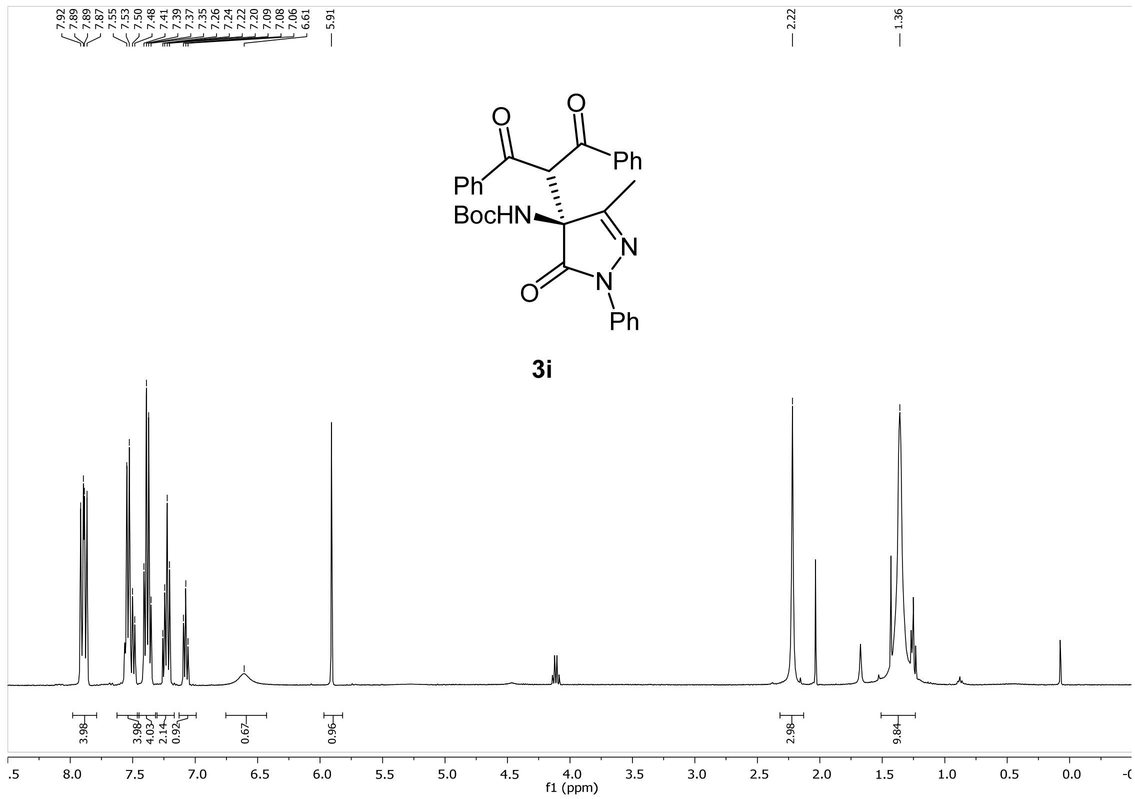

Figure S21. ^{1}H NMR spectrum of **3i** ($CDCl_3$, 500 MHz).

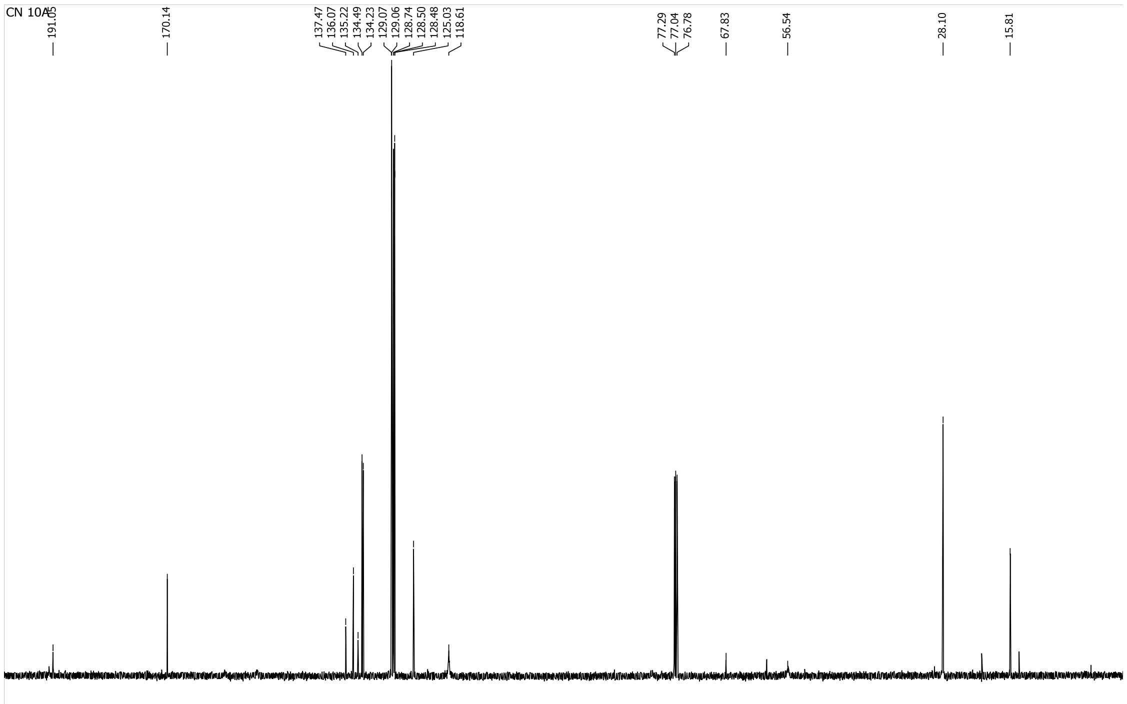

Figure S22. ^{13}C NMR spectrum of **3i** ($CDCl_3$, 126 MHz).

***tert*-Butyl (*S*)-(4-(3,5-dioxoheptan-4-yl)-5-oxo-1,3-diphenyl-4,5-dihydro-1*H*-pyrazol-4-yl)carbamate (3j).**

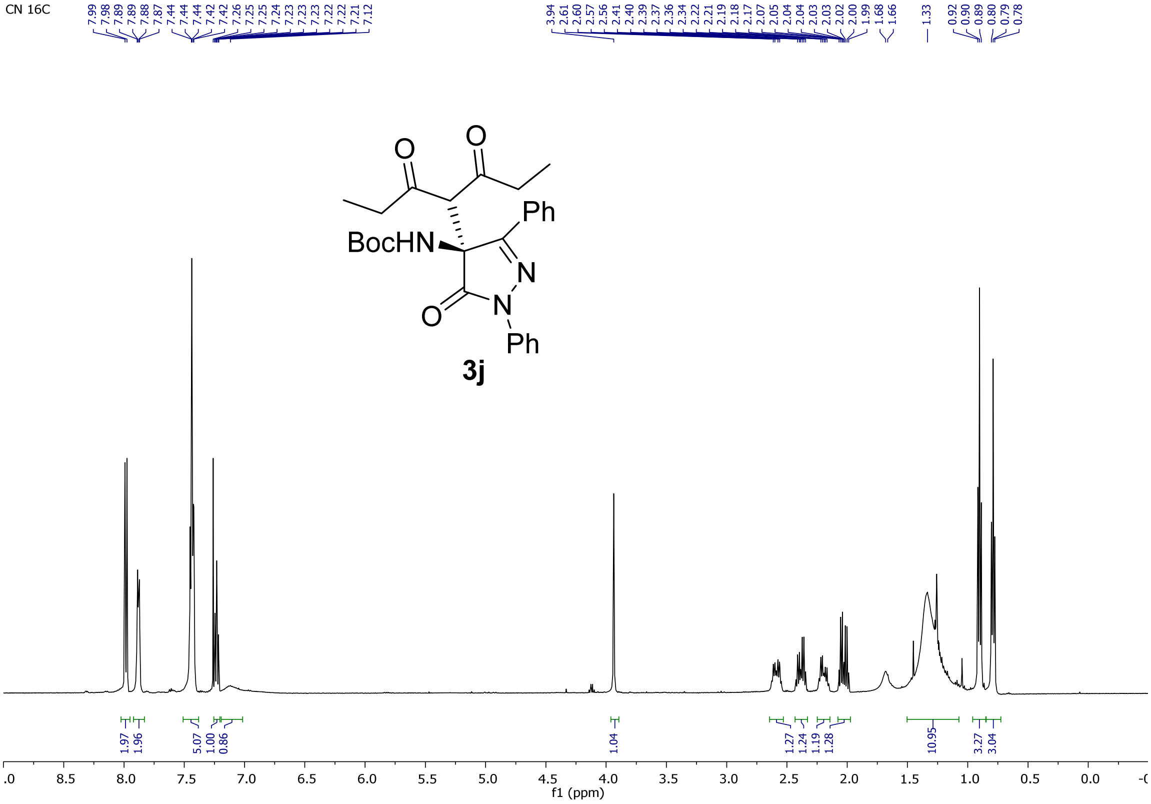

Figure S23. ^{1}H NMR spectrum of **3j** ($CDCl_3$, 500 MHz).

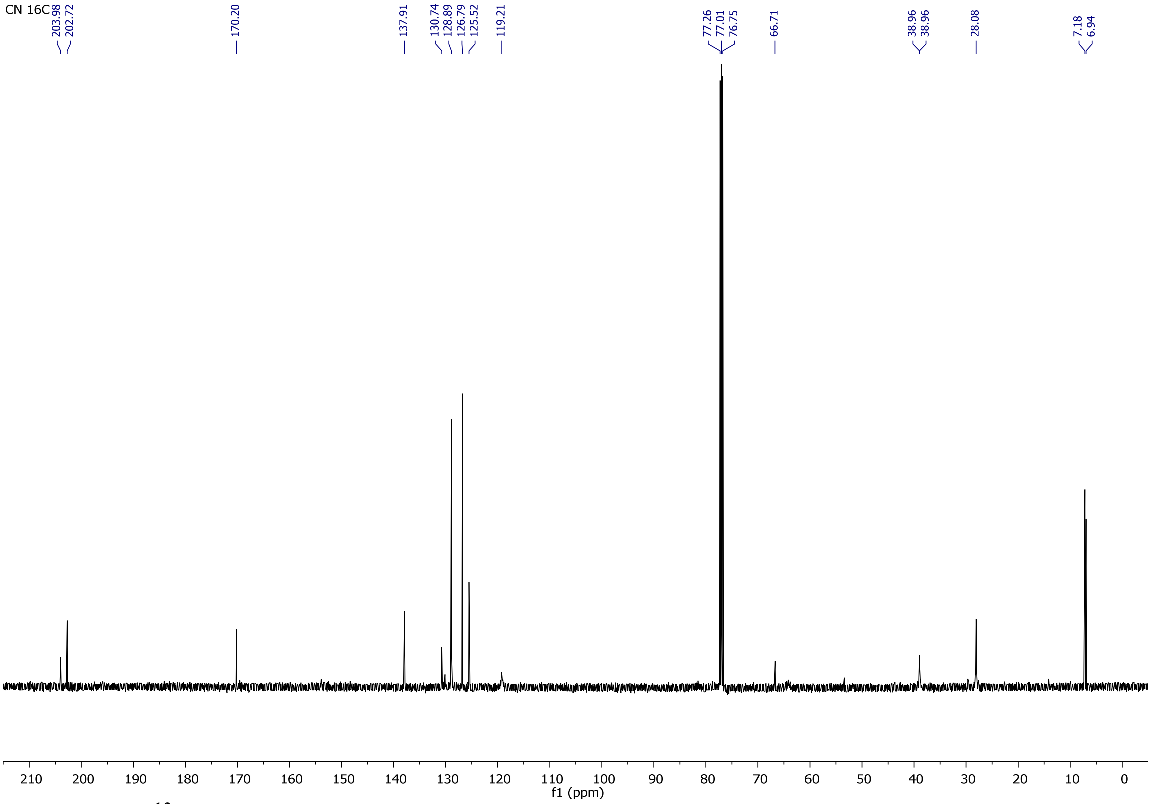

Figure S24. ^{13}C NMR spectrum of **3j** ($CDCl_3$, 126 MHz).

***tert*-Butyl (*S*)-(4-(1,3-dioxo-1,3-diphenylpropan-2-yl)-5-oxo-1,3-diphenyl-4,5-dihydro-1*H*-pyrazol-4-yl)carbamate (3k).**

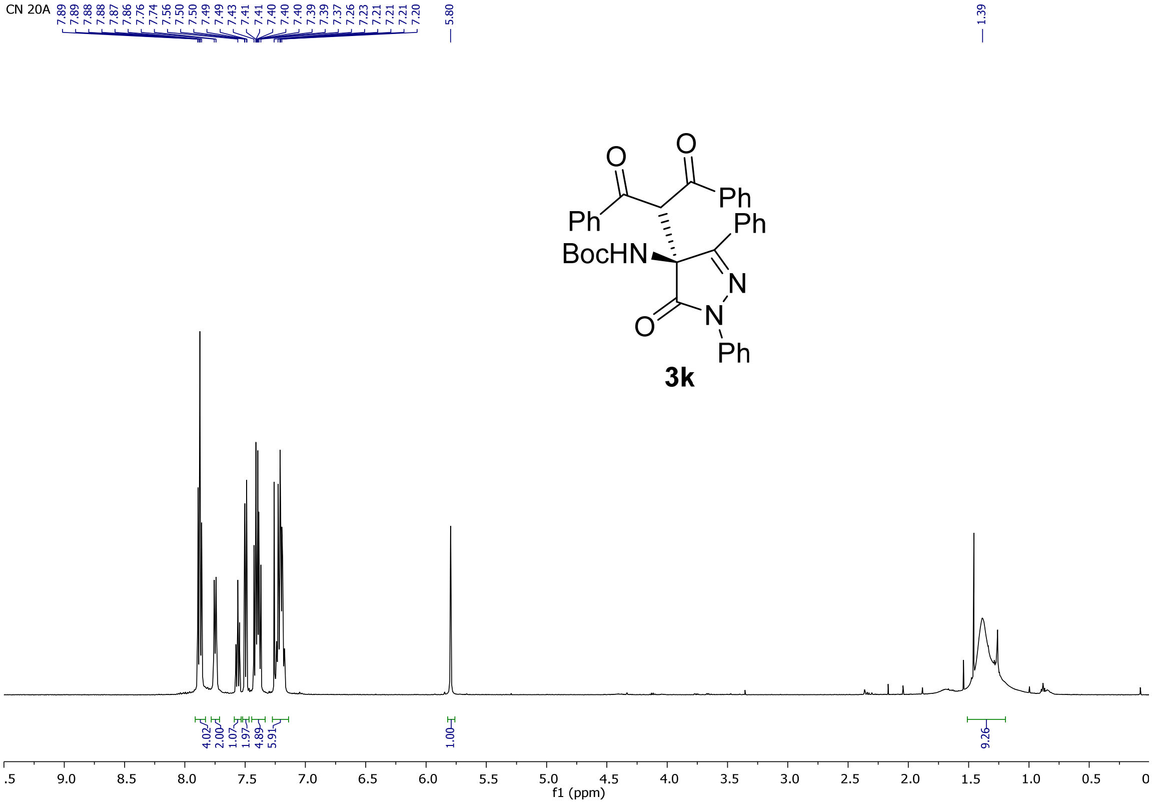

Figure S25. ^{1}H NMR spectrum of **3k** ($CDCl_3$, 500 MHz).

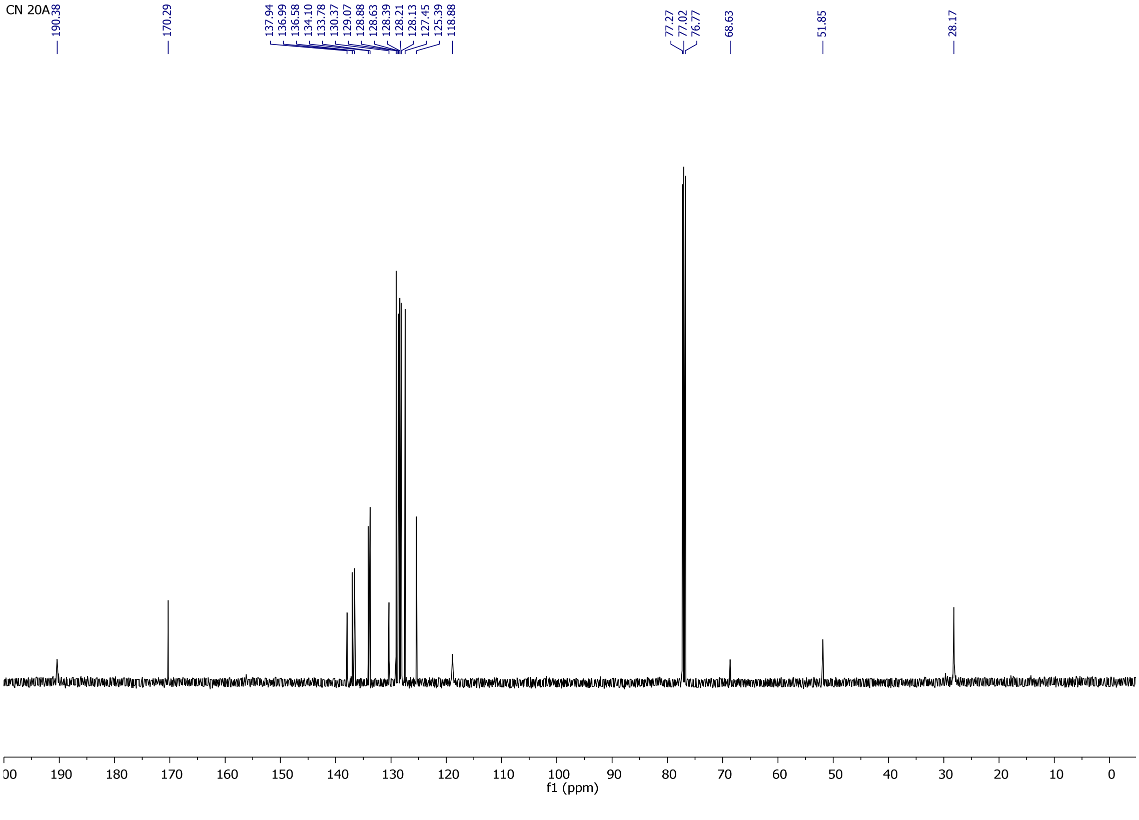

Figure S26. ^{13}C NMR spectrum of **3k** ($CDCl_3$, 126 MHz).

***tert*-Butyl (*S*)-(4-(2,4-dioxopentan-3-yl)-1-methyl-5-oxo-3-phenyl-4,5-dihydro-1*H*-pyrazol-4-yl)carbamate (3l).**

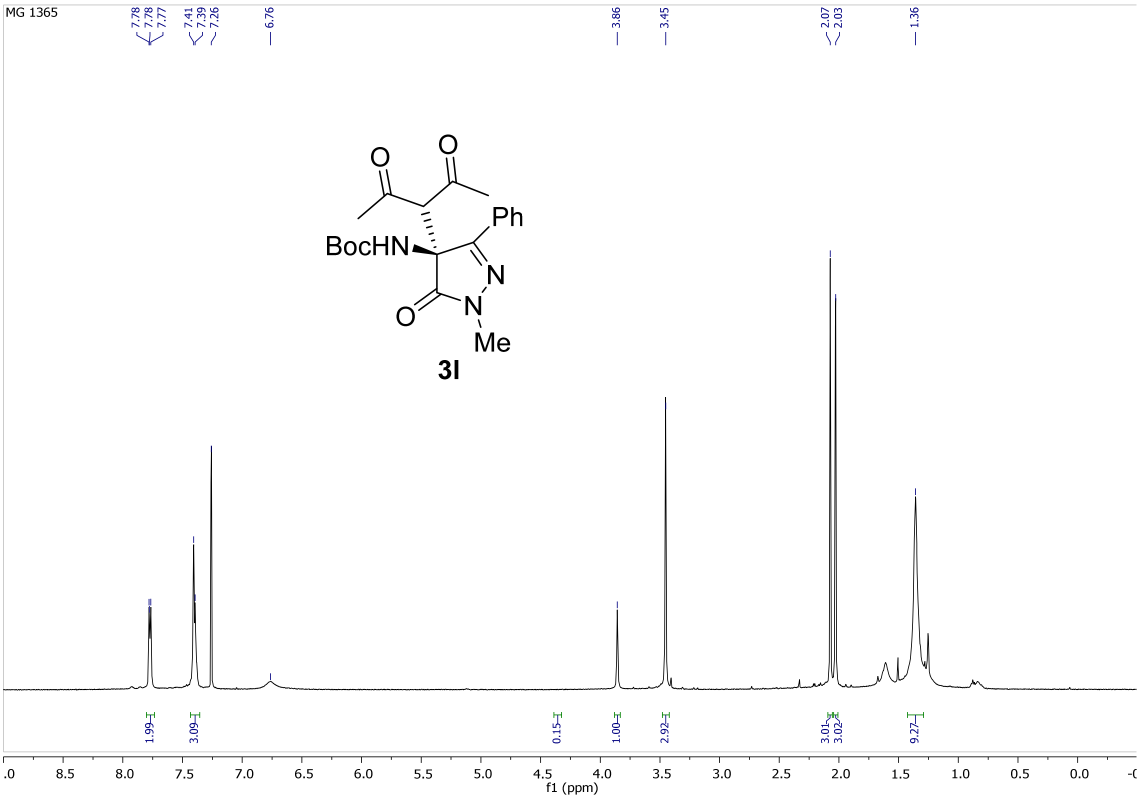

Figure S27. ^{1}H NMR spectrum of **3l** ($CDCl_3$, 500 MHz).

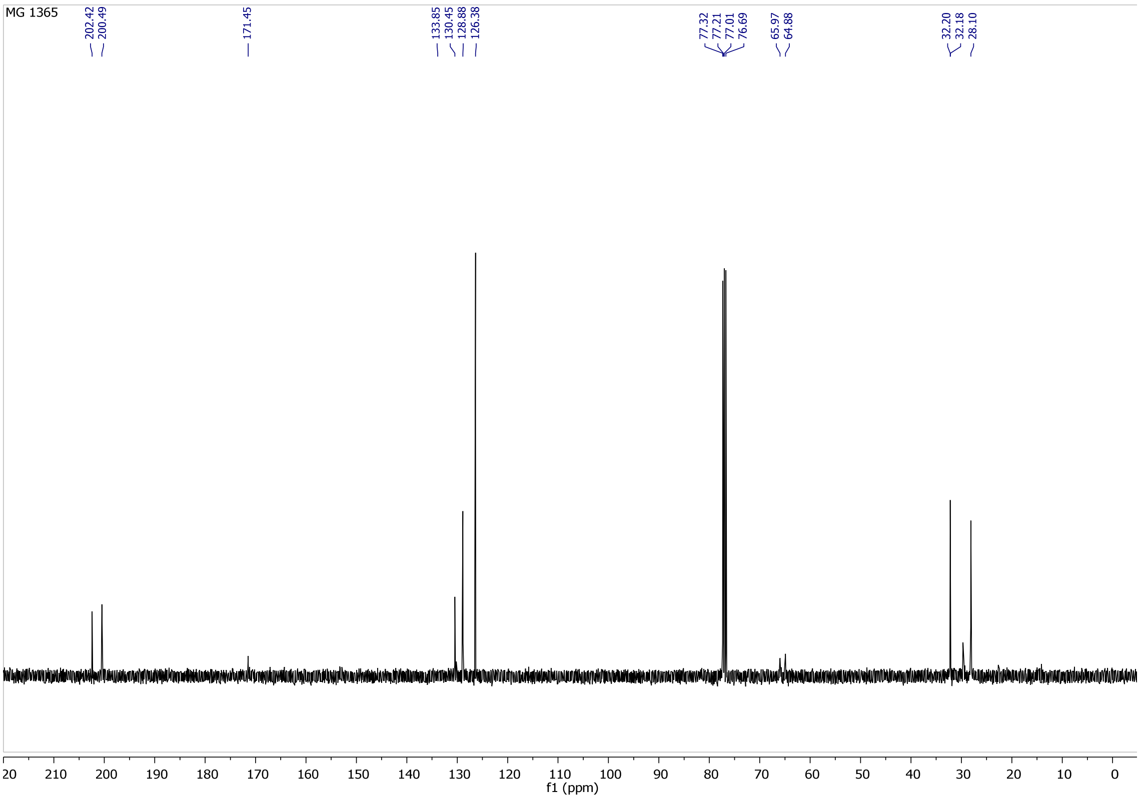

Figure S28. ^{13}C NMR spectrum of **3l** ($CDCl_3$, 126 MHz).

***tert*-Butyl (*S*)-(3,3',5-trimethyl-5'-oxo-1'-phenyl-1',5'-dihydro-1*H*,4'*H*-[4,4'-bipyrazol]-4'-yl)carbamate (4a).**

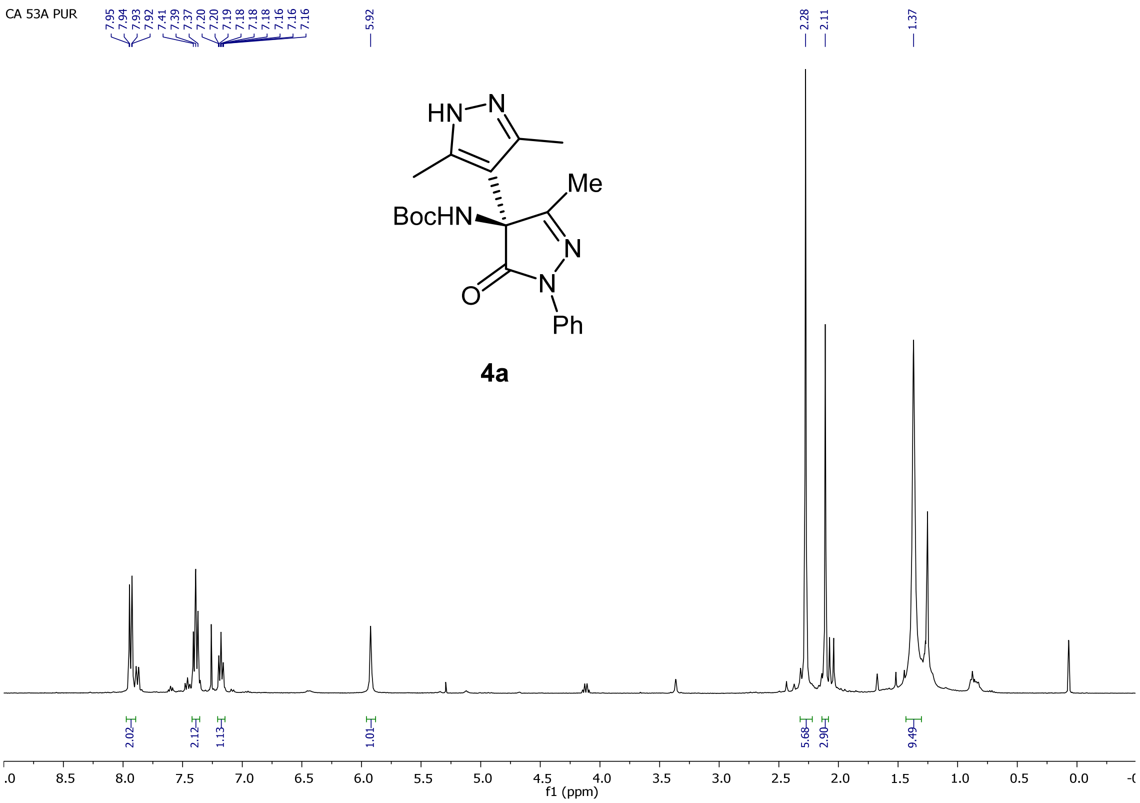

Figure S29. ^{1}H NMR spectrum of **4a** ($CDCl_3$, 400 MHz).

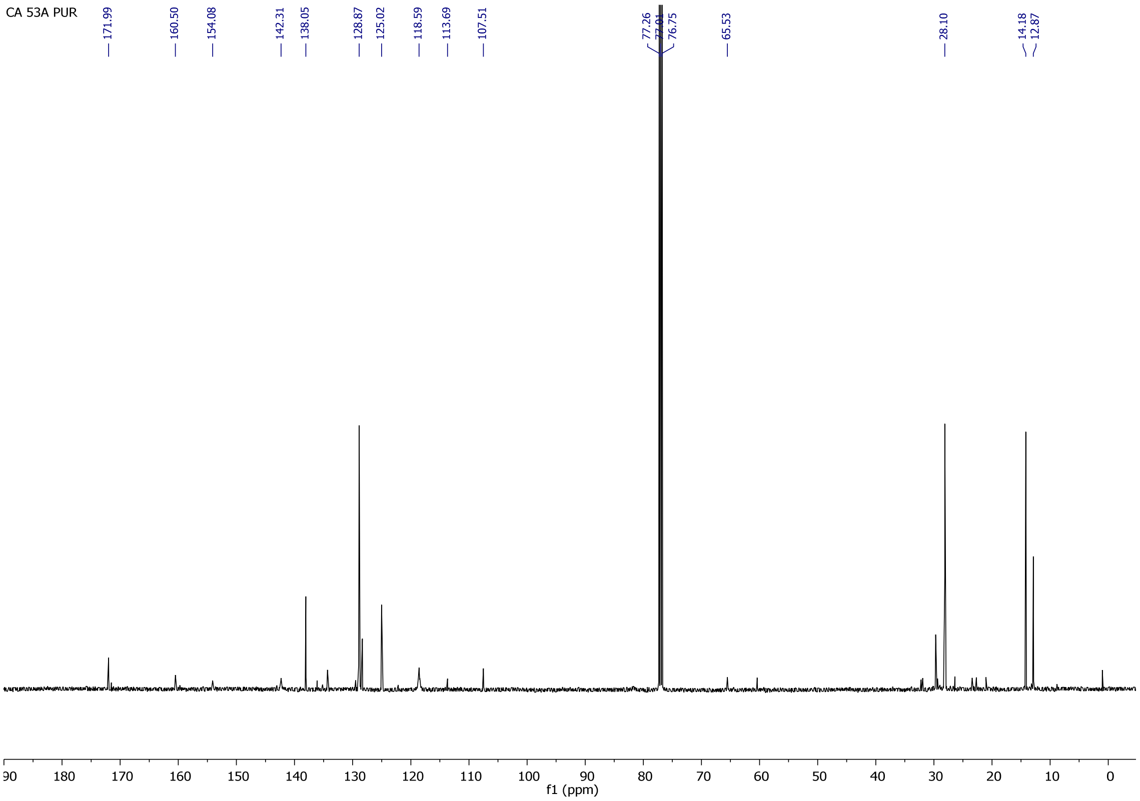

Figure S30. ^{13}C NMR spectrum of **4a** ($CDCl_3$, 126 MHz).

***tert*-Butyl (*S*)-(3'-ethyl-3,5-dimethyl-5'-oxo-1'-phenyl-1',5'-dihydro-1*H*,4'*H*-[4,4'-bipyrazol]-4'-yl)carbamate (4b).**

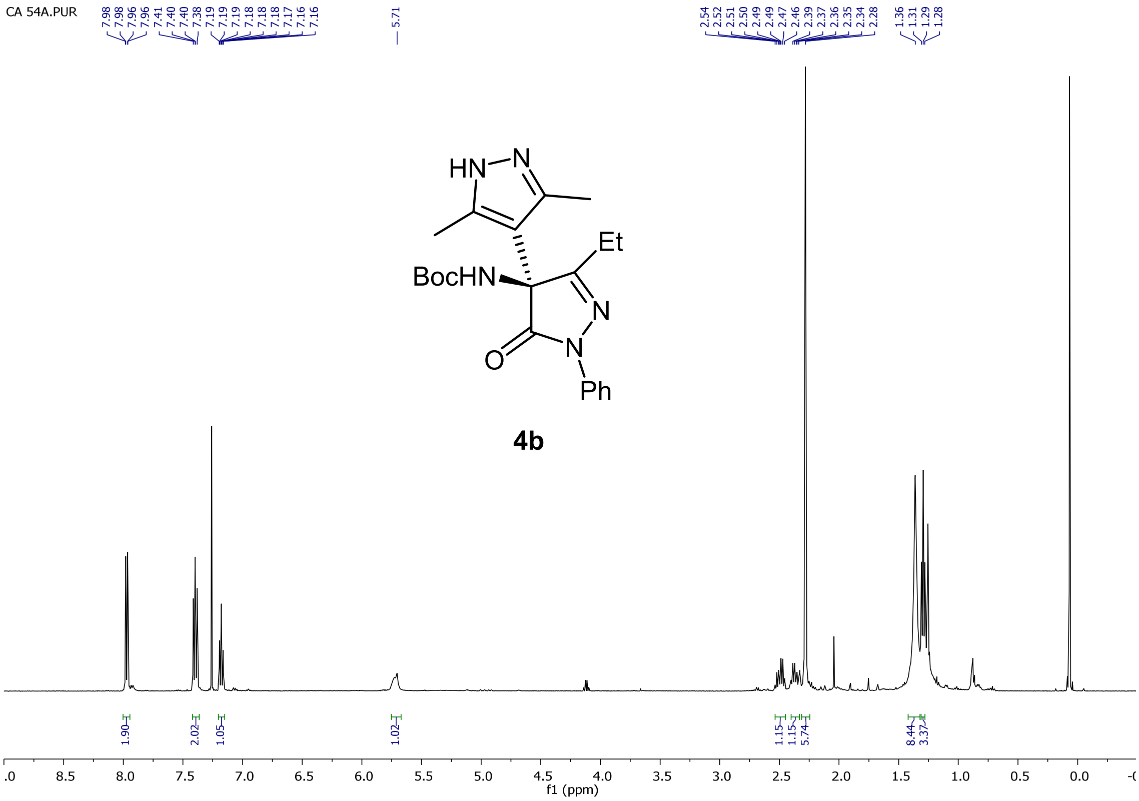

Figure S31. ^{1}H NMR spectrum of **4b** ($CDCl_3$, 500 MHz).

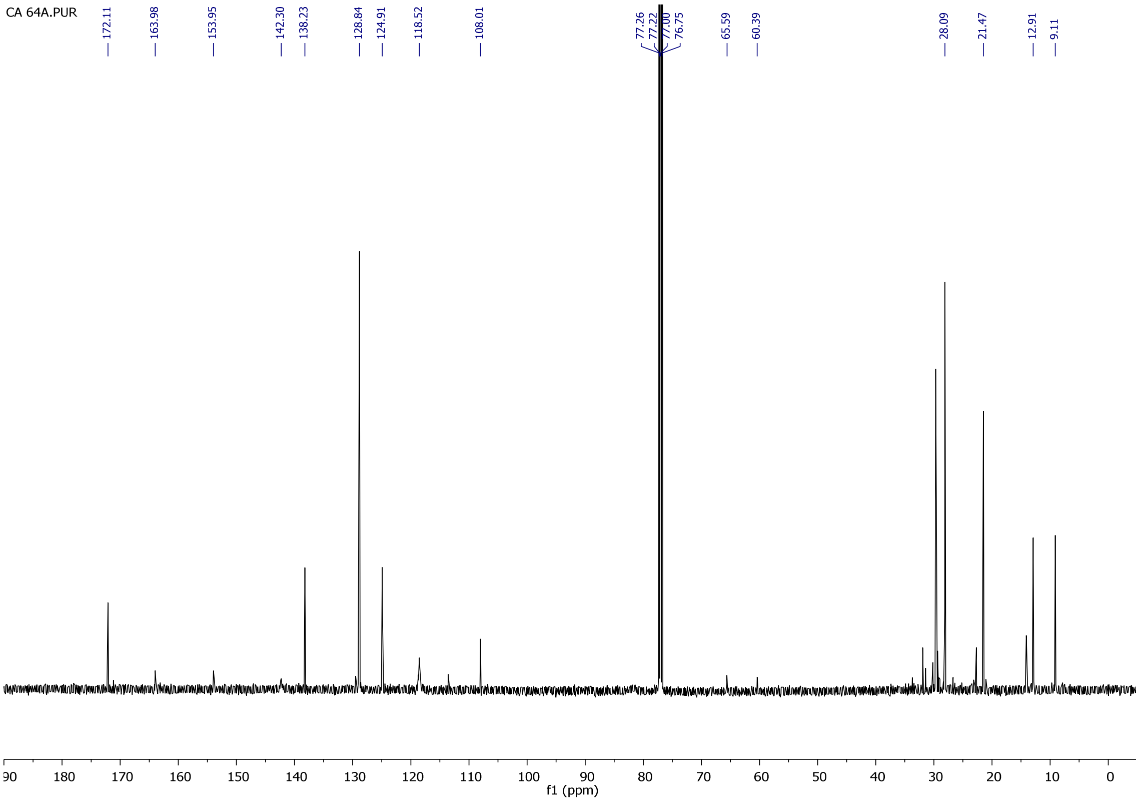

Figure S32. ^{13}C NMR spectrum of **4b** ($CDCl_3$, 126 MHz).

***tert*-Butyl (*S*)-(3'-isopropyl-3,5-dimethyl-5'-oxo-1'-phenyl-1',5'-dihydro-1*H*,4'*H*-[4,4'-bipyrazol]-4'-yl)carbamate (4c).**

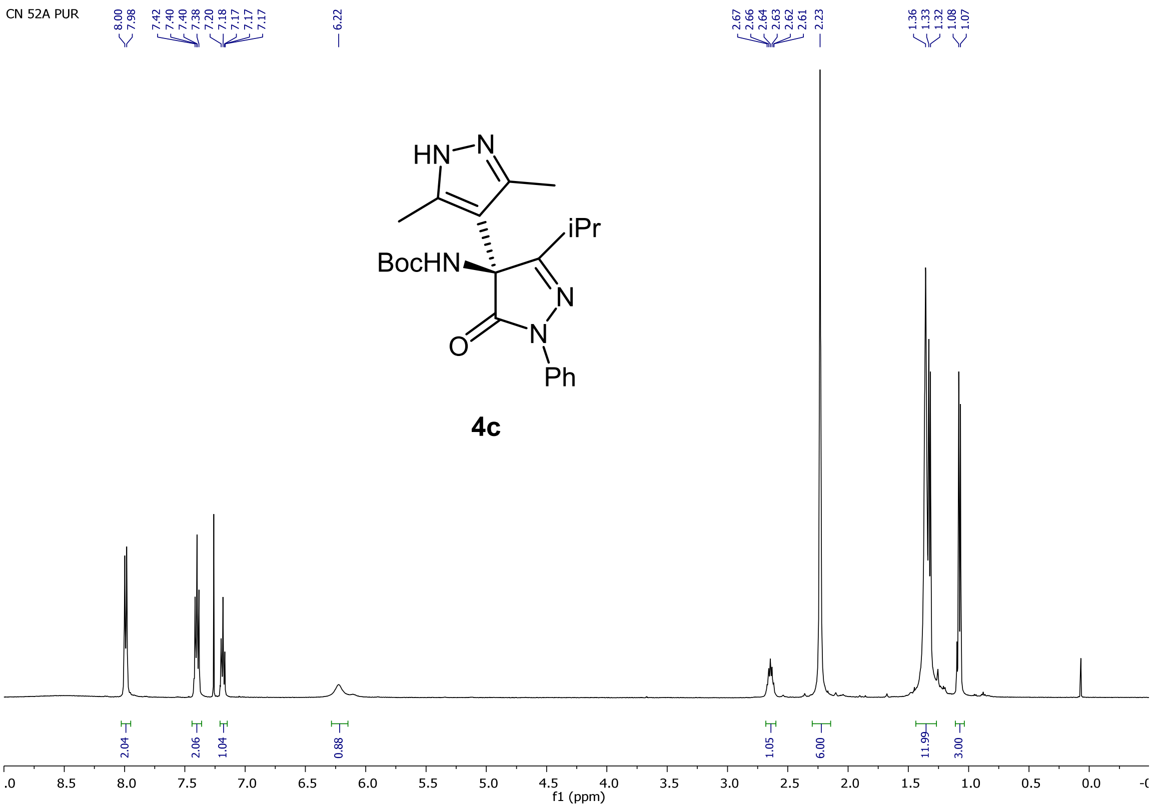

Figure S33. ^{1}H NMR spectrum of **4c** ($CDCl_3$, 500 MHz).

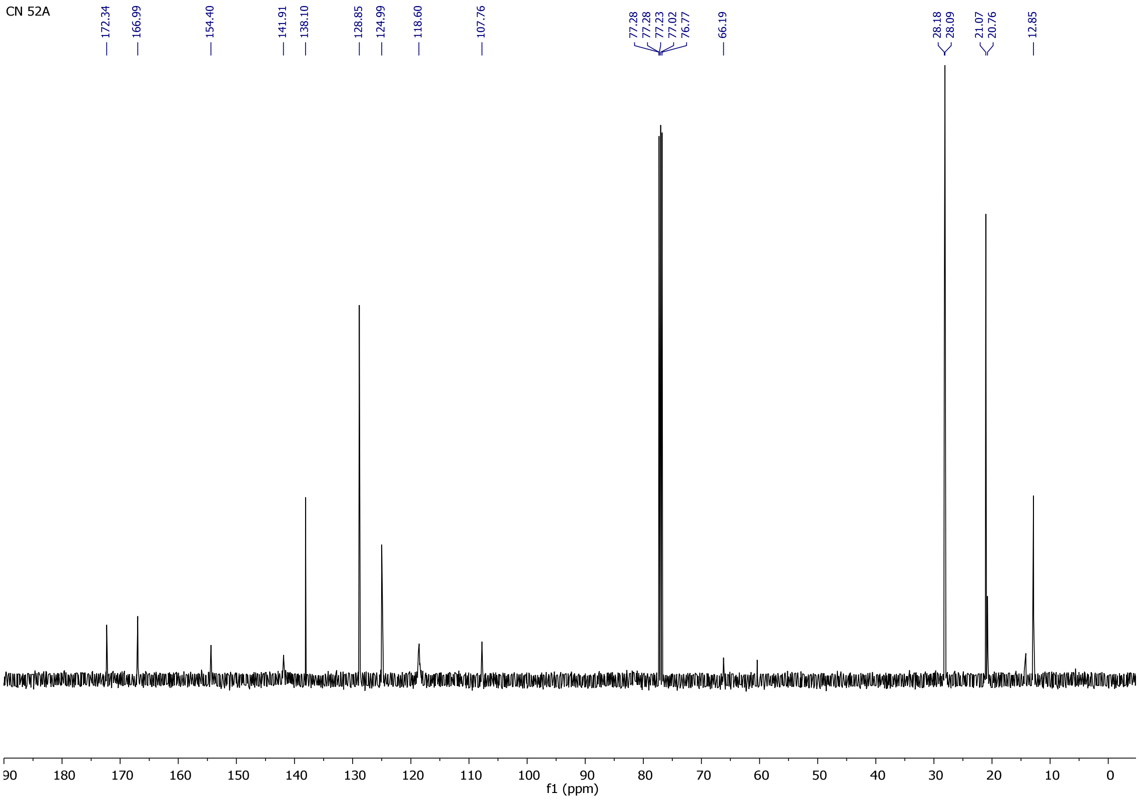

Figure S34. ^{13}C NMR spectrum of **4c** ($CDCl_3$, 126 MHz).

***tert*-Butyl (*S*)-(3,5-dimethyl-5'-oxo-1',3'-diphenyl-1',5'-dihydro-1*H*,4'*H*-[4,4'-bipyrazol]-4'-yl)carbamate (4e).**

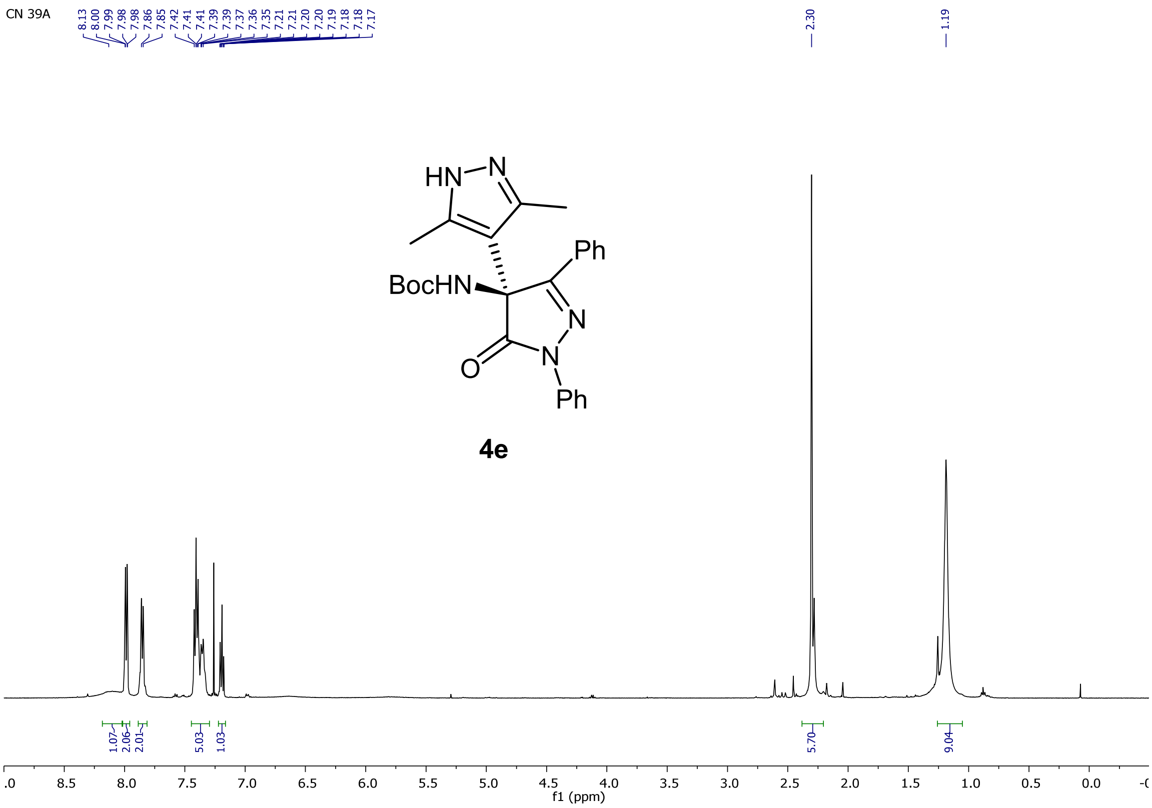

Figure S35. ^{1}H NMR spectrum of **4e** ($CDCl_3$, 500 MHz).

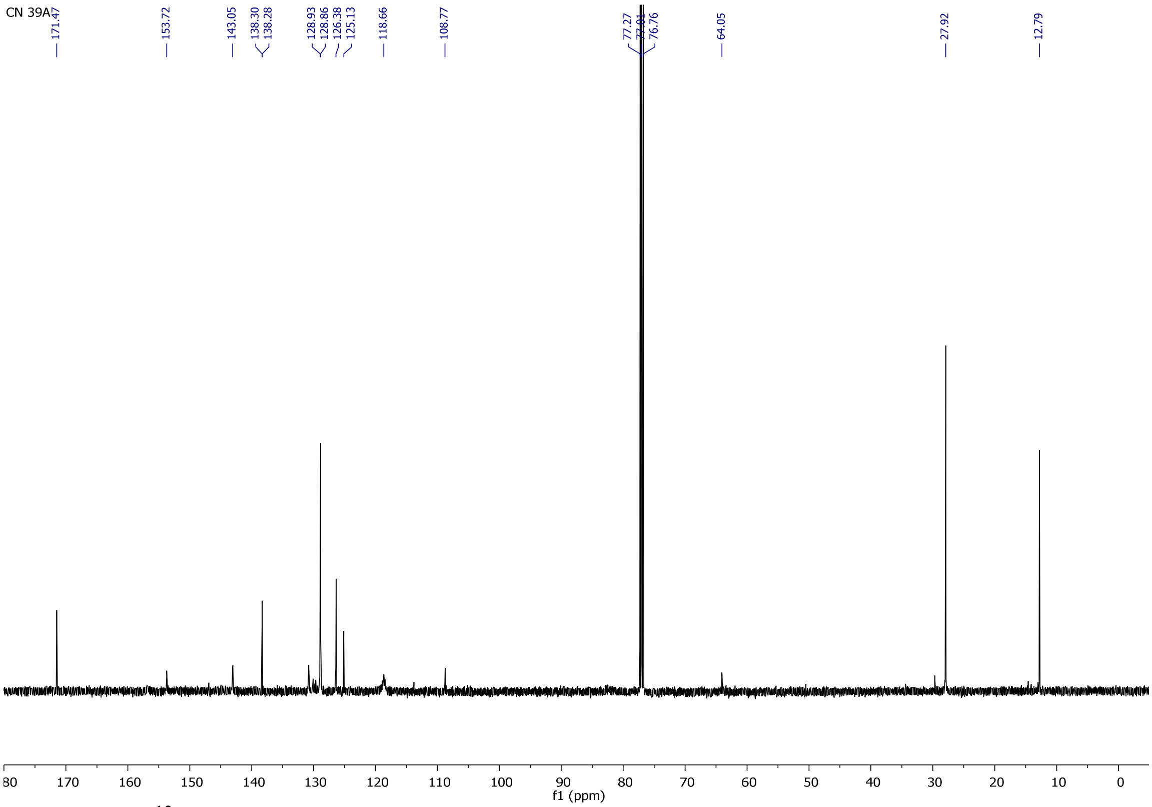

Figure S36. ^{13}C NMR spectrum of **4e** ($CDCl_3$, 126 MHz).

tert-Butyl (_S_)-(1'-(4-chlorophenyl)-3,3',5-trimethyl-5'-oxo-1',5'-dihydro-1_H_,4'_H_-[4,4'-bipyrazol]-4'-yl)carbamate (4f).

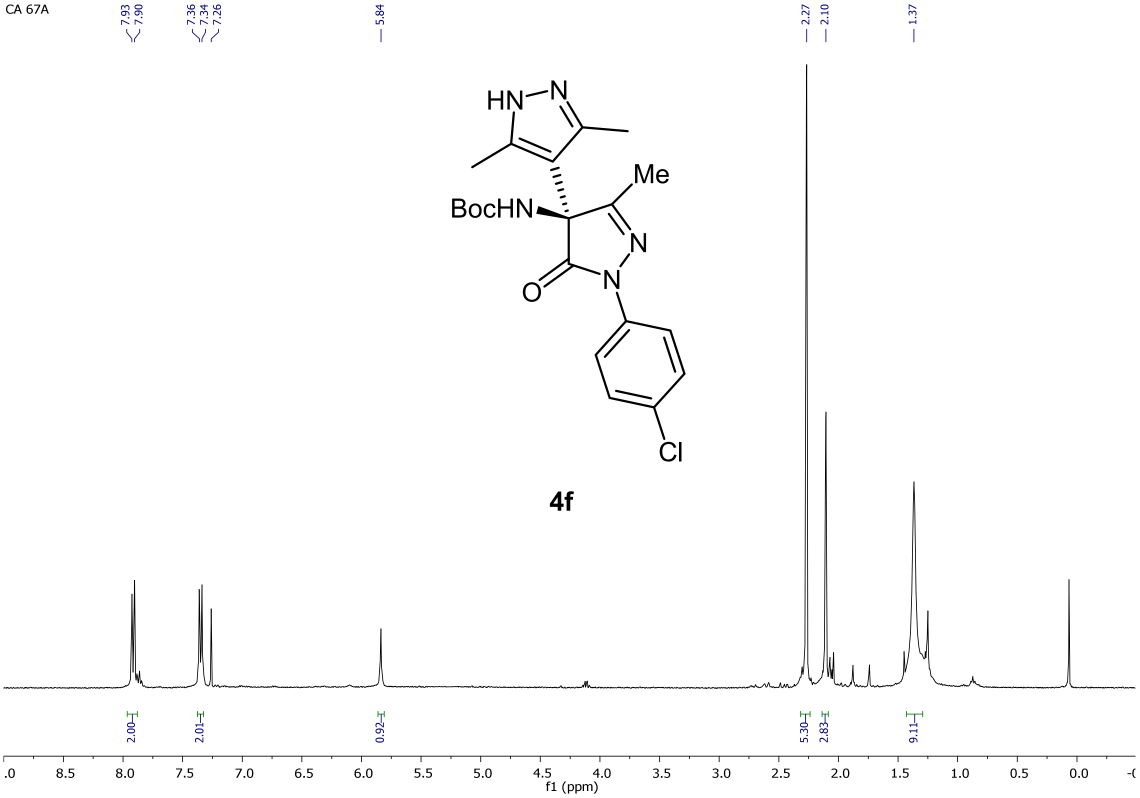

Figure S37. ^{1}H NMR spectrum of **4f** ($CDCl_3$, 500 MHz).

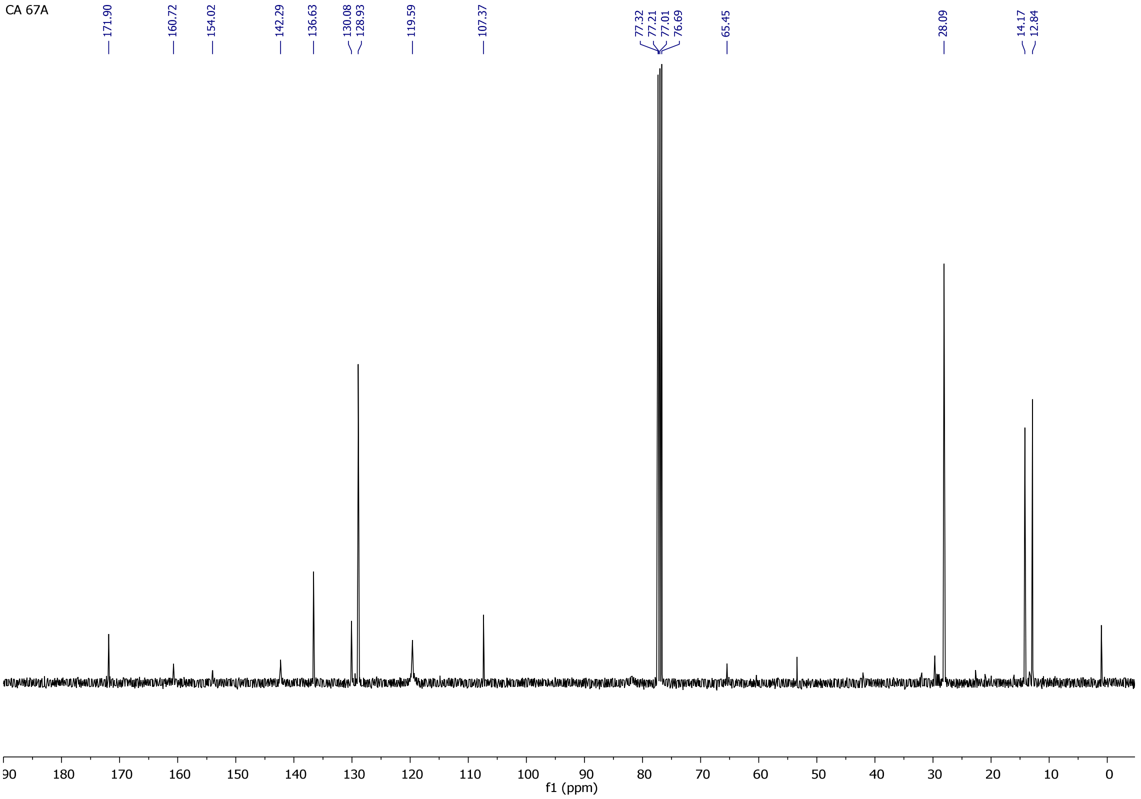

Figure S38. ^{13}C NMR spectrum of **4f** ($CDCl_3$, 126 MHz).

***tert*-Butyl (*S*)-(3,3',5-trimethyl-5'-oxo-1'-(*p*-tolyl)-1',5'-dihydro-1*H*,4'*H*-[4,4'-bipyrazol]-4'-yl)carbamate (4g).**

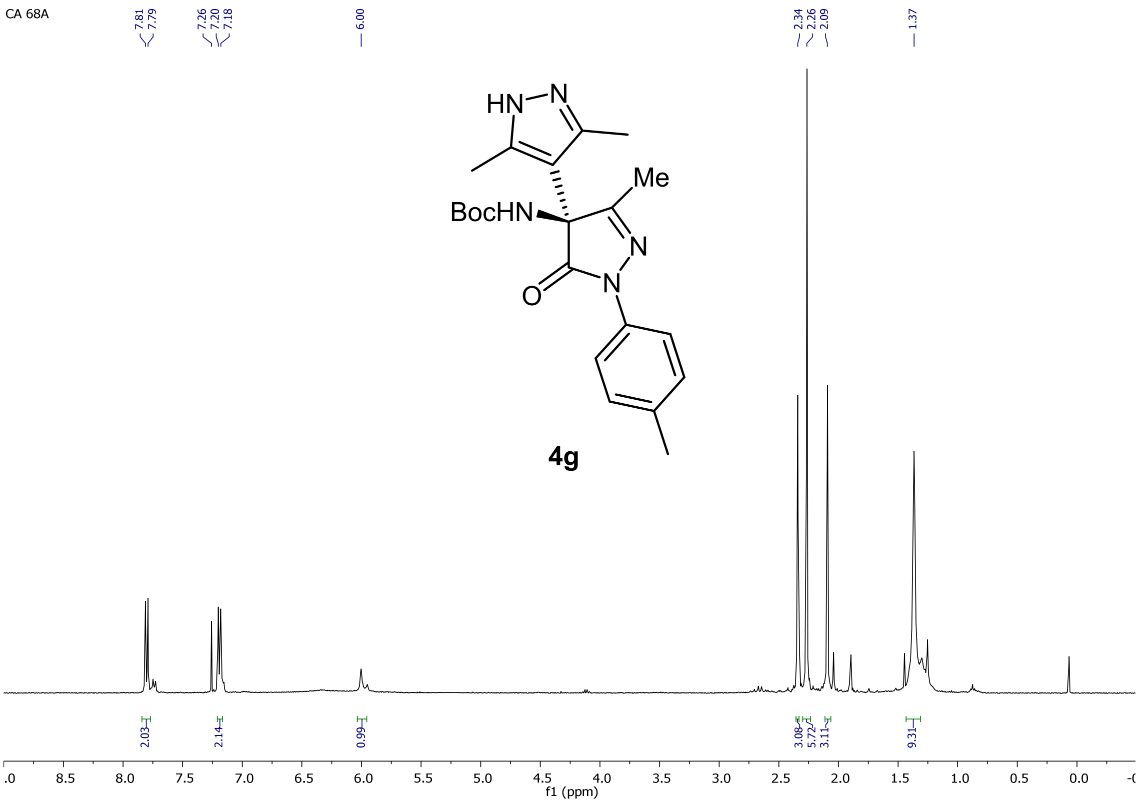

Figure S39. ^{1}H NMR spectrum of **4g** ($CDCl_3$, 500 MHz).

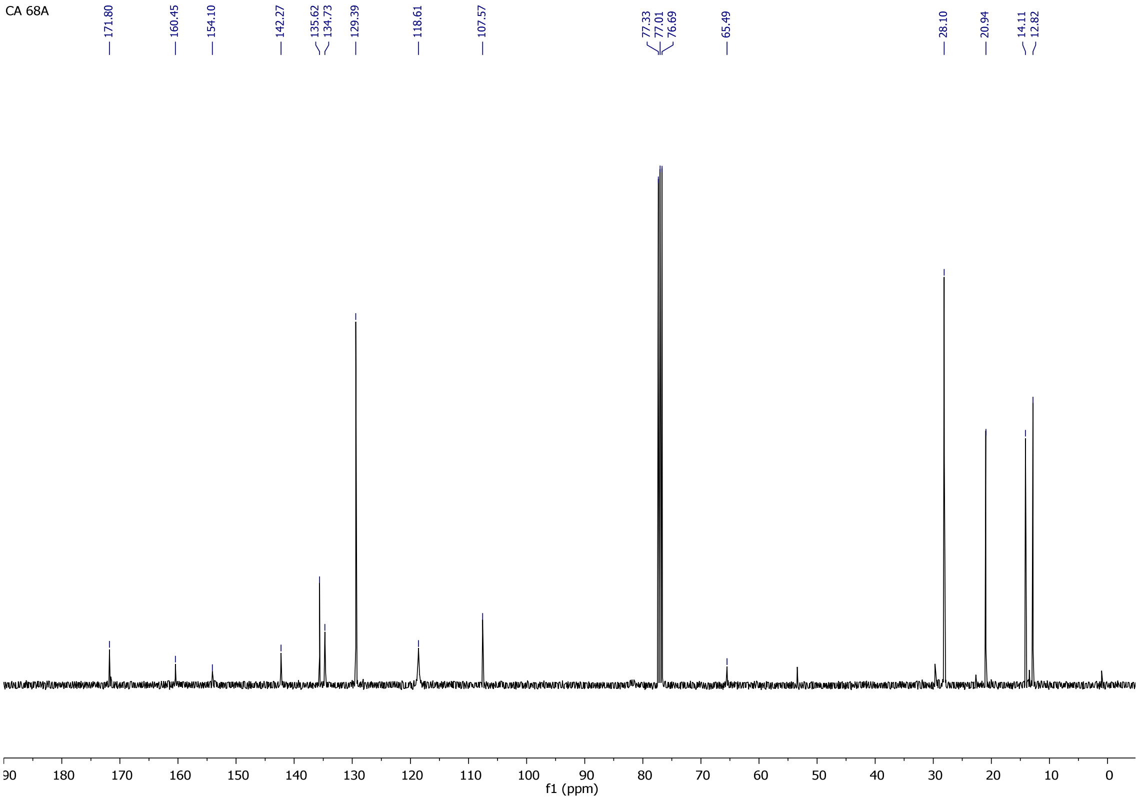

Figure S40. ^{13}C NMR spectrum of **4g** ($CDCl_3$, 126 MHz).

***tert*-Butyl (*S*)-(3,5-diethyl-5'-oxo-1',3'-diphenyl-1',5'-dihydro-1*H*,4'*H*-[4,4'-bipyrazol]-4'-yl)carbamate (4j).**

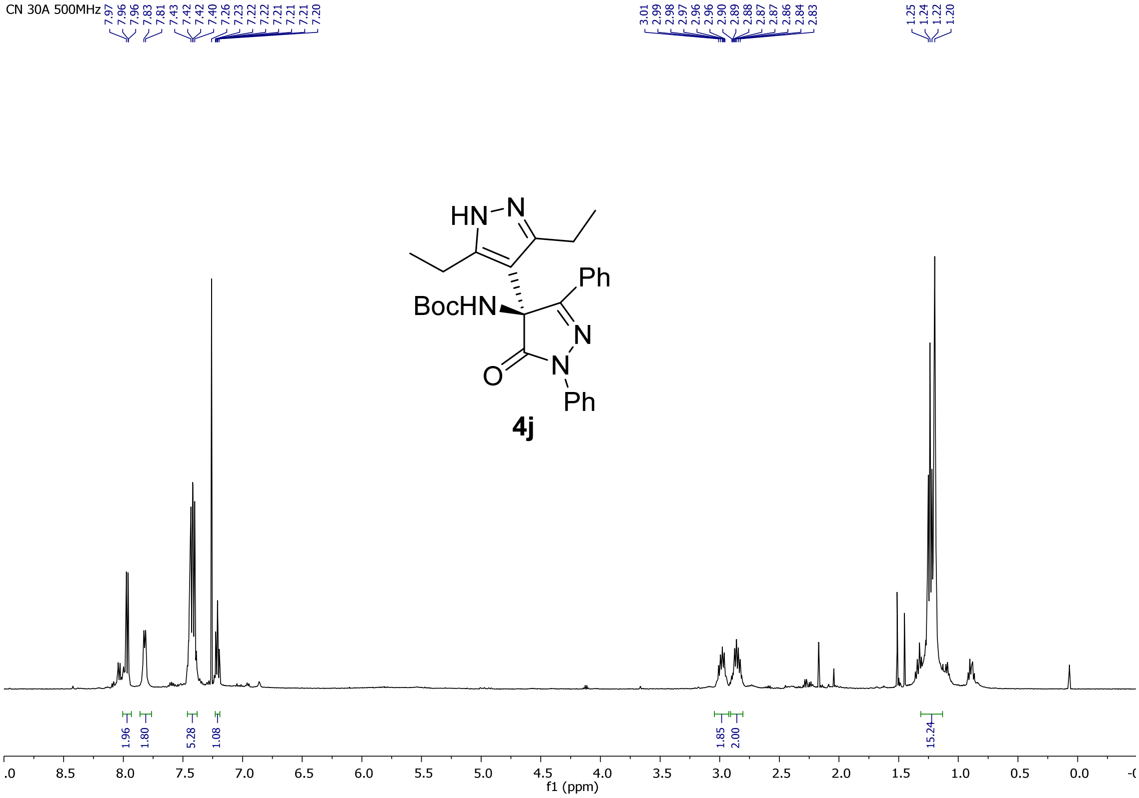

Figure S41. ^{1}H NMR spectrum of **4j** ($CDCl_3$, 500 MHz).

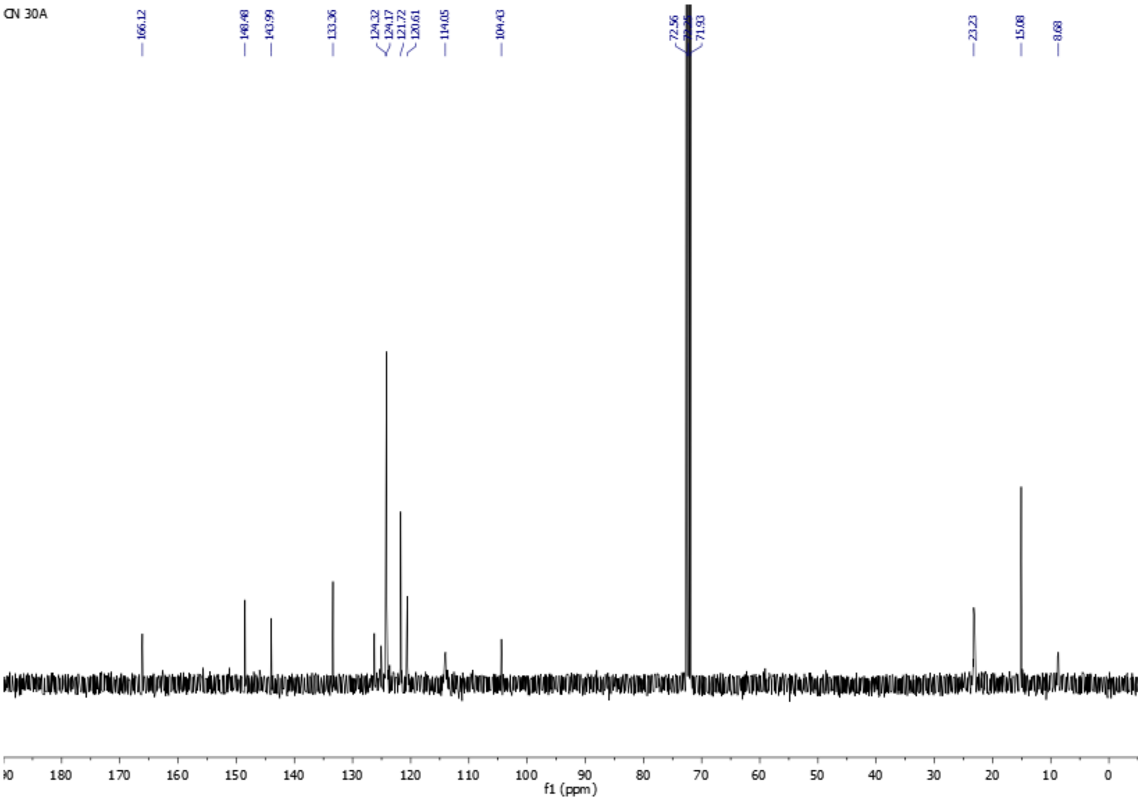

Figure S42. ^{13}C NMR spectrum of **4j** ($CDCl_3$, 126 MHz).

***tert*-Butyl (*S*)-(3-methyl-5-oxo-4-(2-oxo-2-phenylethyl)-1-phenyl-4,5-dihydro-1*H*-pyrazol-4-yl)carbamate (5i).**

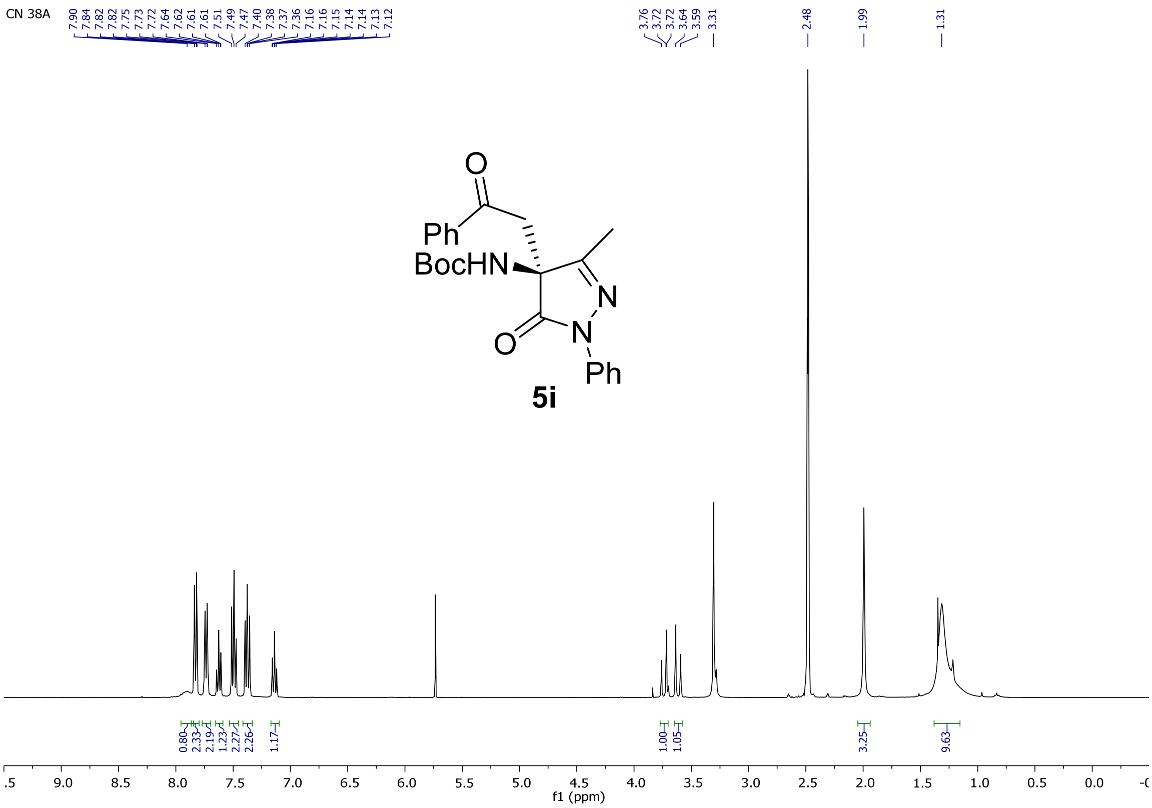

Figure S43. ^{1}H NMR spectrum of **5i** (DMSO-d$_6$, 400 MHz).

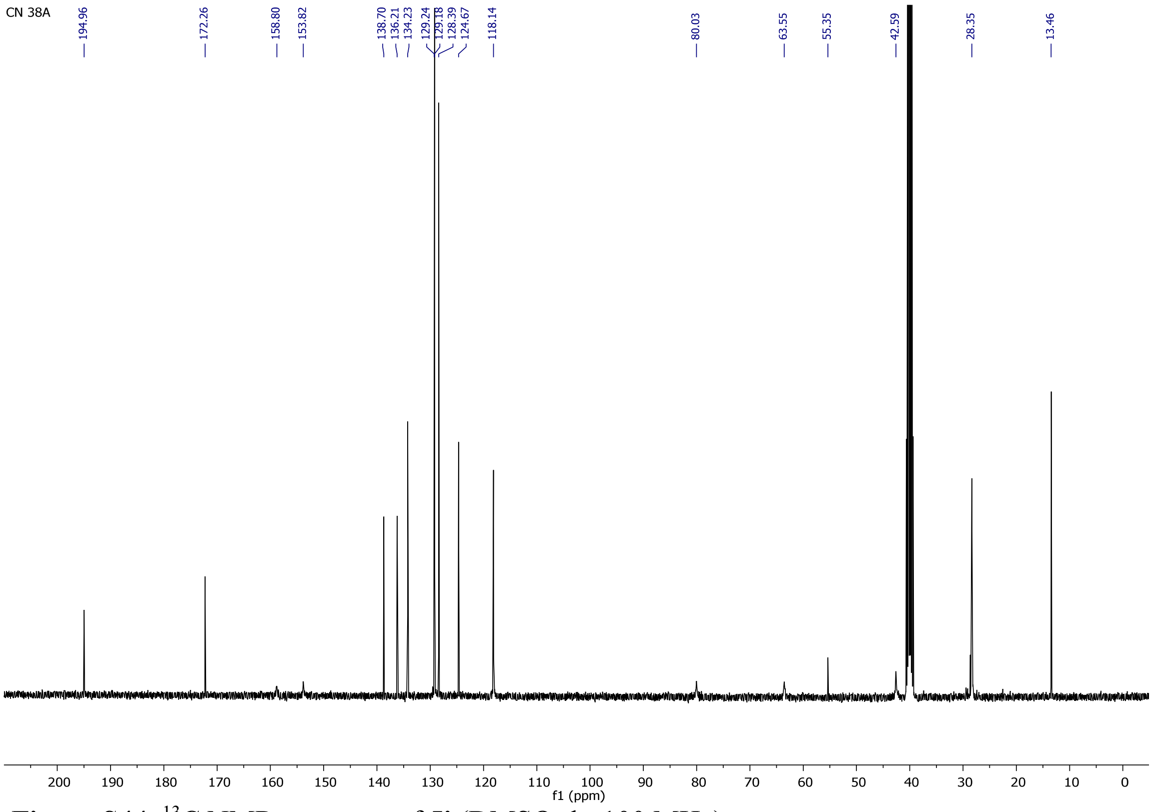

Figure S44. ^{13}C NMR spectrum of **5i** (DMSO-d$_6$, 100 MHz).

tert-Butyl (_S_)-(1-(4-chlorophenyl)-3,3',5-trimethyl-5'-oxo-1'-phenyl-1',5'-dihydro-1_H_,4'_H_-[4,4'-bipyrazol]-4'-yl)carbamate (6a).

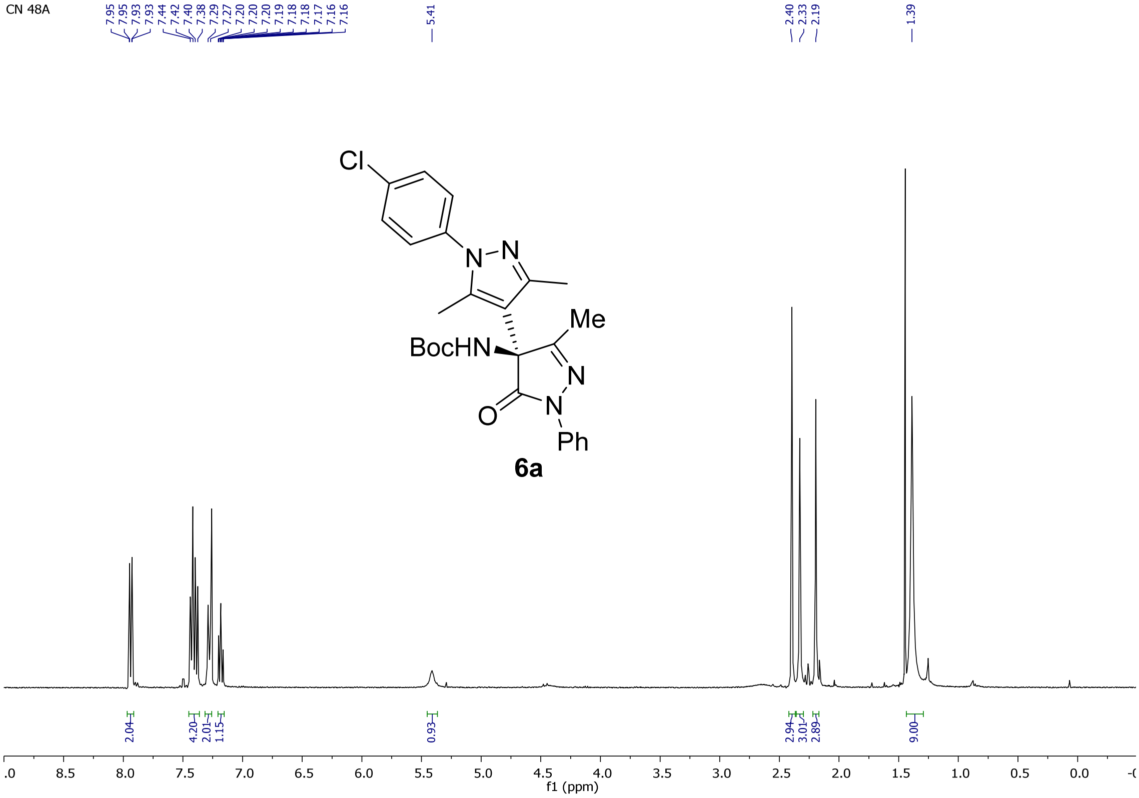

Figure S45. ^{1}H NMR spectrum of **6a** ($CDCl_3$, 500 MHz).

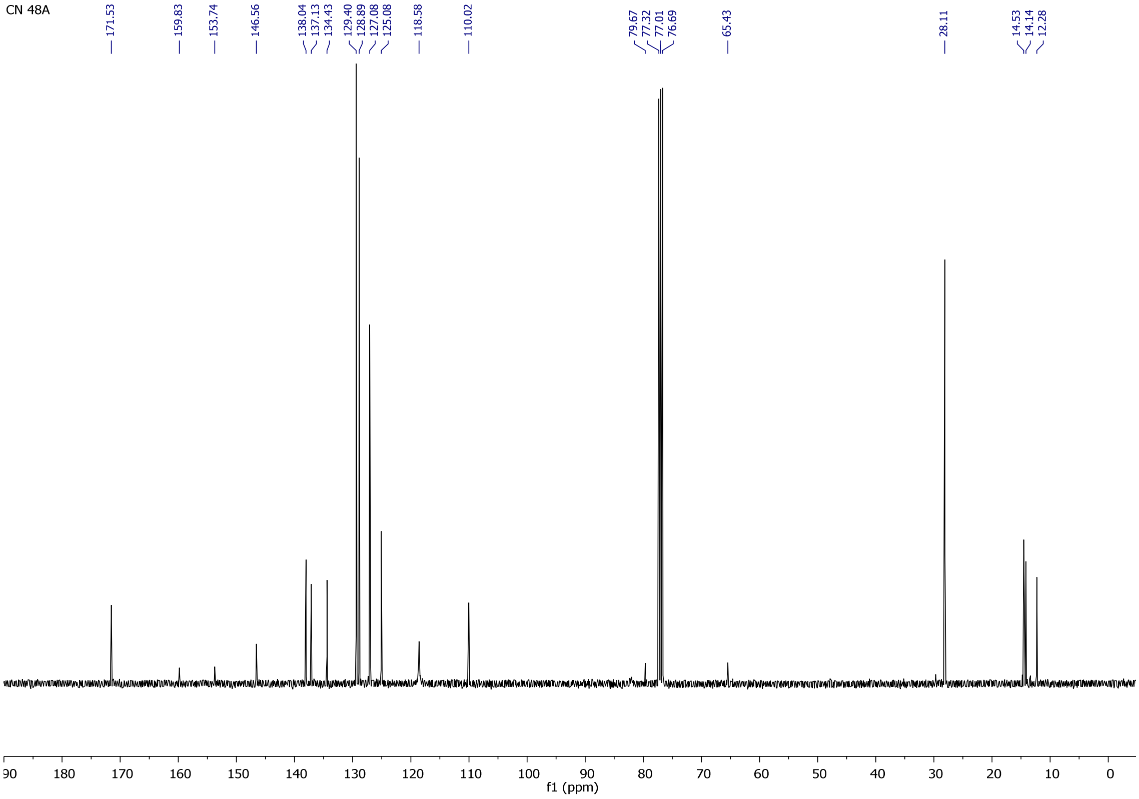

Figure S46. ^{13}C NMR spectrum of **6a** ($CDCl_3$, 126 MHz).

***tert*-Butyl (*S*)-(1-(4-chlorophenyl)-3,5-dimethyl-5'-oxo-1',3'-diphenyl-1',5'-dihydro-1*H*,4'*H*-[4,4'-bipyrazol]-4'-yl)carbamate (6e).**

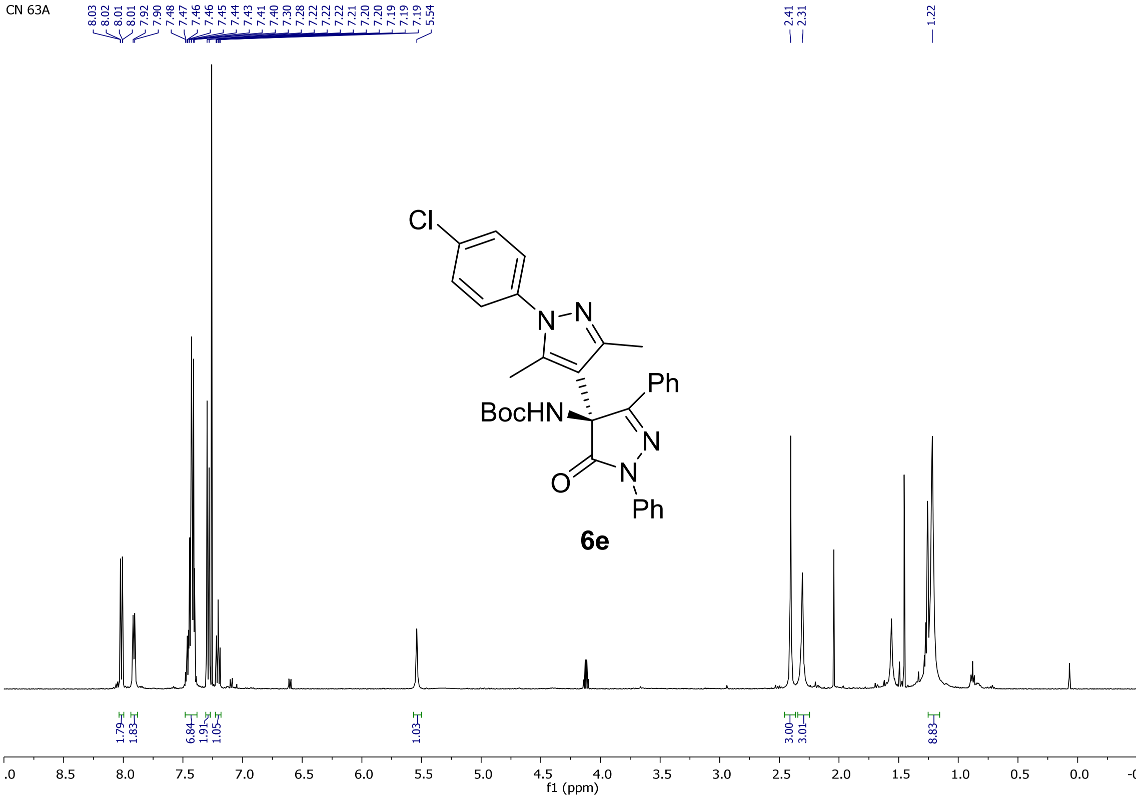

Figure S47. ^{1}H NMR spectrum of **6e** ($CDCl_3$, 500 MHz).

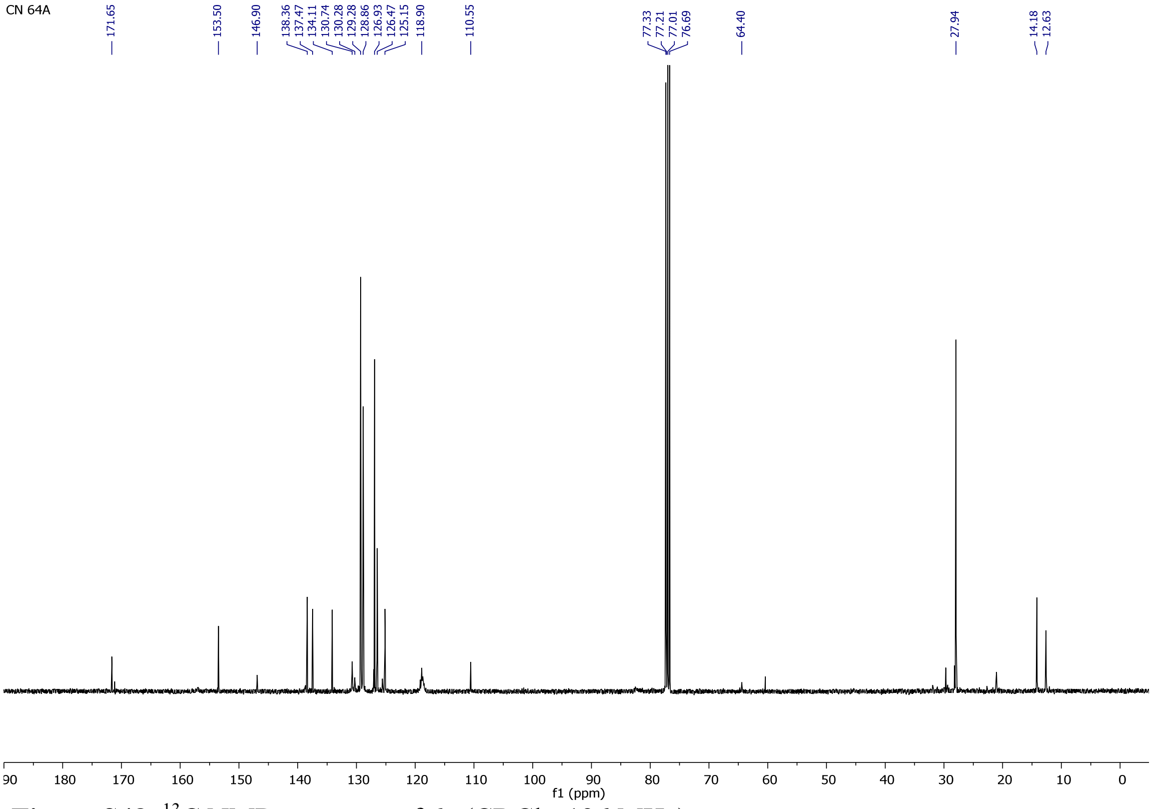

Figure S48. ^{13}C NMR spectrum of **6e** ($CDCl_3$, 126 MHz).

***tert*-Butyl (*S*)-(4-(3,5-dimethylisoxazol-4-yl)-3-methyl-5-oxo-1-phenyl-4,5-dihydro-1*H*-pyrazol-4-yl)carbamate (7a).**

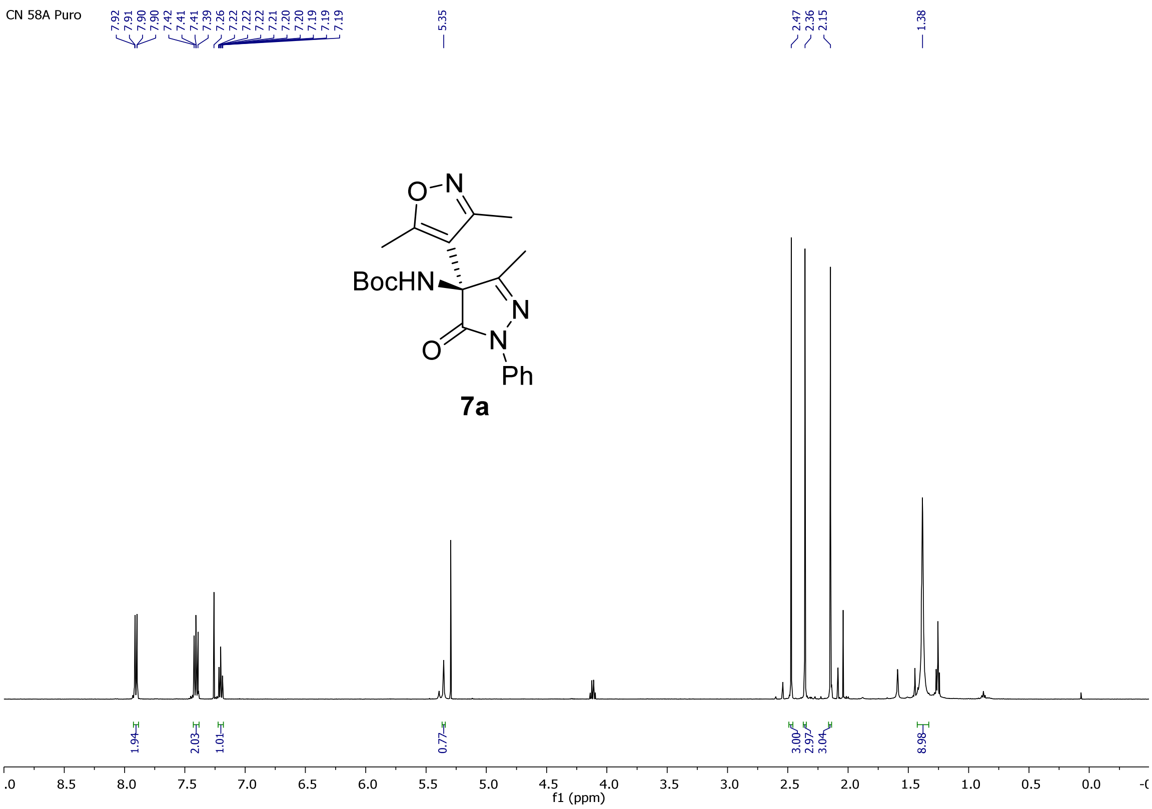

Figure S49. ^{1}H NMR spectrum of **7a** ($CDCl_3$, 500 MHz).

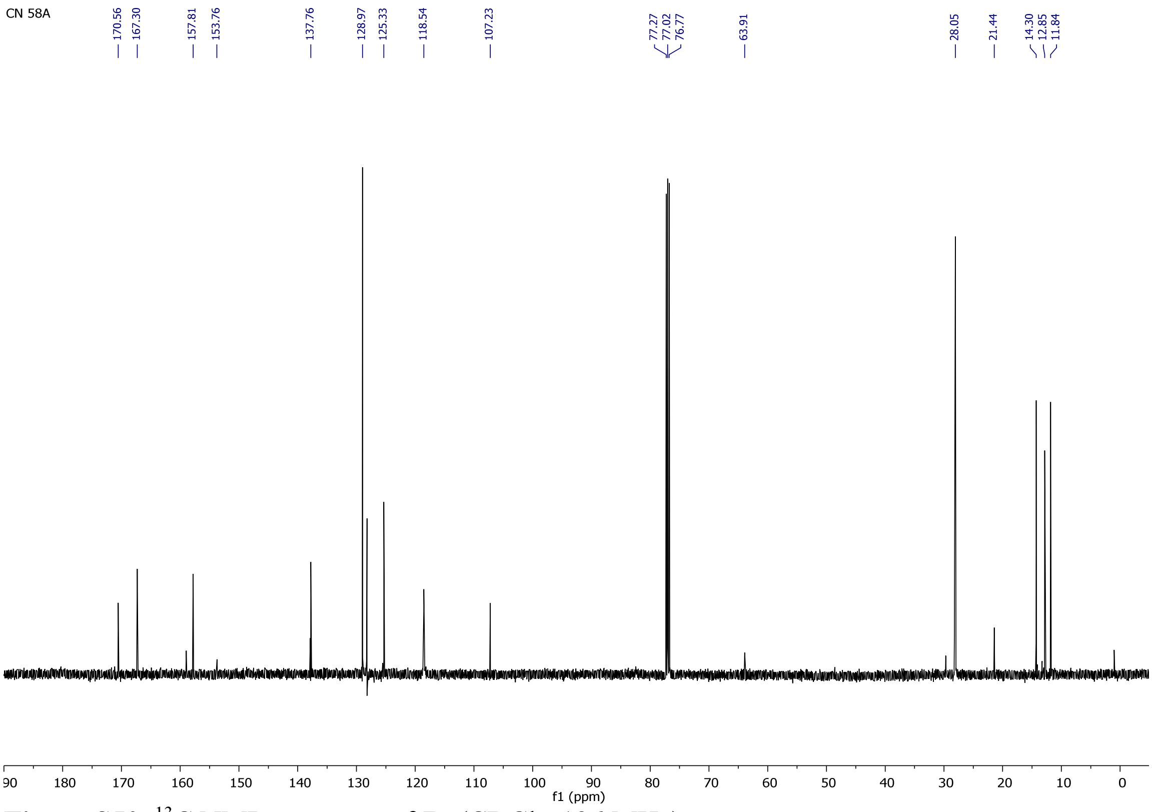

Figure S50. ^{13}C NMR spectrum of **7a** ($CDCl_3$, 126 MHz).

tert-Butyl (_S_)-(4-(3,5-dimethylisoxazol-4-yl)-5-oxo-1,3-diphenyl-4,5-dihydro-1_H_-pyrazol-4-yl)carbamate (7e).

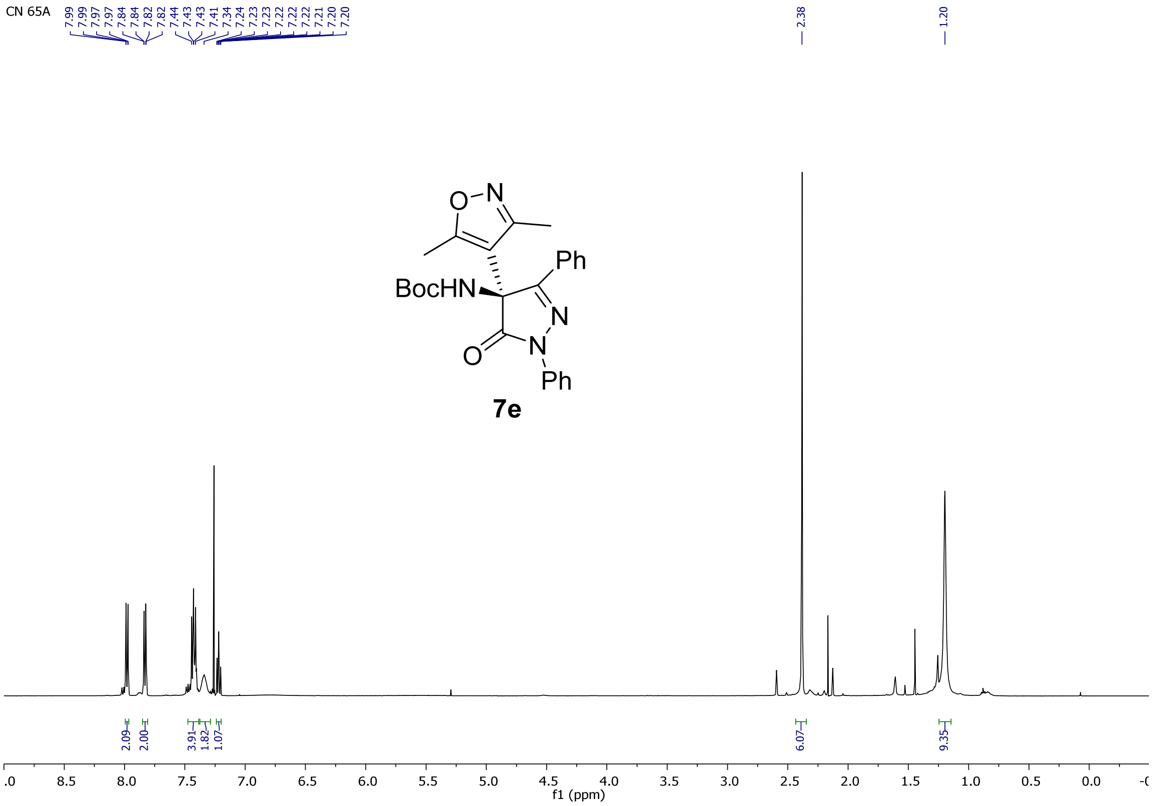

Figure S51. ^{1}H NMR spectrum of **7e** ($CDCl_3$, 500 MHz).

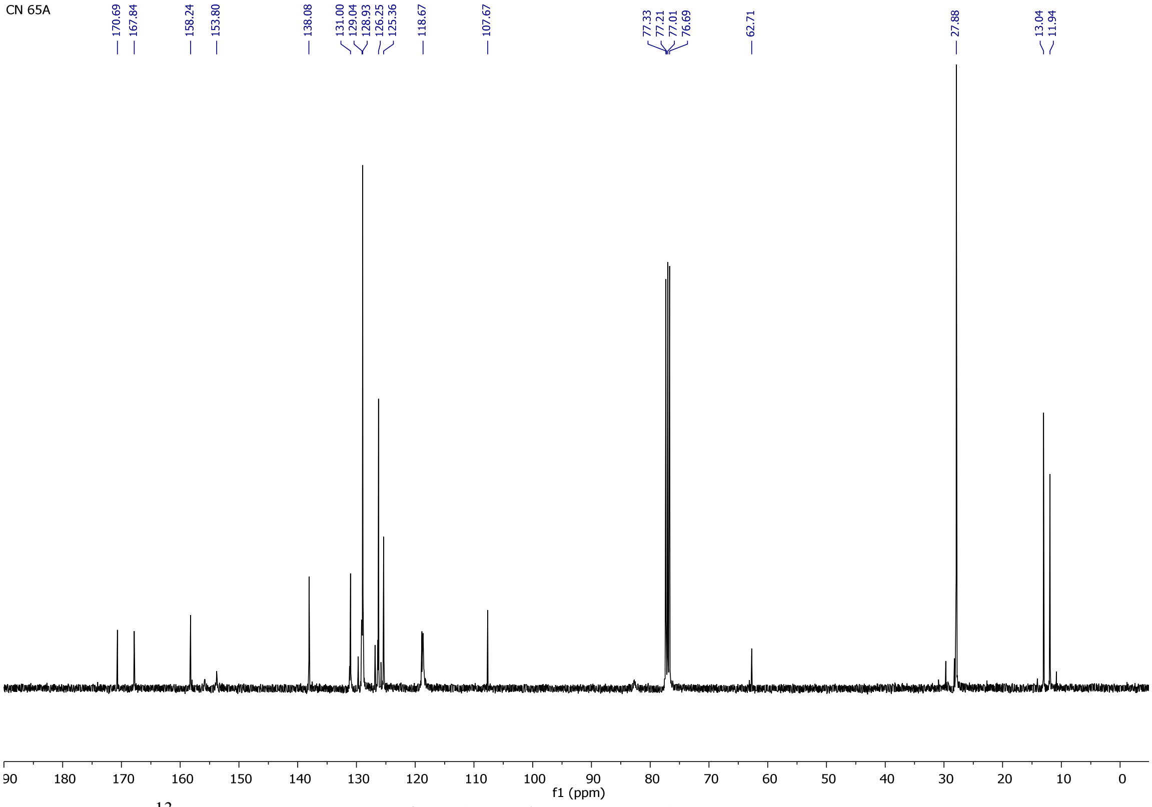

Figure S52. ^{13}C NMR spectrum of **7e** ($CDCl_3$, 100 MHz).

3. HPLC Profiles of the isolated compounds.

***tert*-Butyl (*S*)-(4-(2,4-dioxopentan-3-yl)-3-methyl-5-oxo-1-phenyl-4,5-dihydro-1*H*-pyrazol-4-yl)carbamate (3a).**

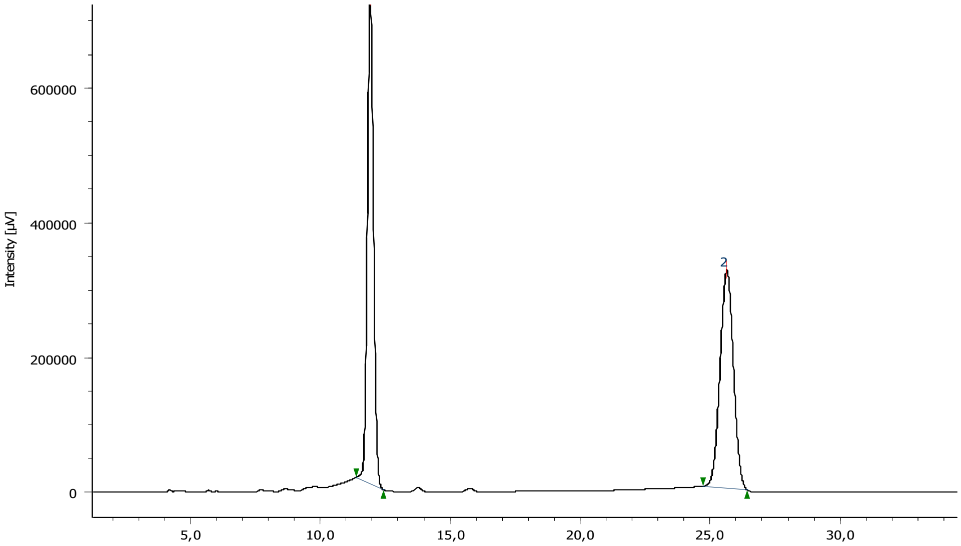

Peak Number	t_R(min)	Area	Height	Area (%)	Symmetry Factor
1	**11.900**	11954241	722346	**50.613**	1.104
2	**25.608**	11664640	323407	**49.387**	1.005

Figure S53. HPLC profile for **3a** (racemic).

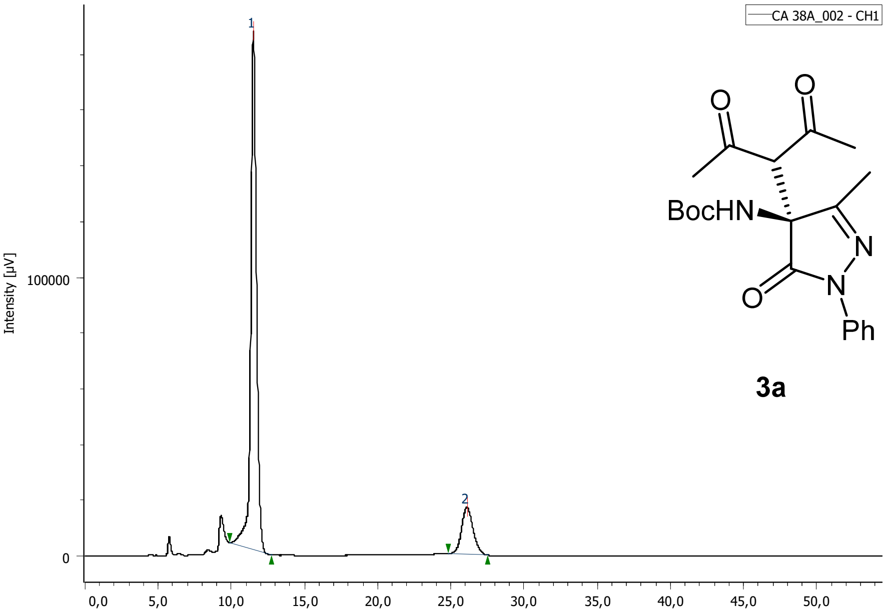

Peak Number	t_R(min)	Area	Height	Area (%)	Symmetry Factor
1	**11.492**	5090147	185674	**84.771**	1.000
2	**26.058**	914451	16821	**15.229**	1.136

Figure S54. HPLC Profile for **3a** compound. Scheme 2, er 85:15.

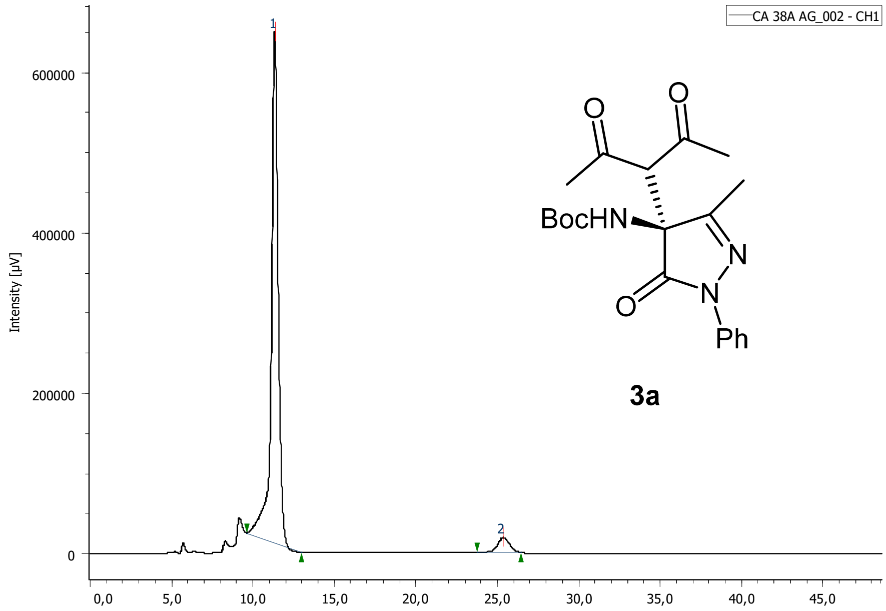

Peak Number	t_R(min)	Area	Height	Area (%)	Symmetry Factor
1	**11.325**	18650197	636380	**95.160**	0.817
2	**25.342**	948603	18462	**4.840**	1.000

Figure S55. HPLC Profile for **3a** from liquor mothers, er 95:5.

***tert*-Butyl (*S*)-(4-(2,4-dioxopentan-3-yl)-3-ethyl-5-oxo-1-phenyl-4,5-dihydro-1*H*-pyrazol-4-yl)carbamate (3b).**

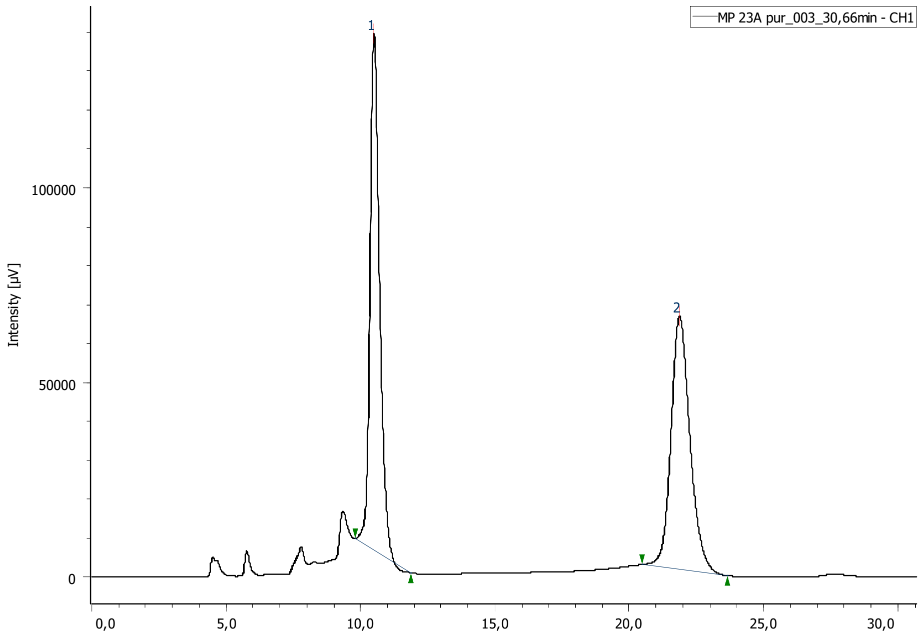

Peak Number	t_R(min)	Area	Height	Area (%)	Symmetry Factor
1	**10.492**	3268688	132530	**50.530**	1.275
2	**21.850**	3200091	64980	**49.470**	1.175

Figure S56. HPLC profile for **3b** (racemic).

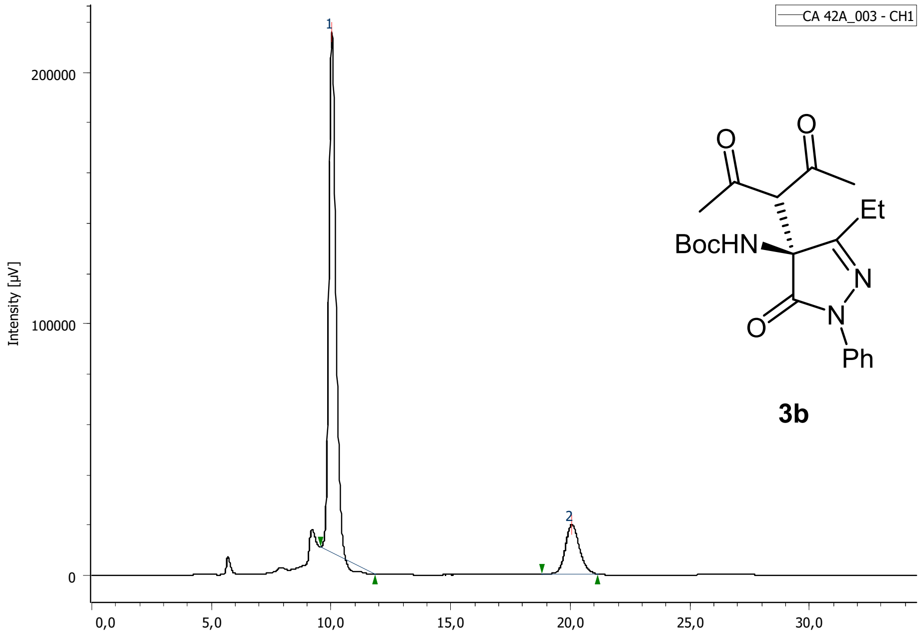

PeakNumber	t_R(min)	Area	Height	Area (%)	SymmetryFactor
1	**10.025**	4156356	206525	**83.853**	1.260
2	**20.033**	800333	19632	**16.147**	1.159

Figure S57. HPLC Profile for **3b** compound. Scheme 2, er 84:16.

***tert*-Butyl (*S*)-(4-(2,4-dioxopentan-3-yl)-3-isopropyl-5-oxo-1-phenyl-4,5-dihydro-1*H*-pyrazol-4-yl)carbamate (3c).**

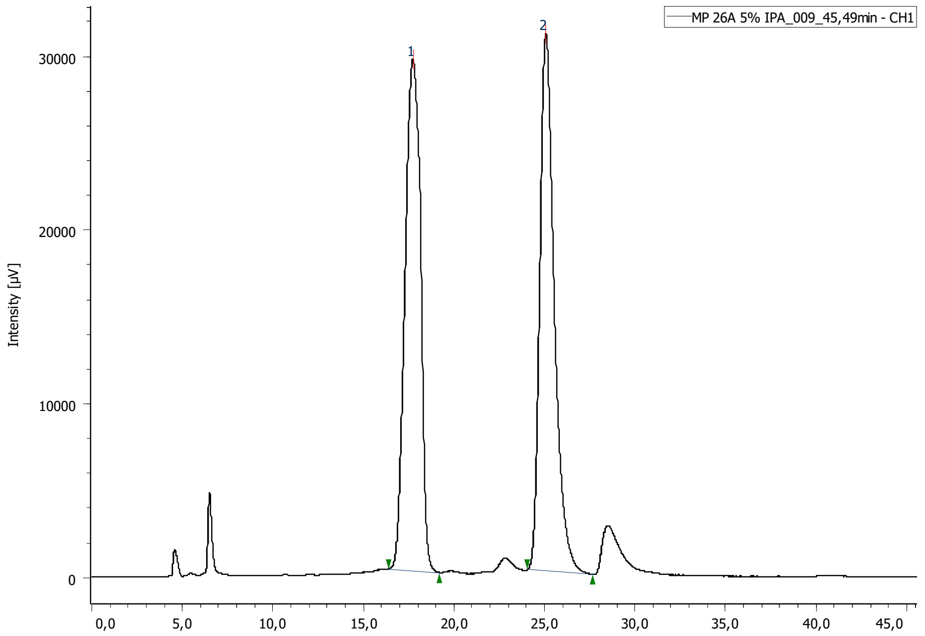

Peak Number	t_R(min)	Area	Height	Area (%)	Symmetry Factor
1	**17.708**	1750023	29389	**50.474**	0.953
2	**25.042**	1717121	30883	**49.526**	1.401

Figure S58. HPLC profile for **3c** (racemic).

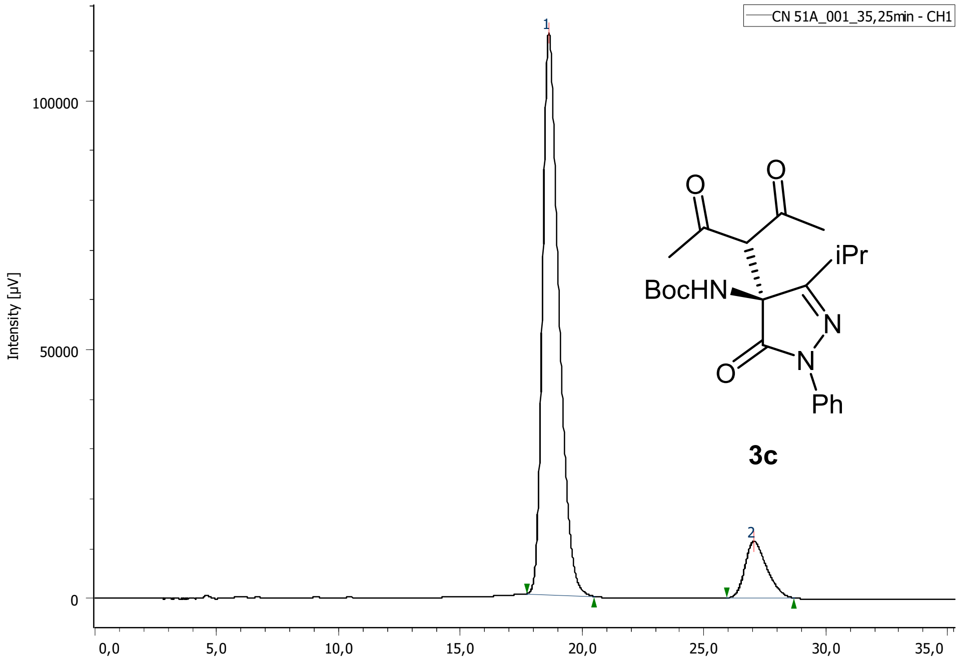

Peak Number	tR	Area	Height	Area%	Height%	Symmetry Factor
1	**18,600**	5241997	112514	**88,074**	90,758	1,437
2	**27,017**	709806	11458	**11,926**	9,242	1,290

Figure S59. HPLC Profile for **3c** compound. Scheme 2, er 88:12.

tert-Butyl (_S_)-(4-(2,4-dioxopentan-3-yl)-5-oxo-1,3-diphenyl-4,5-dihydro-1_H_-pyrazol-4-yl)carbamate (3e).

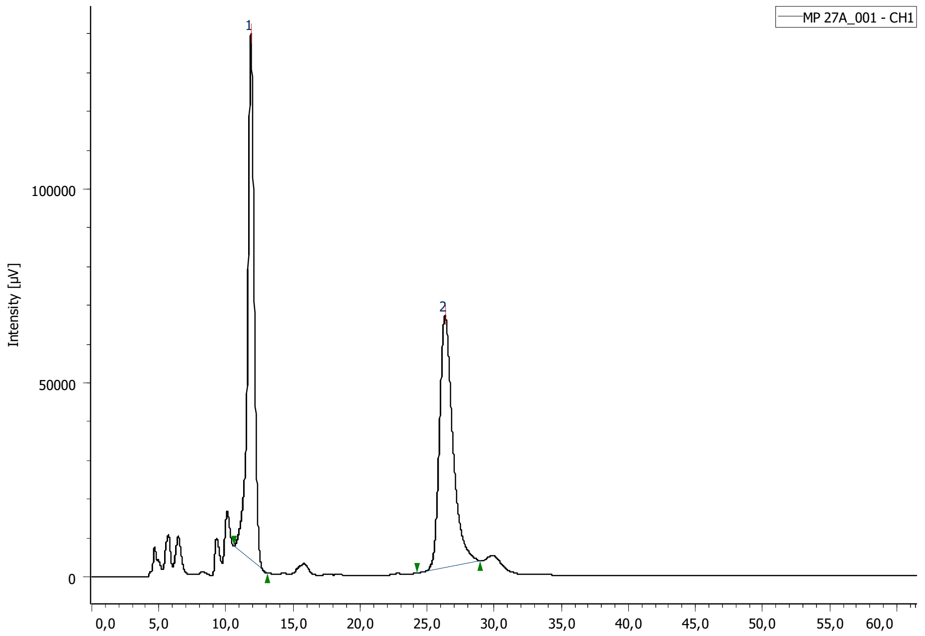

Peak Number	t_R(min)	Area	Height	Area (%)	Symmetry Factor
1	**11.842**	4548326	135340	**50.294**	0.868
2	**26.292**	4495142	64882	**49.706**	1.450

Figure S60. HPLC profile for **3e** (racemic).

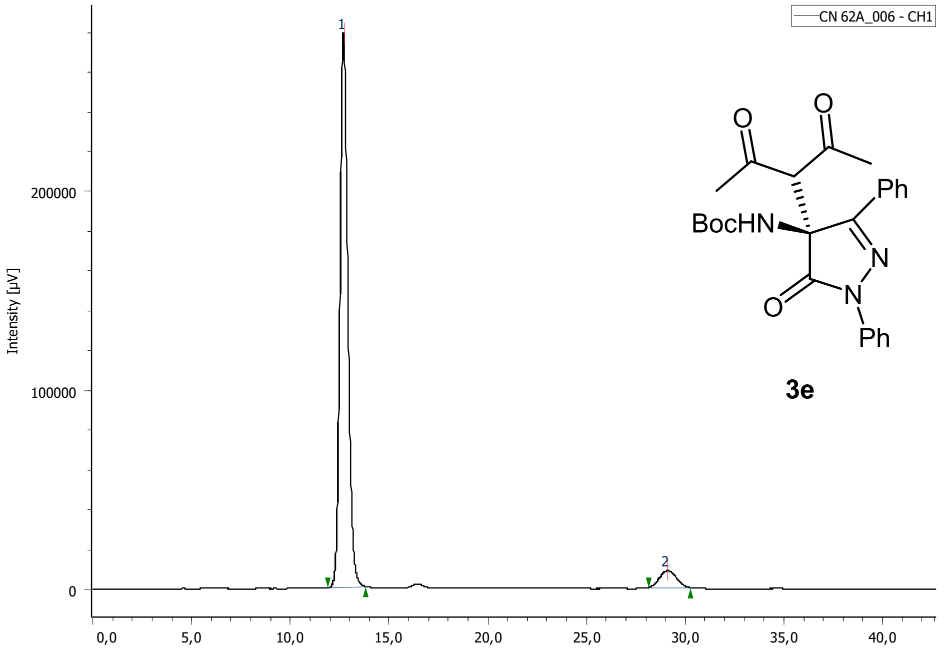

PeakNumber	t_R(min)	Area	Height	Area (%)	SymmetryFactor
1	**12,683**	7750572	278161	**93,900**	97,052
2	**29,083**	503455	8450	**6,100**	2,948

Figure S61. HPLC Profile for **3e** compound. Scheme 2, er 94:6.

***tert*-Butyl (*S*)-(1-(4-Chlorophenyl)-4-(2,4-dioxopentan-3-yl)-3-methyl-5-oxo-4,5-dihydro-1*H*-pyrazol-4-yl)carbamate (3f).**

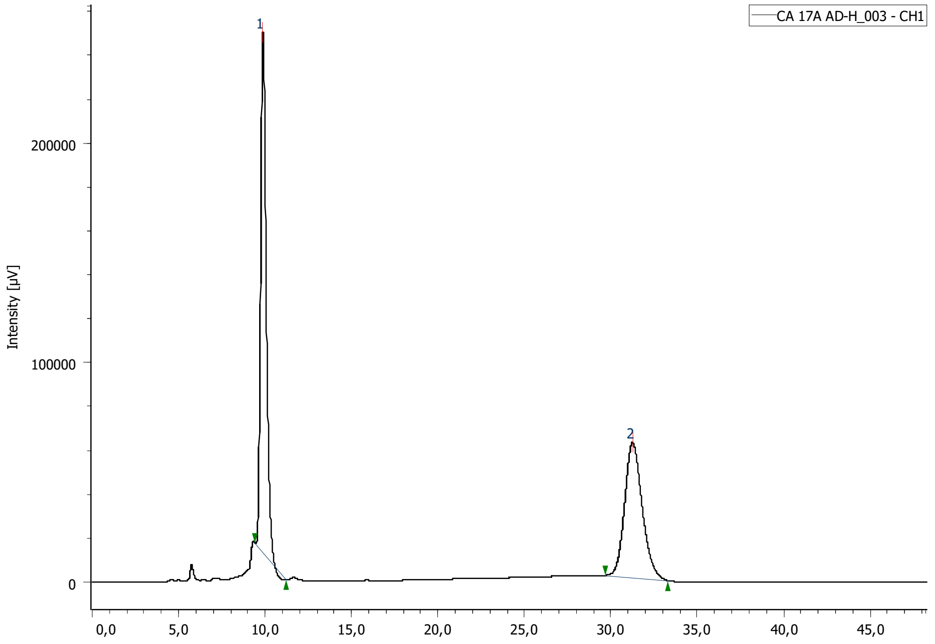

Peak Number	t_R(min)	Area	Height	Area (%)	Symmetry Factor
1	**9.867**	5291635	236518	**54.969**	1.361
2	**31.225**	4335008	61417	**45.031**	1.171

Figure S62. HPLC profile for **3f** (racemic).

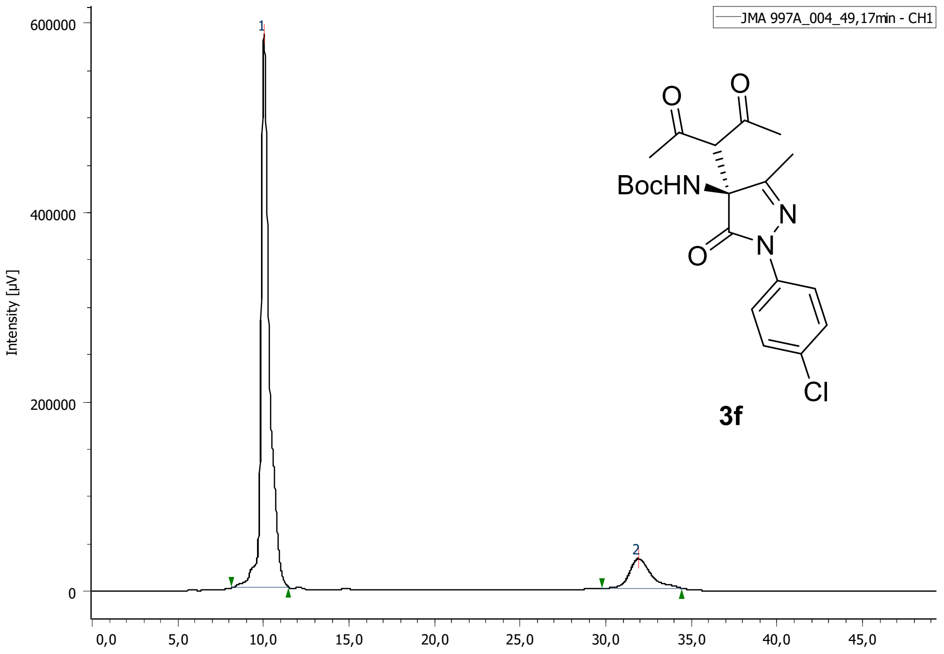

Peak Number	t_R(min)	Area	Height	Area (%)	Symmetry Factor
1	**10.017**	19795438	583412	**87.847**	1.624
2	**31.858**	2738504	31178	**12.153**	1.341

Figure S63. HPLC Profile for **3f** compound. Scheme 2, er 88:12.

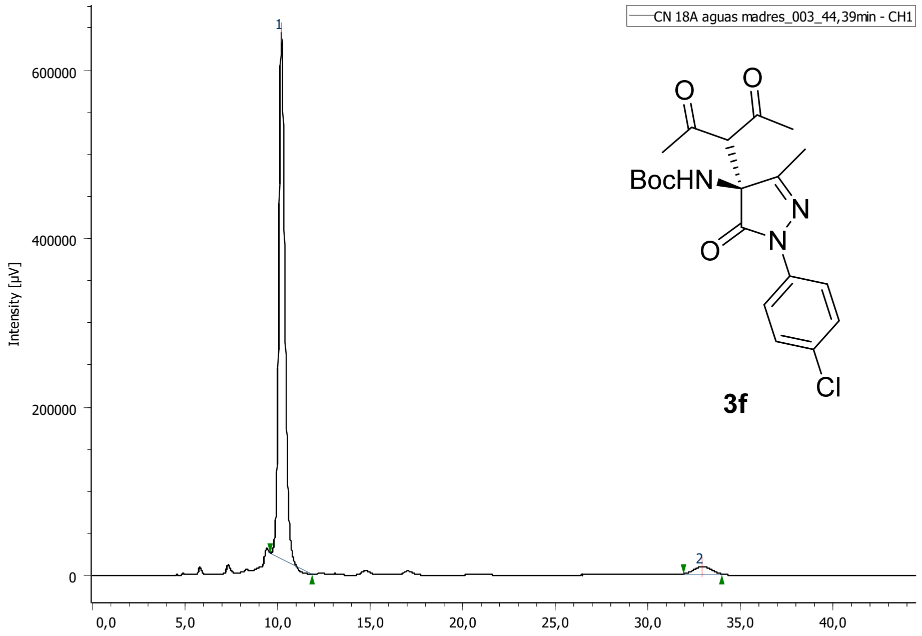

Peak Number	t_R(min)	Area	Height	Area (%)	Symmetry Factor
1	**10.200**	14221584	623717	**96.180**	1.191
2	**32.892**	564856	9087	**3.820**	1.044

Figure S64. HPLC Profile for **3f** from liquor mothers, er 96:4.

***tert*-Butyl (*S*)-(4-(2,4-dioxopentan-3-yl)-3-methyl-5-oxo-1-(*p*-tolyl)-4,5-dihydro-1*H*-pyrazol-4-yl)carbamate (3g).**

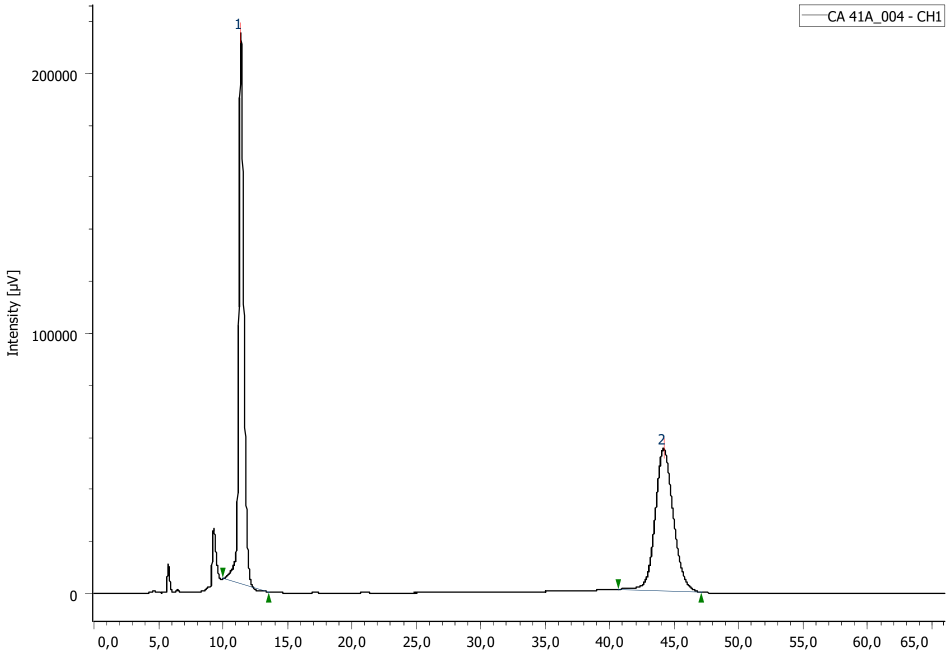

Peak Number	t_R(min)	Area	Height	Area (%)	Symmetry Factor
1	**11,383**	5657641	211401	**51,305**	79,505
2	**44,117**	5369868	54496	**48,695**	20,495

Figure S65. HPLC profile for **3g** (racemic).

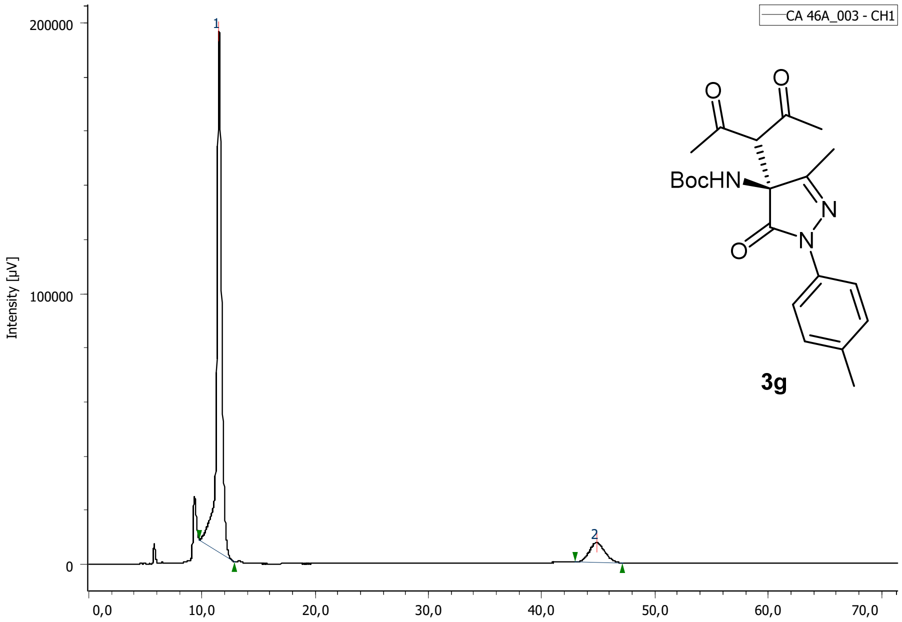

Peak Number	t_R(min)	Area	Height	Area (%)	Symmetry Factor
1	**11.492**	5695956	191925	**89.075**	0.839
2	**44.775**	698588	7276	**10.925**	1.120

Figure S66. HPLC Profile for **3g** compound. Scheme 2, er 89:11.

***tert*-Butyl (*S*)-(4-(3,5-dioxoheptan-4-yl)-3-methyl-5-oxo-1-phenyl-4,5-dihydro-1*H*-pyrazol-4-yl)carbamate (3h).**

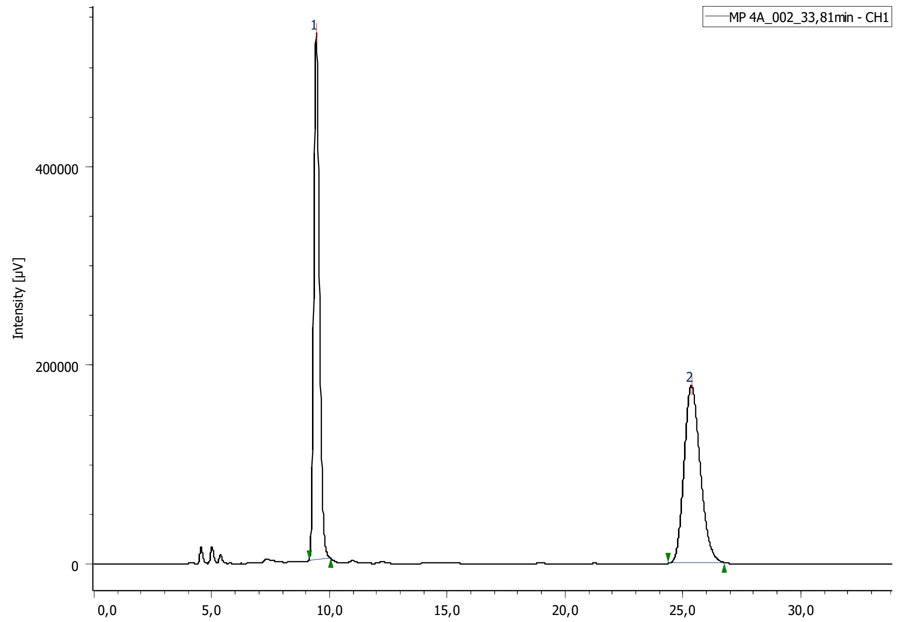

Peak Number	t_R(min)	Area	Height	Area (%)	Symmetry Factor
1	**9.425**	8614606	529030	**50.208**	1.248
2	**25.325**	8543387	178745	**49.792**	1.197

Figure S67. HPLC profile for **3h** (racemic).

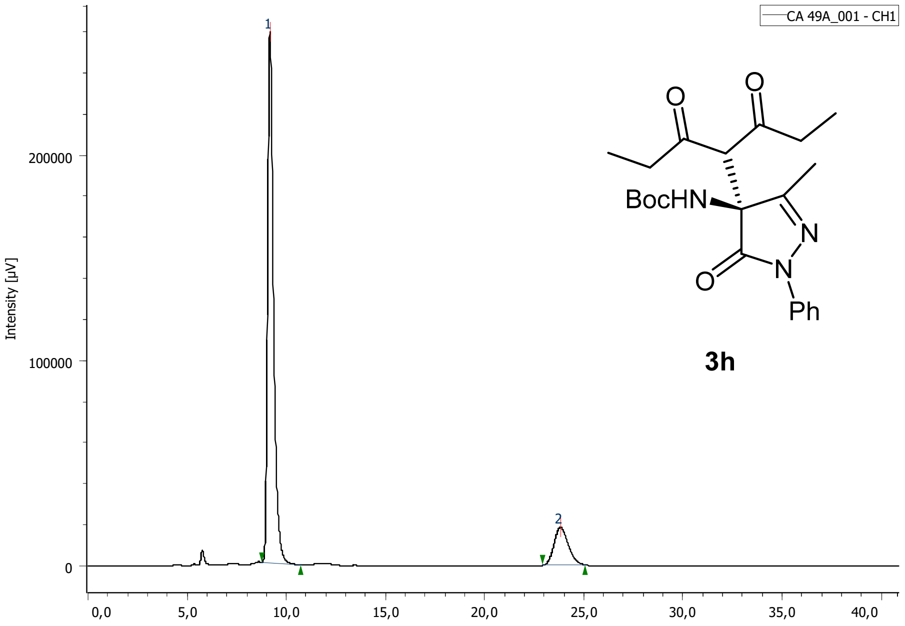

3h

Peak Number	t_R(min)	Area	Height	Area (%)	Symmetry Factor
1	**9.167**	5289670	257882	**85.194**	1.507
2	**23.792**	919285	18509	**14.806**	1.196

Figure S68. HPLC Profile for **3h** compound. Scheme 2, er 85:15.

***tert*-Butyl (*S*)-(4-(1,3-dioxo-1,3-diphenylpropan-2-yl)-3-methyl-5-oxo-1-phenyl-4,5-dihydro-1*H*-pyrazol-4-yl)carbamate (3i).**

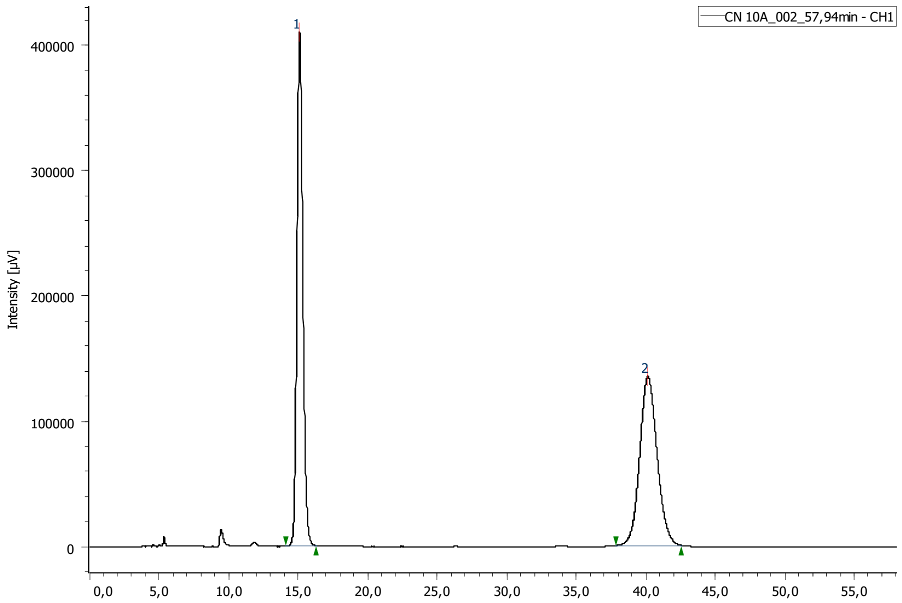

Peak Number	t_R(min)	Area	Height	Area (%)	Symmetry Factor
1	**15.075**	11929079	408849	**50.462**	1.166
2	**40.100**	11710796	134520	**49.538**	1.099

Figure S69. HPLC profile for **3i** (racemic).

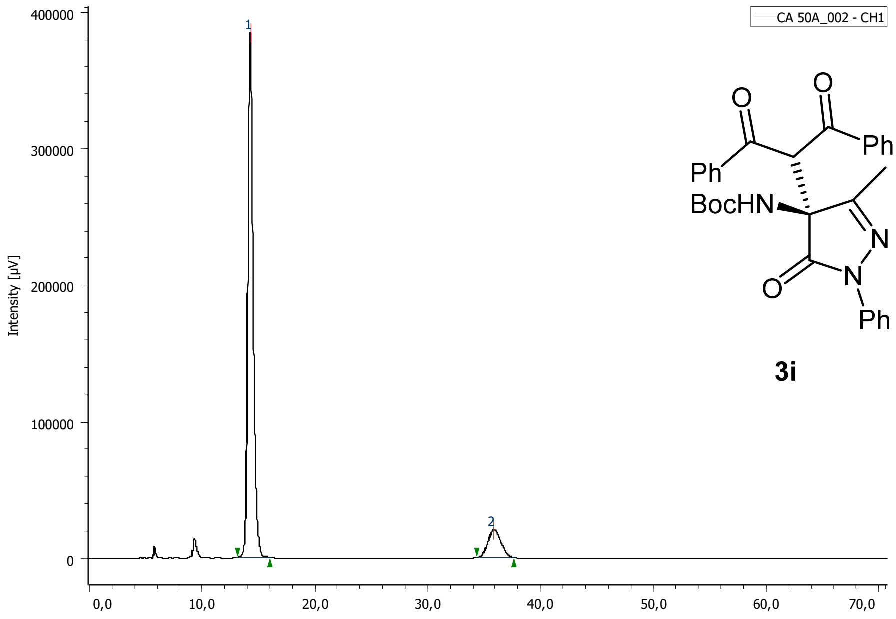

Peak Number	t_R(min)	Area	Height	Area (%)	Symmetry Factor
1	**14.242**	12504213	383289	**88.315**	1.271
2	**35.833**	1654430	20551	**11.685**	1.106

Figure S70. HPLC Profile for **3i** compound. Scheme 2, er 88:12.

***tert*-Butyl (*S*)-(4-(3,5-dioxoheptan-4-yl)-5-oxo-1,3-diphenyl-4,5-dihydro-1*H*-pyrazol-4-yl)carbamate (3j).**

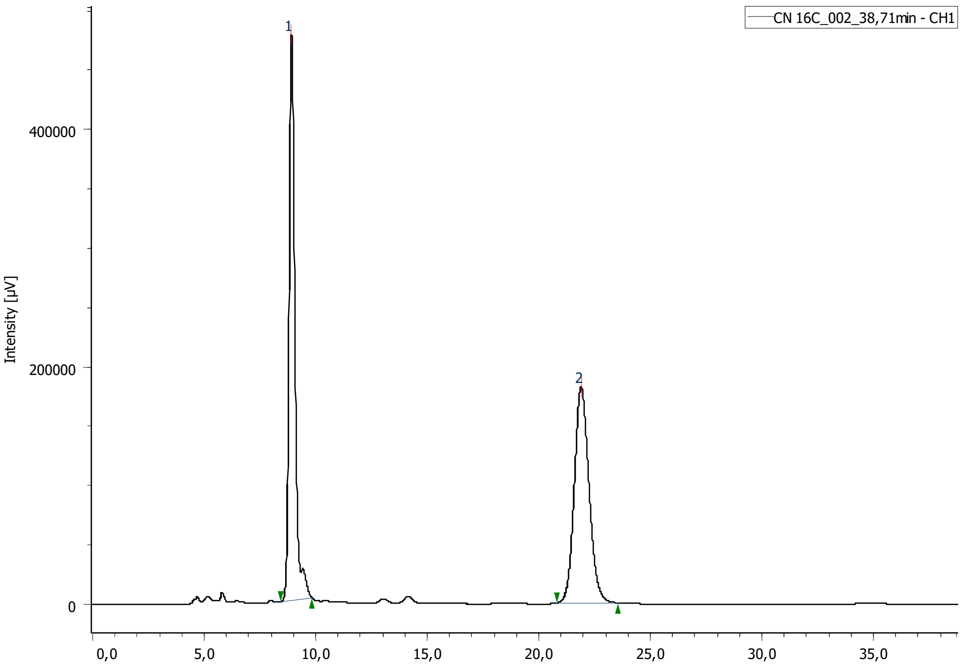

Peak Number	tR	Area	Height	Area%	Height%	Symmetry Factor
1	**8,908**	8625302	476025	**50,756**	72,324	1,599
2	**21,867**	8368363	182162	**49,244**	27,676	1,118

Figure S71. HPLC profile for **3j** (racemic).

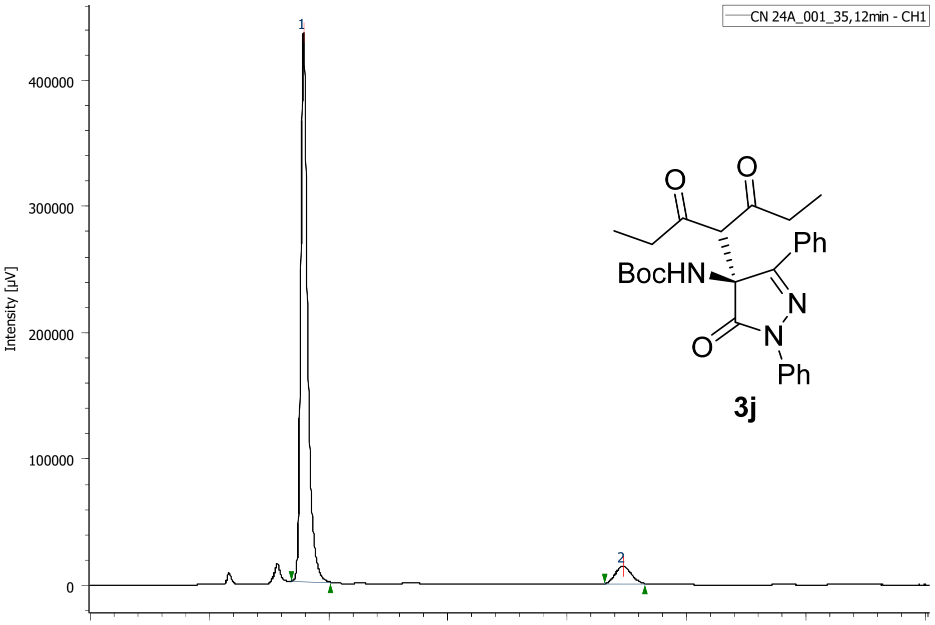

3j

Peak Number	tR	Area	Height	Area%	Height%	Symmetry Factor
1	**8,933**	8714793	433523	**93,270**	96,913	1,473
2	**22,308**	628810	13809	**6,730**	3,087	1,093

Figure S72. HPLC Profile for **3j** compound. Scheme 2, er 93:7.

***tert*-Butyl (*S*)-(4-(1,3-dioxo-1,3-diphenylpropan-2-yl)-5-oxo-1,3-diphenyl-4,5-dihydro-1*H*-pyrazol-4-yl)carbamate (3k).**

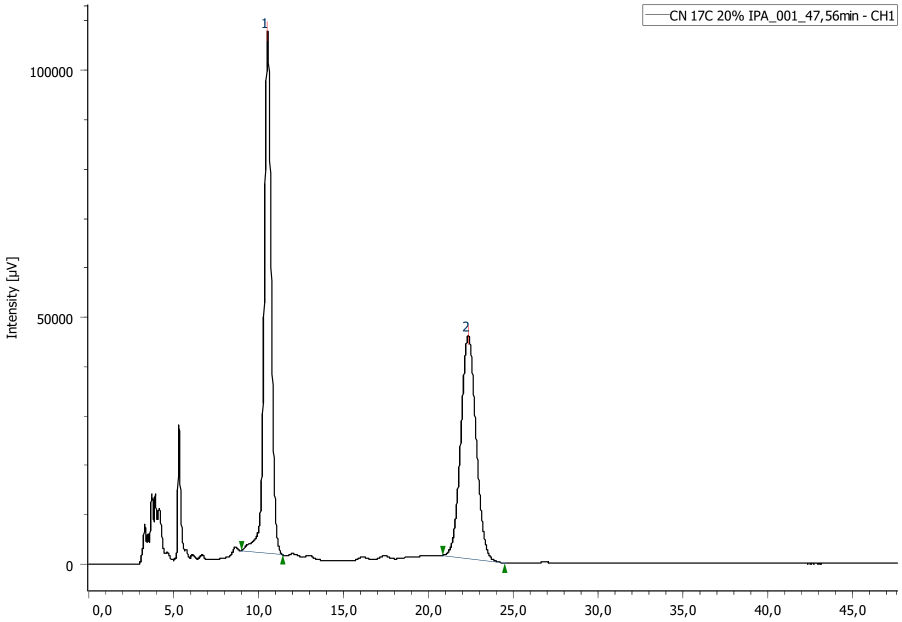

Peak Number	tR	Area	Height	Area%	Height%	Symmetry Factor
1	**10,517**	3074291	105439	**52,217**	70,114	1,020
2	**22,317**	2813199	44943	**47,783**	29,886	1,060

Figure S73. HPLC profile for **3k** (racemic).

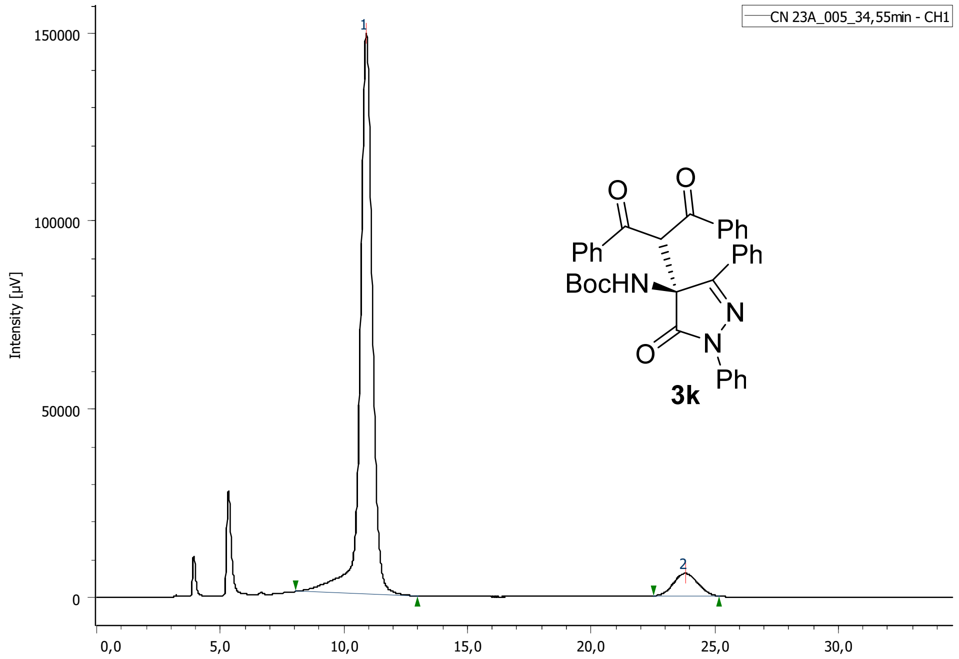

Peak Number	tR	Area	Height	Area%	Height%	Symmetry Factor
1	**10,892**	5164528	148763	**92,597**	96,113	1,004
2	**23,792**	412913	6017	**7,403**	3,887	1,032

Figure S74. HPLC Profile for **3k** compound. Scheme 2, er 93:7.

***tert*-Butyl (*S*)-(4-(2,4-dioxopentan-3-yl)-1-methyl-5-oxo-3-phenyl-4,5-dihydro-1*H*-pyrazol-4-yl)carbamate (3l).**

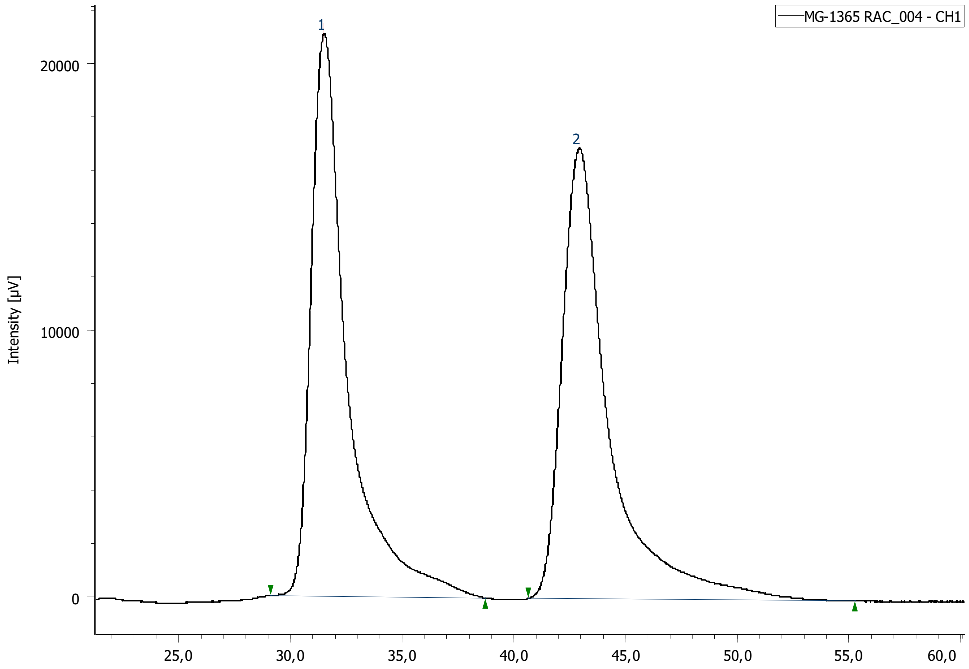

Peak Number	t_R(min)	Area	Height	Area (%)	Symmetry Factor
1	**31,500**	2377916	21054	**49,268**	2,110
2	**42,900**	2448559	16867	**50,732**	2,115

Figure S75. HPLC profile for **3l** (racemic).

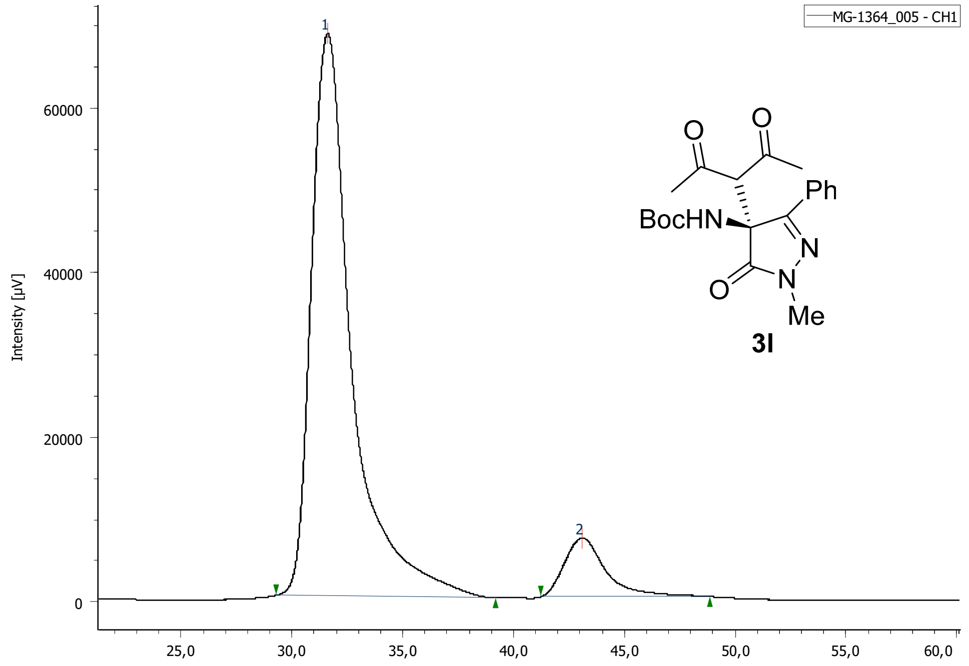

Peak Number	t_R(min)	Area	Height	Area (%)	Symmetry Factor
1	**31,608**	8642626	68202	**90,288**	1,851
2	**43,067**	929657	7151	**9,712**	1,601

Figure S76. HPLC Profile for **3l** compound. Scheme 2, er 90:10.

***tert*-Butyl (*S*)-(3,3',5-trimethyl-5'-oxo-1'-phenyl-1',5'-dihydro-1*H*,4'*H*-[4,4'-bipyrazol]-4'-yl)carbamate (4a).**

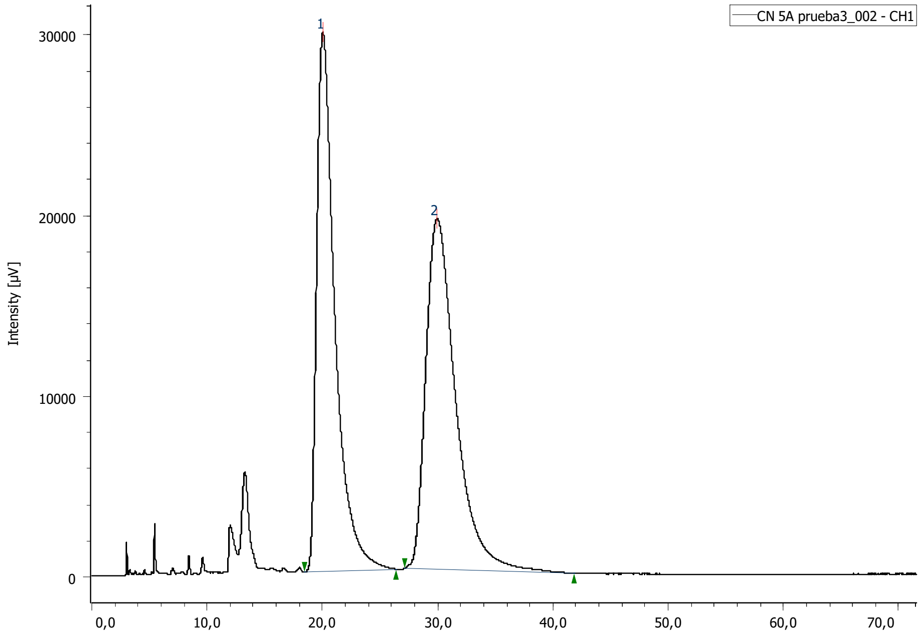

Peak Number	t_R(min)	Area	Height	Area (%)	Symmetry Factor
1	**20.017**	3270382	29798	**48.354**	2.003
2	**29.900**	3493034	19375	**51.646**	1.664

Figure S77. HPLC profile for **4a** (racemic).

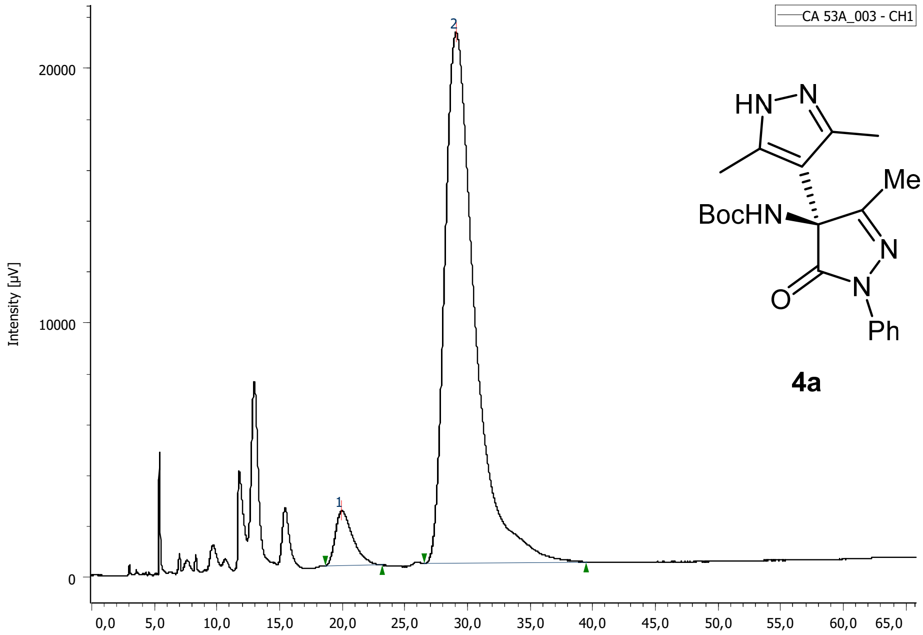

Peak Number	t_R(min)	Area	Height	Area (%)	Symmetry Factor
1	**19.950**	211642	2157	**5.740**	1.597
2	**29.050**	3475661	20826	**94.260**	1.852

Figure S78. HPLC Profile for **4a** compound. Scheme 3, er 94:6.

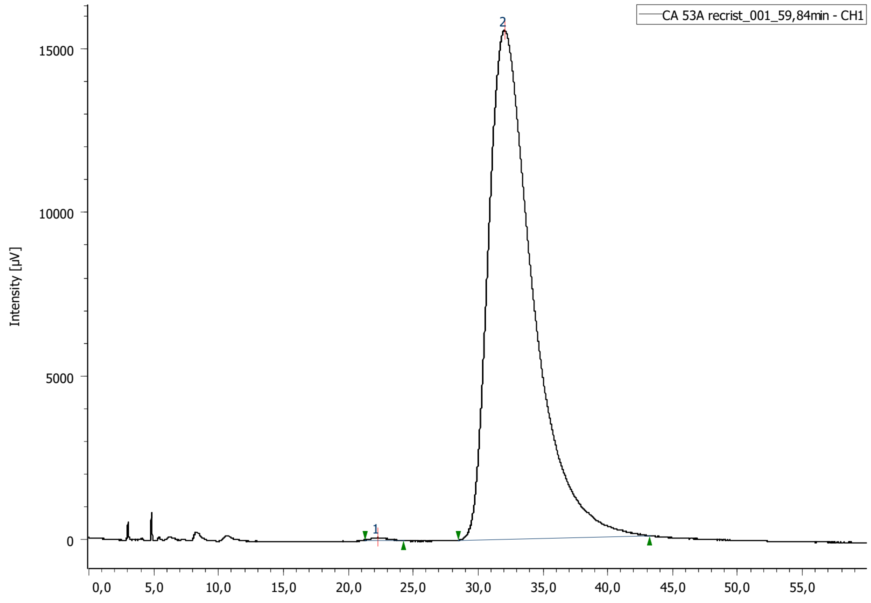

PeakNumber	t_R(min)	Area	Height (µV)	Area (%)	SymmetryFactor
1	**22,233**	6459	81	**0,175**	0,521
2	**32,008**	3693654	15551	**99,825**	99,479

Figure S79. HPLC Profile for **4a** after recrystallization. er ≥ 99:1.

***tert*-Butyl (*S*)-(3'-ethyl-3,5-dimethyl-5'-oxo-1'-phenyl-1',5'-dihydro-1*H*,4'*H*-[4,4'-bipyrazol]-4'-yl)carbamate (4b).**

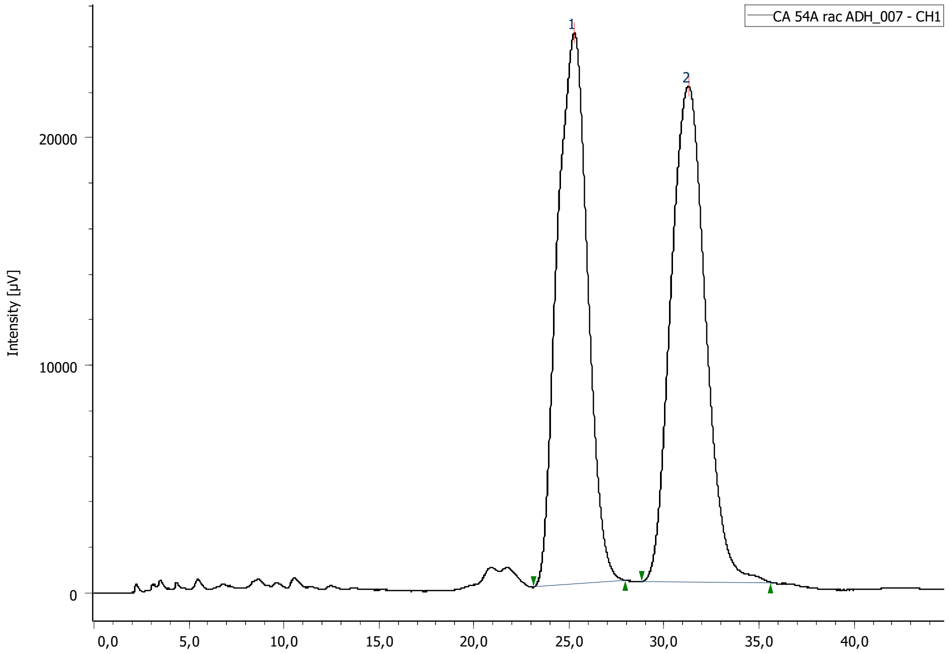

Peak Number	tR	Area	Height	Area%	Height%	Symmetry Factor
1	**25,242**	2685984	24175	**49,291**	52,631	1,011
2	**31,242**	2763309	21758	**50,709**	47,369	1,098

Figure S80. HPLC profile for **4b** (racemic).

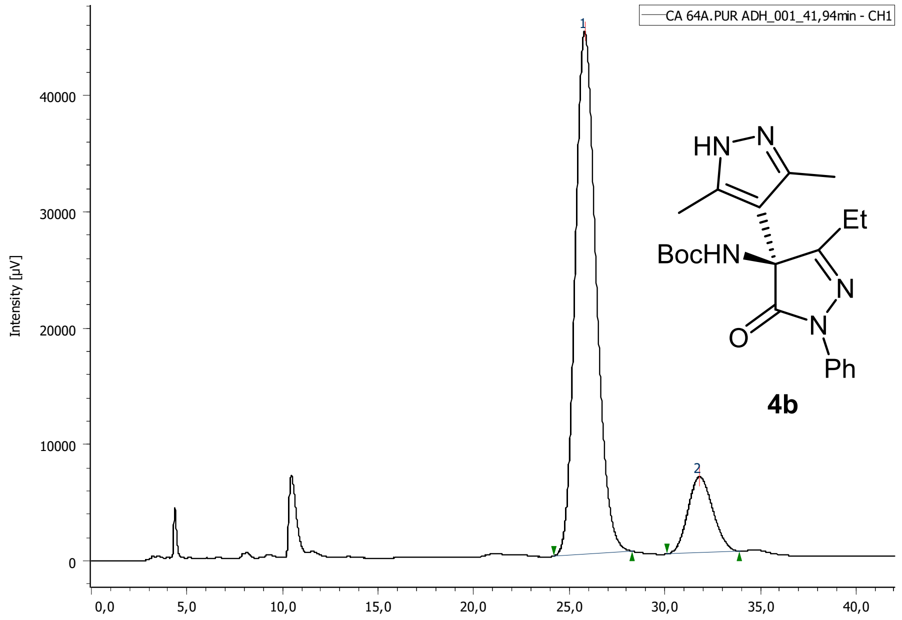

Peak Number	tR	Area	Height	Area%	Height%	Symmetry Factor
1	**25,767**	3406140	44845	**85,215**	87,294	1,200
2	**31,767**	590969	6528	**14,785**	12,706	1,122

Figure S81. HPLC Profile for **4b** compound. Scheme 3, er 85:15.

***tert*-Butyl (*S*)-(3'-isopropyl-3,5-dimethyl-5'-oxo-1'-phenyl-1',5'-dihydro-1*H*,4'*H*-[4,4'-bipyrazol]-4'-yl)carbamate (4c).**

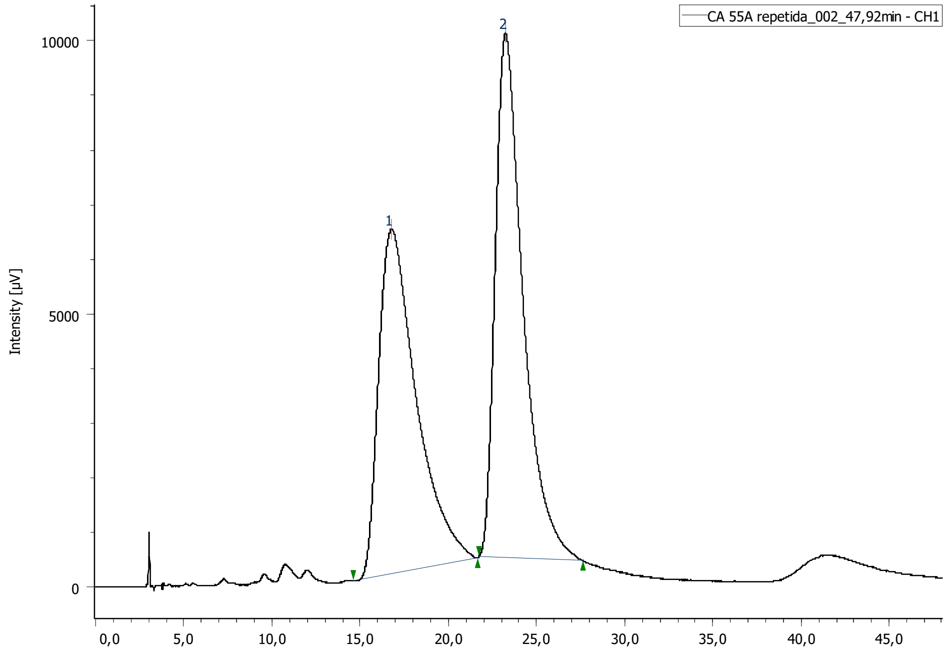

Peak Name	tR	Area	Height	Area%	Height%	Symmetry Factor
1	**16,775**	934391	6310	**48,848**	40,085	1,837
2	**23,200**	978461	9432	**51,152**	59,915	1,588

Figure S82. HPLC profile for **4c** (racemic).

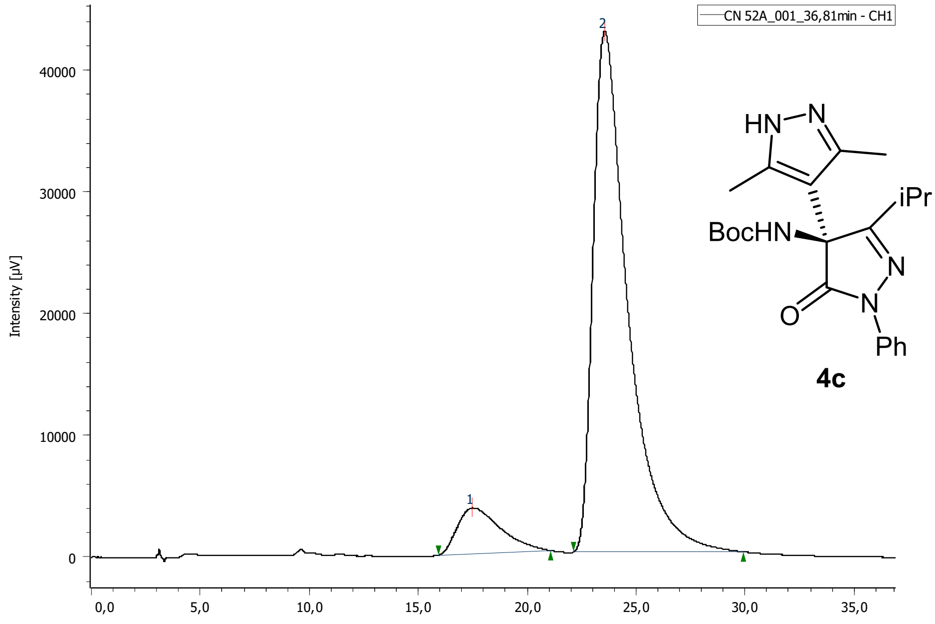

Peak Number	tR	Area	Height	Area%	Height%	Symmetry Factor
1	**17,475**	515449	3760	**9,790**	8,094	1,660
2	**23,533**	4749472	42690	**90,210**	91,906	1,998

Figure S83. HPLC Profile for **4c** compound. Scheme 3, er 90:10.

tert-Butyl (S)-(3,5-dimethyl-5'-oxo-1',3'-diphenyl-1',5'-dihydro-1H,4'H-[4,4'-bipyrazol]-4'-yl)carbamate (4e).

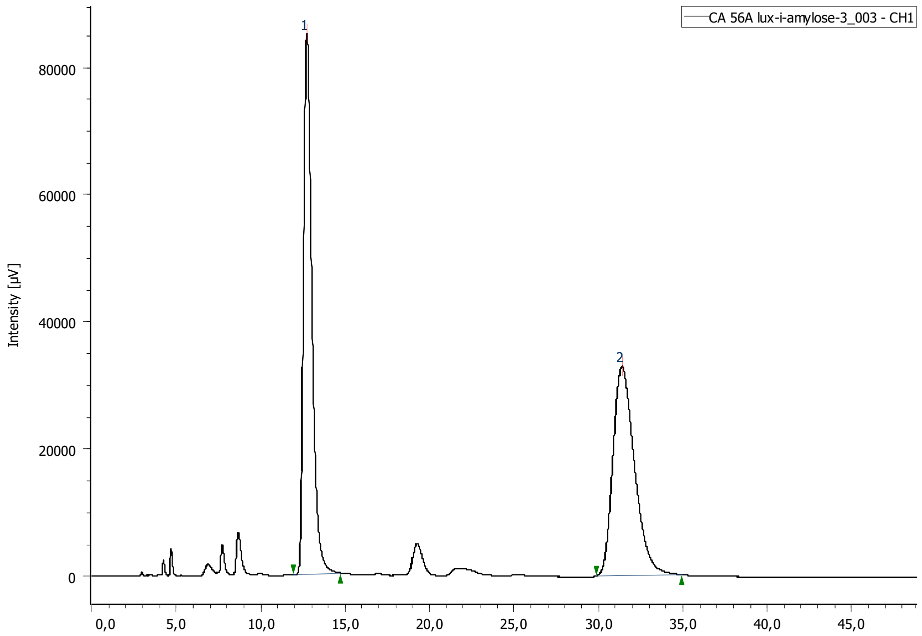

Peak Number	t_R(min)	Area	Height	Area (%)	Symmetry Factor
1	**12.717**	2931594	84826	**50.086**	1.487
2	**31.350**	2921501	32808	**49.914**	1.364

Figure S84. HPLC profile for **4e** (racemic).

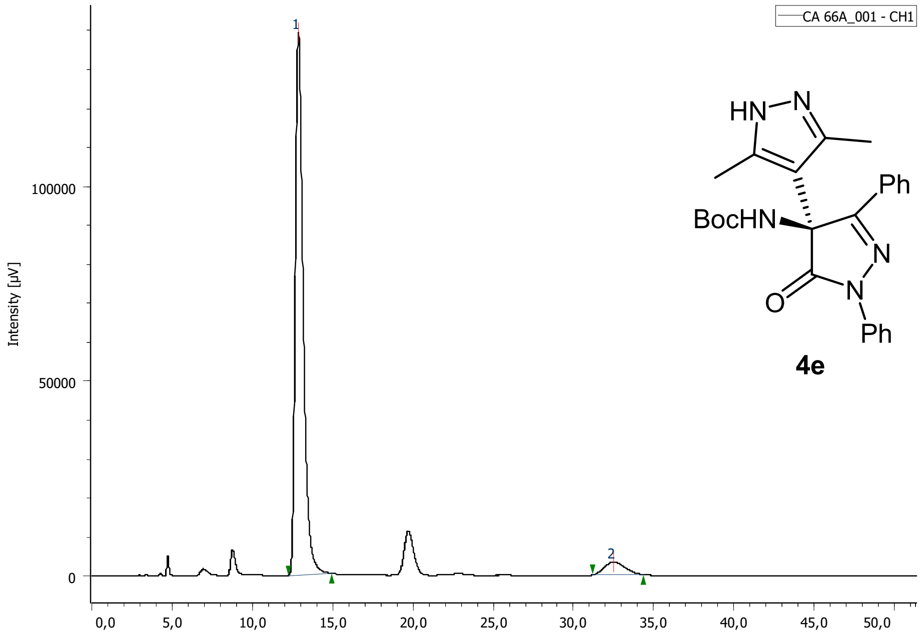

Peak Number	t_R(min)	Area	Height	Area (%)	Symmetry Factor
1	**12.850**	4772695	138915	**94.217**	1.496
2	**32.458**	292947	3283	**5.783**	1.184

Figure S85. HPLC Profile for **4e** compound. Scheme 3, er 94:6.

***tert*-Butyl (*S*)-(1'-(4-chlorophenyl)-3,3',5-trimethyl-5'-oxo-1',5'-dihydro-1*H*,4'*H*-[4,4'-bipyrazol]-4'-yl)carbamate (4f).**

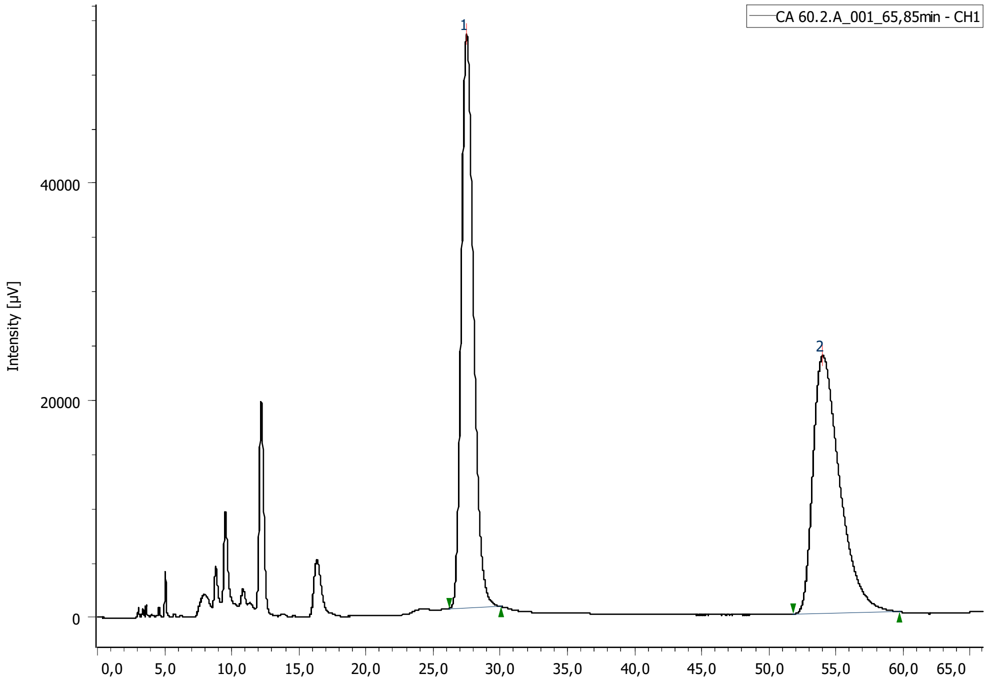

Peak Number	t_R(min)	Area	Height	Area (%)	Symmetry Factor
1	**27,467**	3337320	52765	**50,307**	1,272
2	**53,942**	3296547	23760	**49,693**	1,652

Figure S86. HPLC profile for **4f** (racemic).

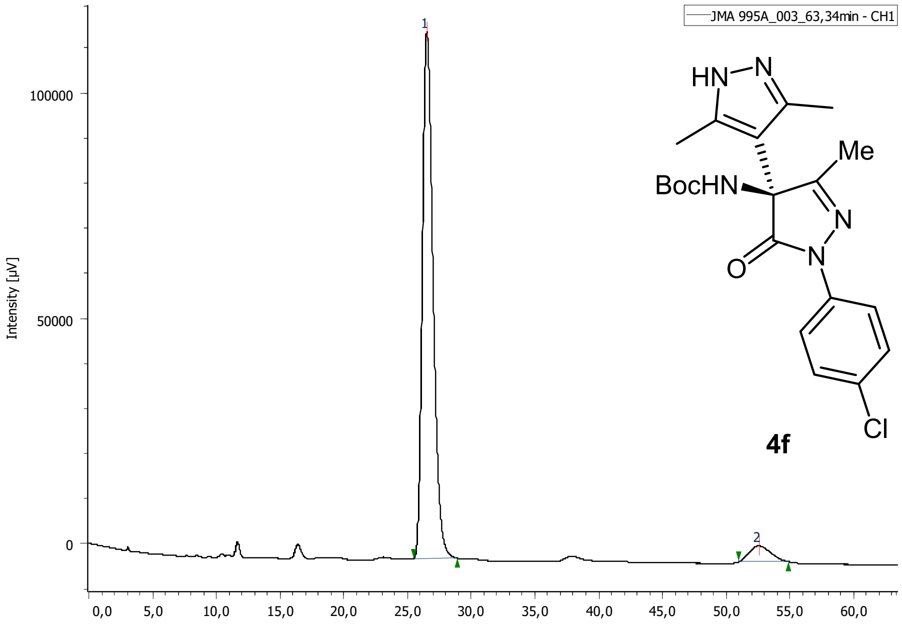

Peak Number	t_R(min)	Area	Height	Area (%)	Symmetry Factor
1	**26,483**	6760936	116665	**94,340**	1,327
2	**52,483**	405643	3514	**5,660**	1,230

Figure S87. HPLC Profile for **4f** compound. Scheme 3, er 94:6.

***tert*-Butyl (*S*)-(3,3',5-trimethyl-5'-oxo-1'-(*p*-tolyl)-1',5'-dihydro-1*H*,4'*H*-[4,4'-bipyrazol]-4'-yl)carbamate (4g).**

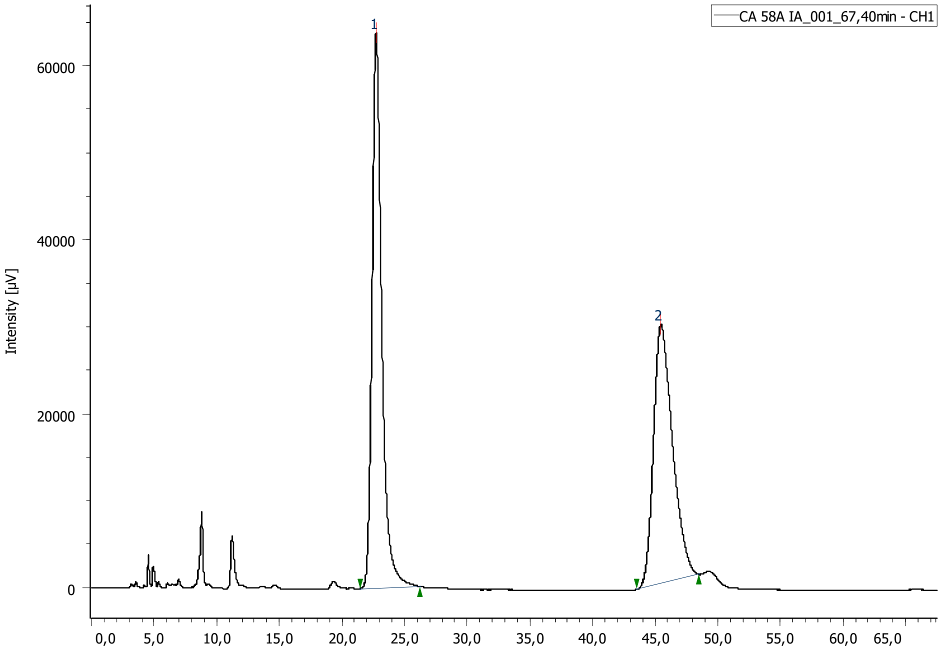

Peak Number	tR	Area	Height	Area%	Height%	Symmetry Factor
1	**22,700**	3206770	62887	**50,486**	67,922	1,266
2	**45,425**	3145023	29700	**49,514**	32,078	1,360

Figure S88. HPLC profile for **4g** (racemic).

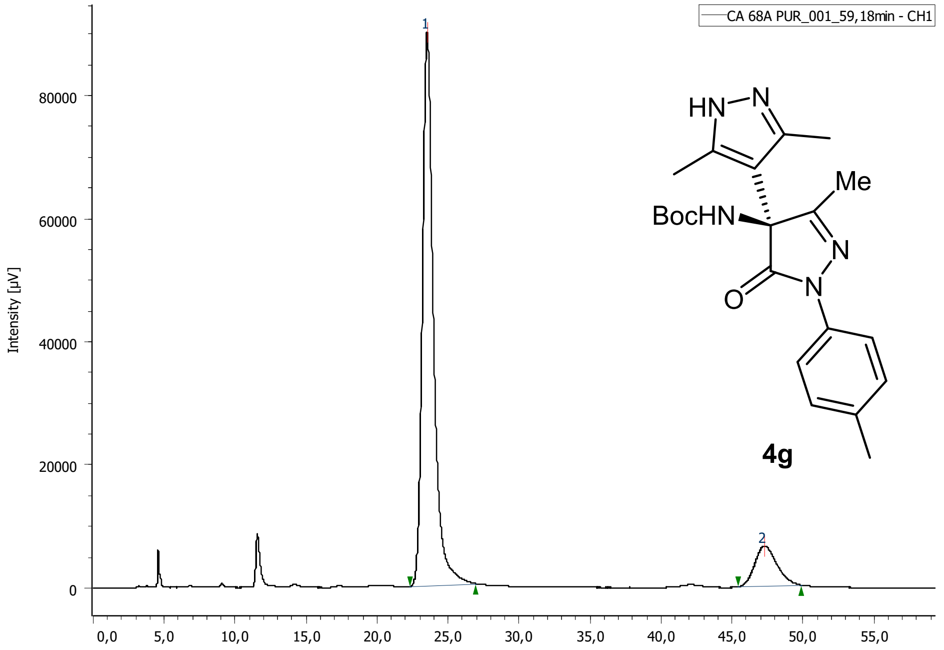

Peak Number	tR	Area	Height	Area%	Height%	Symmetry Factor
1	**23,492**	4908020	89812	**88,051**	93,426	1,382
2	**47,217**	666014	6319	**11,949**	6,574	1,289

Figure S89. HPLC Profile for **4g** compound. Scheme 3, er 88:12.

***tert*-Butyl (*S*)-(3,5-diethyl-5'-oxo-1',3'-diphenyl-1',5'-dihydro-1*H*,4'*H*-[4,4'-bipyrazol]-4'-yl)carbamate (4j).**

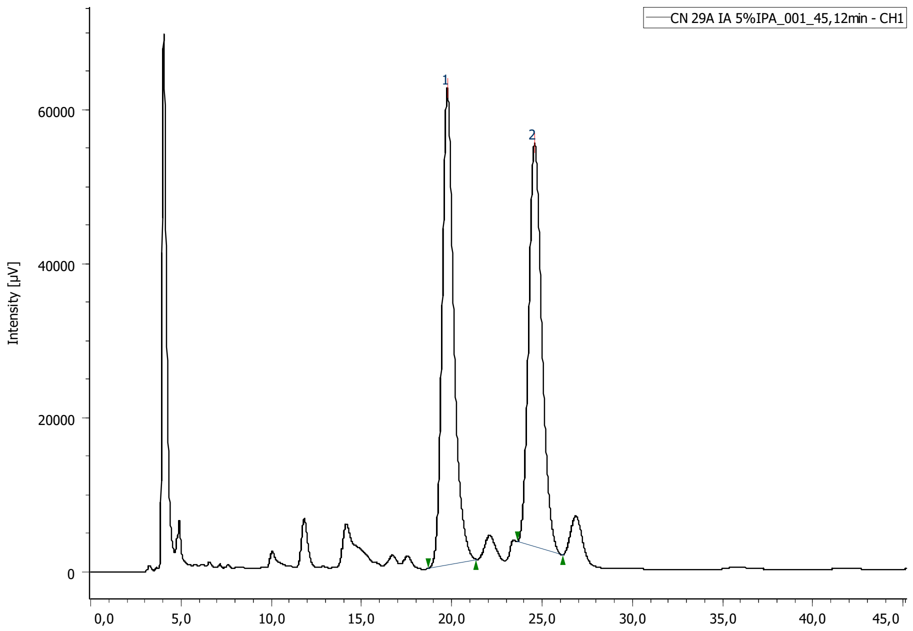

Peak Number	tR	Area	Height	Area%	Height%	Symmetry Factor
1	**19,725**	276801	61716	**51,125**	54,161	1,299
2	**24,550**	2646161	52232	**48,875**	45,839	1,205

Figure S90. HPLC profile for **4j** (racemic).

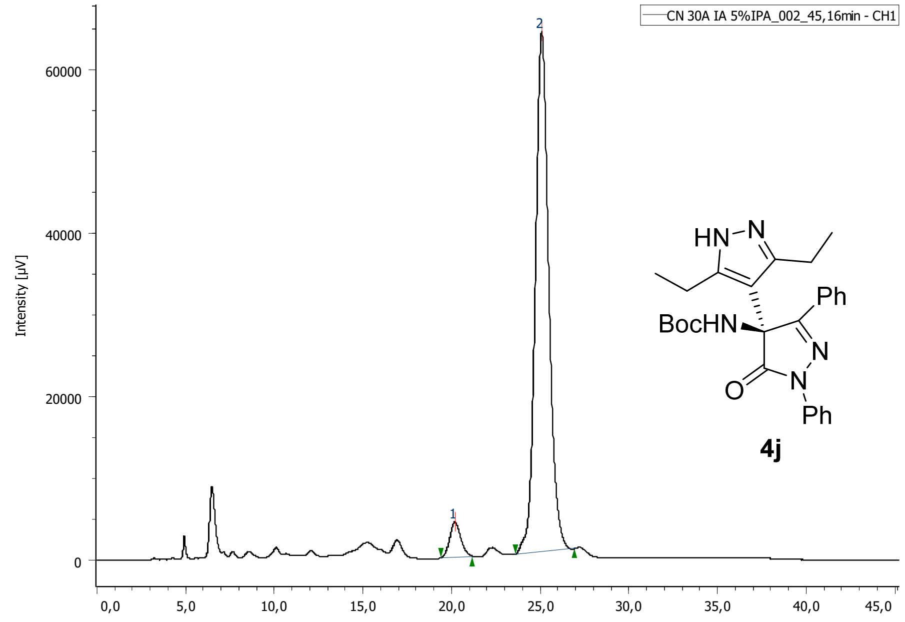

Peak Number	tR	Area	Height	Area%	Height%	Symmetry Factor
1	**20,158**	185714	4311	**5,273**	6,349	1,125
2	**25,042**	3336290	63586	**94,727**	93,651	1,118

Figure S91. HPLC Profile for **4j** compound. Scheme 3, er 95:5.

***tert*-Butyl (*S*)-(3-methyl-5-oxo-4-(2-oxo-2-phenylethyl)-1-phenyl-4,5-dihydro-1*H*-pyrazol-4-yl)carbamate (5i)**.

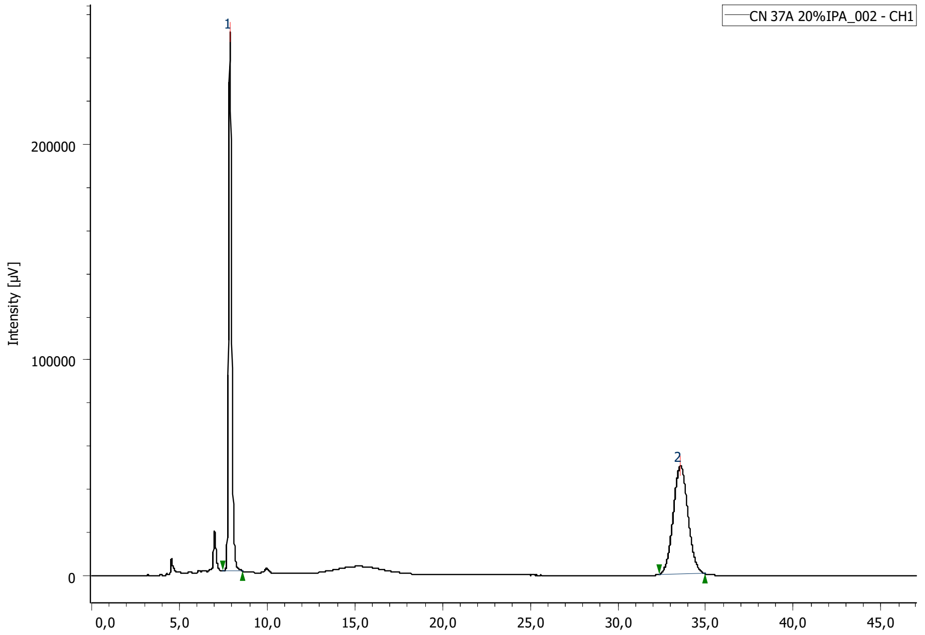

Peak Name	tR	Area	Height	Area%	Height%	Symmetry Factor
1	**7,875**	3002905	249516	**50,441**	83,214	1,226
2	**33,517**	2950438	50333	**49,559**	16,786	1,058

Figure S92. HPLC profile for **5i** (racemic).

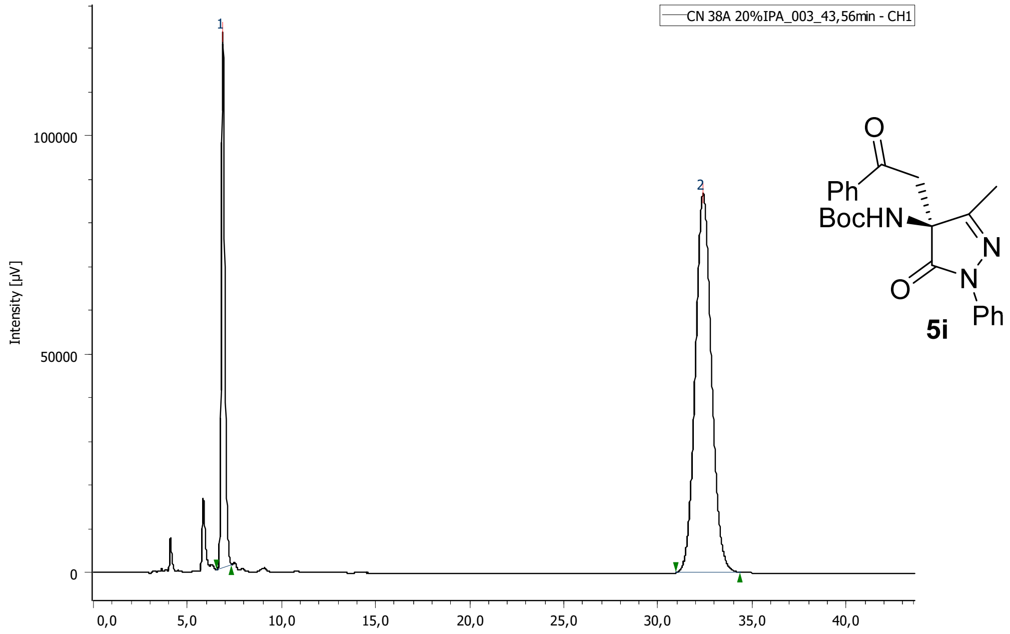

Peak Name	tR	Area	Height	Area%	Height%	Symmetry Factor
1	**6,875**	1551170	122385	**23,092**	58,562	1,260
2	**32,358**	5166215	86600	**76,908**	41,438	1,074

Figure S93. HPLC Profile for **5i** compound. Scheme 3, er 77:23.

***tert*-Butyl (*S*)-(1-(4-chlorophenyl)-3,3',5-trimethyl-5'-oxo-1'-phenyl-1',5'-dihydro-1*H*,4'*H*-[4,4'-bipyrazol]-4'-yl)carbamate (6a).**

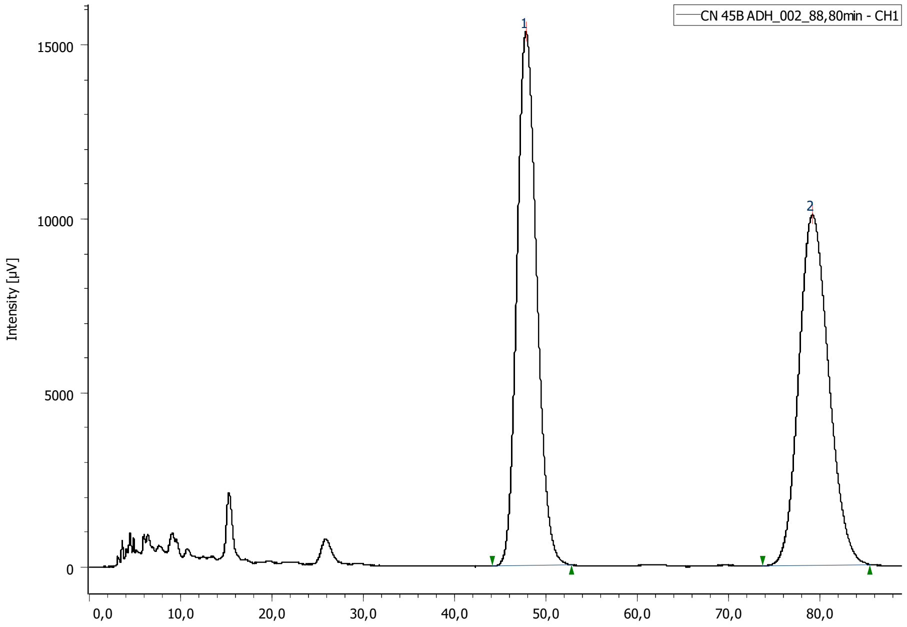

Peak Number	tR	Area	Height	Area%	Height%	Symmetry Factor
1	**47,758**	2305688	15319	**50,132**	60,338	1,173
2	**79,067**	2293573	10070	**49,868**	39,662	1,157

Figure S94. HPLC profile for **6a** (racemic).

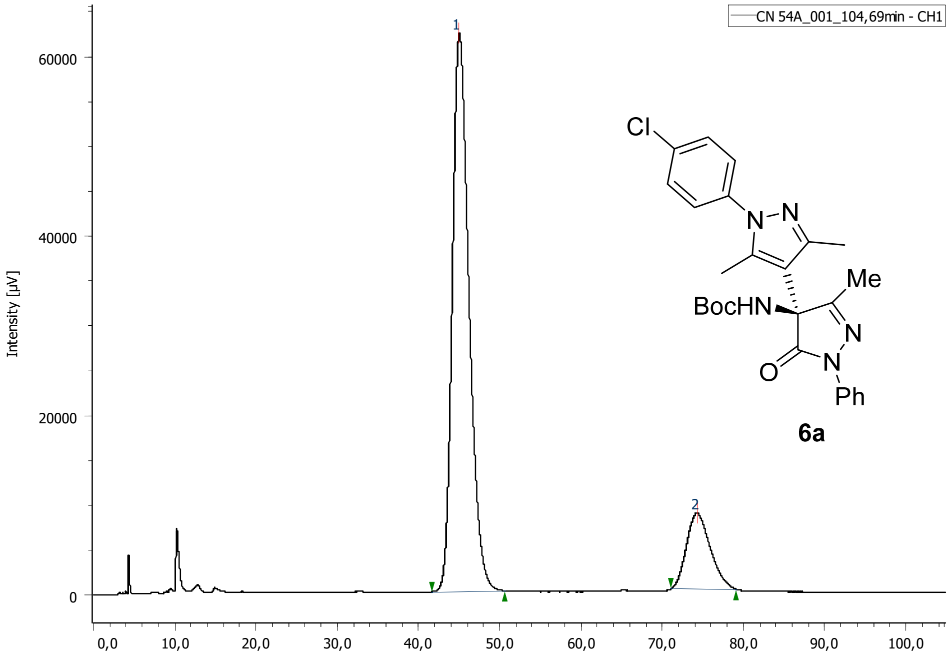

Peak Number	tR	Area	Height	Area%	Height%	Symmetry Factor
1	**44,967**	8828181	62101	**83,614**	88,009	1,296
2	**74,183**	1730125	8461	**16,386**	11,991	1,217

Figure S95. HPLC Profile for **6a** compound. Scheme 5, er 84:16.

tert-Butyl (S)-(1-(4-chlorophenyl)-3,5-dimethyl-5'-oxo-1',3'-diphenyl-1',5'-dihydro-1H,4'H-[4,4'-bipyrazol]-4'-yl)carbamate (6e).

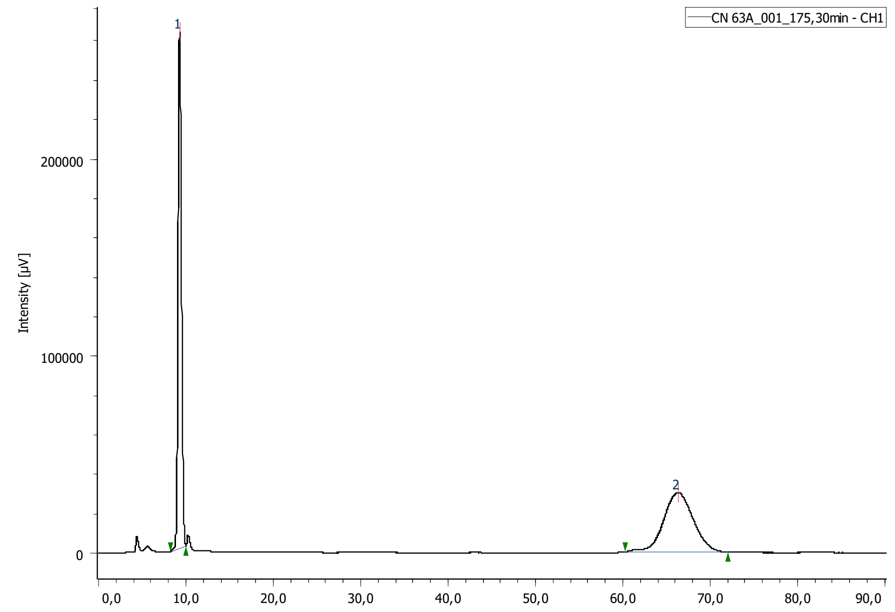

Peak Number	tR	Area	Height	Area%	Height%	Symmetry Factor
1	**9,275**	7170915	261657	**50,261**	89,668	1,120
2	**66,267**	7096443	30149	**49,739**	10,332	1,008

Figure S96. HPLC profile for **6e** (racemic).

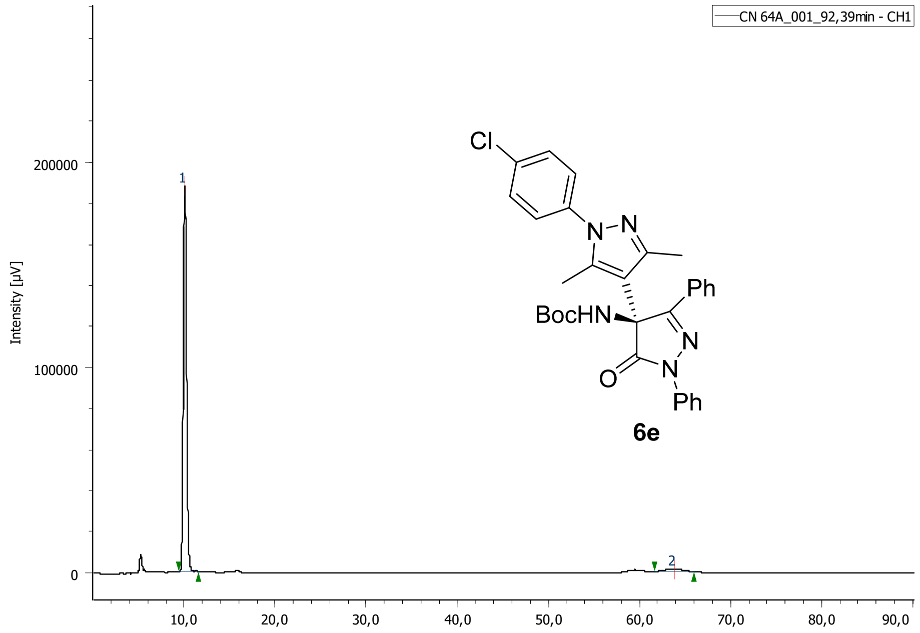

Peak Number	tR	Area	Height	Area%	Height%	Symmetry Factor
1	**10,075**	4591426	187683	**96,419**	99,392	1,124
2	**63,700**	170512	1149	**3,581**	0,608	1,007

Figure S97. HPLC Profile for **6e** compound. Scheme 5, er 96:4.

***tert*-Butyl (*S*)-(4-(3,5-dimethylisoxazol-4-yl)-3-methyl-5-oxo-1-phenyl-4,5-dihydro-1*H*-pyrazol-4-yl)carbamate (7a).**

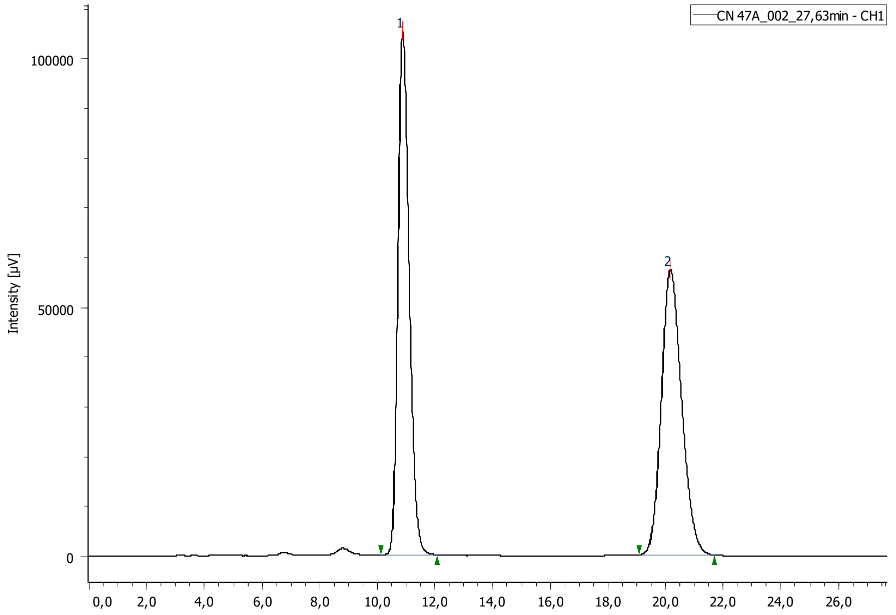

Peak Number	tR	Area	Height	Area%	Height%	Symmetry Factor
1	**10,867**	2829567	105132	**50,156**	64,746	1,257
2	**20,142**	2811934	57245	**49,844**	35,254	1,182

Figure S98. HPLC profile for **7a** (racemic).

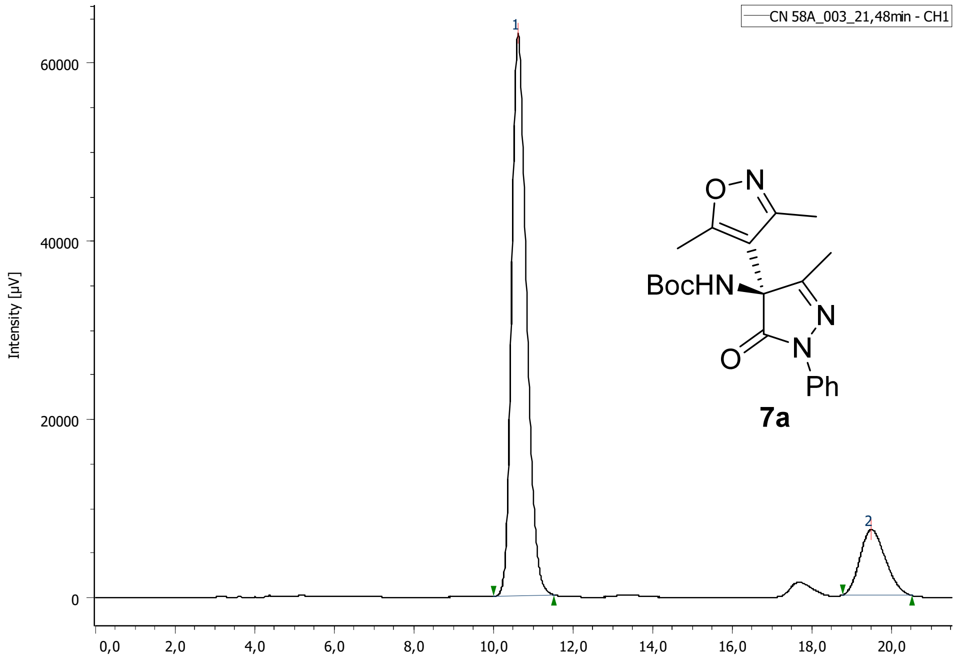

Peak Number	tR	Area	Height	Area%	Height%	Symmetry Factor
1	**10,608**	1618696	63025	**83,159**	89,543	1,271
2	**19,475**	327817	7360	**16,841**	10,457	1,195

Figure S99. HPLC Profile for **7a** compound. Scheme 5, er 83:17.

***tert*-Butyl (*S*)-(4-(3,5-dimethylisoxazol-4-yl)-5-oxo-1,3-diphenyl-4,5-dihydro-1*H*-pyrazol-4-yl)carbamate (7e).**

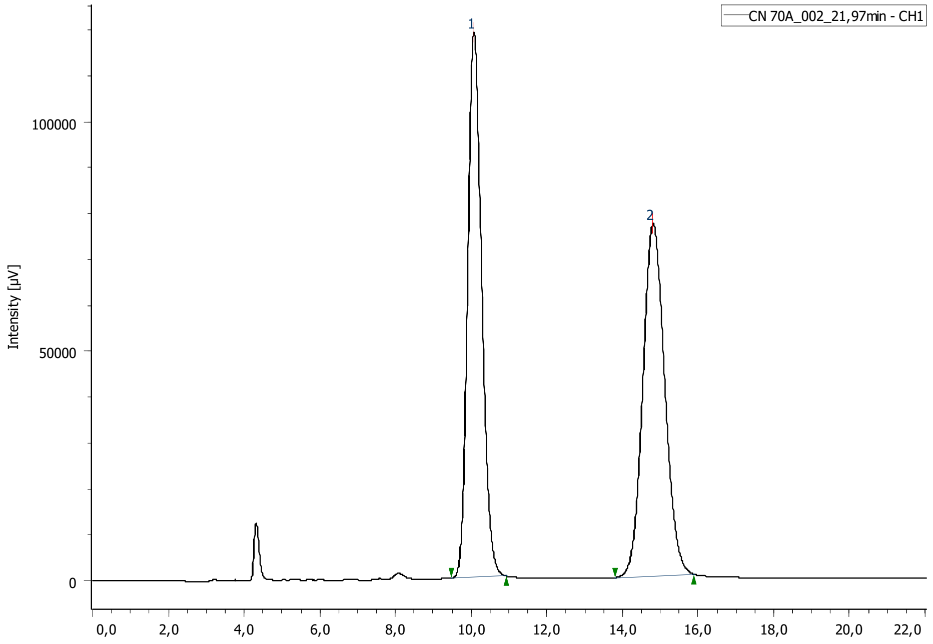

Peak Number	tR	Area	Height	Area%	Height%	Symmetry Factor
1	**10,067**	3046066	118497	**49,751**	60,739	1,178
2	**14,792**	3076553	76594	**50,249**	39,261	1,099

Figure S100. HPLC profile for **7e** (racemic).

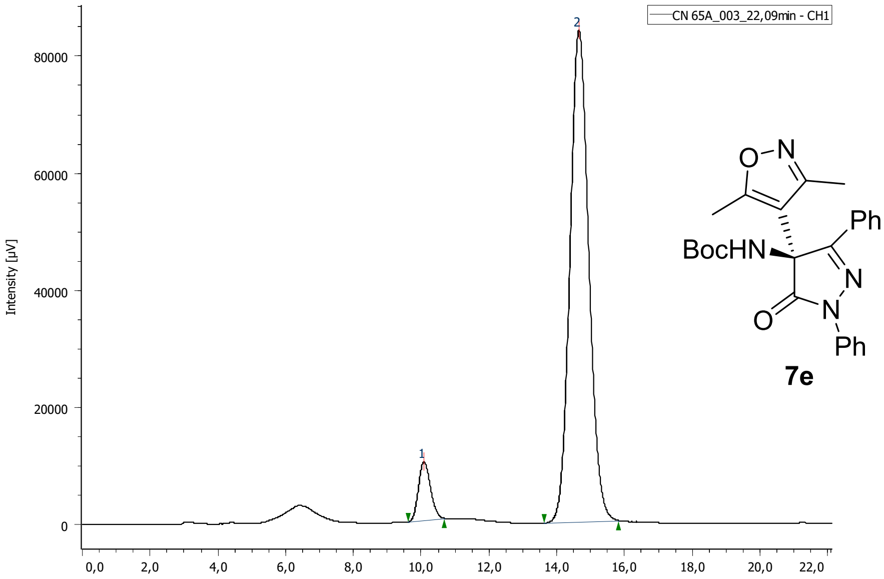

Peak Number	tR	Area	Height	Area%	Height%	Symmetry Factor
1	**10,075**	246531	10010	**7,067**	10,659	1,139
2	**14,633**	3242092	83894	**92,933**	89,341	1,109

Figure S101. HPLC Profile for **7e** compound. Scheme 5, er 93:7.
